# Alignment Modulates Ancestral Sequence Reconstruction Accuracy

**DOI:** 10.1093/molbev/msy055

**Published:** 2018-04-03

**Authors:** Ricardo Assunção Vialle, Asif U Tamuri, Nick Goldman

**Affiliations:** 1 European Molecular Biology Laboratory, European Bioinformatics Institute, Wellcome Genome Campus, Hinxton, United Kingdom; 2 Department of Biochemistry and Immunology, Federal University of Minas Gerais, Belo Horizonte, Minas Gerais, Brazil; 3 Department of Genetics and Molecular Biology, Laboratory of Human and Medical Genetics, Federal University of Pará, Belém, Pará, Brazil; 4 Research IT Services, University College London, London, United Kingdom

**Keywords:** ancestral sequence reconstruction, multiple sequence alignment, ancestral protein reconstruction, phylogenetic analysis, simulation

## Abstract

Accurate reconstruction of ancestral states is a critical evolutionary analysis when studying ancient proteins and comparing biochemical properties between parental or extinct species and their extant relatives. It relies on multiple sequence alignment (MSA) which may introduce biases, and it remains unknown how MSA methodological approaches impact ancestral sequence reconstruction (ASR). Here, we investigate how MSA methodology modulates ASR using a simulation study of various evolutionary scenarios. We evaluate the accuracy of ancestral protein sequence reconstruction for simulated data and compare reconstruction outcomes using different alignment methods. Our results reveal biases introduced not only by aligner algorithms and assumptions, but also tree topology and the rate of insertions and deletions. Under many conditions we find no substantial differences between the MSAs. However, increasing the difficulty for the aligners can significantly impact ASR. The MAFFT consistency aligners and PRANK variants exhibit the best performance, whereas FSA displays limited performance. We also discover a bias towards reconstructed sequences longer than the true ancestors, deriving from a preference for inferring insertions, in almost all MSA methodological approaches. In addition, we find measures of MSA quality generally correlate highly with reconstruction accuracy. Thus, we show MSA methodological differences can affect the quality of reconstructions and propose MSA methods should be selected with care to accurately determine ancestral states with confidence.

## Introduction

Given an ensemble of known sequences, ancestral sequence reconstruction (ASR) refers to methods used to recover the genetic sequence character states of their common ancestors. It has been used to study molecular evolution of photo-reactive proteins (Chang et al. 2002; Shi and Yokoyama 2003; Ugalde et al. 2004; Yokoyama and Takenaka 2004; Chinen et al. 2005; Yokoyama et al. 2008; Bickelmann et al. 2015), thermal stability of ancient proteins (Gaucher et al. 2003; Shimizu et al. 2007; Gaucher et al. 2008; Gouy and Chaussidon 2008; Akanuma et al. 2011; Perez-Jimenez et al. 2011; Akanuma et al. 2015; Busch et al. 2016), and evolution of viral proteins (Kaiser et al. 2007; Gullberg et al. 2010; Zinn et al. 2015). Extensive reviews of these topics are found in Liberles (2007), Ogawa and Shirai (2013), and Merkl and Sterner (2016).

Ancestral reconstruction begins with a hypothesis of how taxa descend from common ancestors in a tree-based structure or phylogeny. The taxa are represented as tips of the tree, progressively connected by branches to their common ancestors represented by the internal nodes of the tree. The common ancestor sequence of the entire sample of taxa is the root of the tree. Protocols for ASR usually involve four steps (Merkl and Sterner 2016): 1) selecting extant sequences, 2) building a multiple sequence alignment (MSA), 3) computing a phylogenetic tree, and 4) reconstructing ancestral sequences. Reconstruction quality is likely to depend on the age of the ancestors, the number of observed descendants and the use of sufficiently realistic evolutionary models.

Two main paradigms for ancestral state reconstruction exist: maximum parsimony (MP) and probabilistic methods, which include maximum likelihood (ML) and Bayesian reconstructions. Probabilistic methods use an explicit model of substitution, unlike the implicit model embedded in MP. These methods can also estimate confidence in each inferred ancestor, often expressed as the posterior probability of the data (Ashkenazy et al. 2012). Although ASR algorithms can tolerate a certain degree of phylogenetic uncertainty (Hanson-Smith et al. 2010), these methods can also introduce biases into reconstructions (Matsumoto et al. 2015). Species tree-aware models incorporating gene losses and duplications, and horizontal transfer events, have been shown to improve the performance of ASR under these conditions (Groussin et al. 2015).

Established ML reconstruction methods can be divided into two types: marginal reconstruction and joint reconstruction. Marginal reconstruction assigns a character state to a single node on the tree and averages over all possible ancestral states at each other node, whereas joint reconstruction assigns a set of character states to all ancestral inferred nodes on the tree simultaneously. Marginal reconstruction can be considered an approximation for joint reconstruction (Pupko et al. 2000). For “sequence-centric” tasks such as determining the gene or protein sequence of a single extinct ancestor, marginal reconstruction can usually be applied, whereas joint reconstruction has been recommended for “lineage-centric” tasks such as counting changes at specific sites (Yang 2007).

Multiple sequence alignment is a crucial step in ASR and can yield two different outcomes: structural or evolutionary homology (Tan et al. 2015). For evolutionary-based methods, sequence sites should be related through evolutionary history with shared direct ancestors. On the other hand, structural methods align sites involved in similar structural folding patterns even when they lack common evolutionary history (Westesson et al. 2012). However, the impact of different MSA tools on ASR is still unknown and seems to have been overlooked. Previous studies suggest alignment errors could promote significant biases in evolutionary reconstructions (Westesson et al. 2012). Consequently, some reconstruction protocols recommend manual refinement at the alignment step, such as removing or trimming difficult sequences to remove gaps in the MSA (Cole et al. 2013). Depending on the requirements of downstream analysis, these approaches may solve some problems. However, they also add subjectivity into the methodology (Anisimova et al. 2010).

Here, we investigate how several popular MSA tools (table 1) impact ASR without any manual intervention. Using simulated evolutionary trees and sequences, we measured the accuracy of reconstructions derived from each alignment tool in order to evaluate performance under different scenarios.

**Table 1.** Multiple Sequence Alignment Tools.

Name, Version	Description	Characteristics
Clustal Omega, v1.2.0	Based on seeded guide trees and HMM profile-profile techniques (Sievers et al. 2011).	Progressive approach;
		permits use of guide tree
FSA, v1.15.9	Builds a multiple alignment using only pairwise estimations of homology through a sequence annealing technique (Bradley et al. 2009).	Consistency-aware approach
MAFFT FFT-NS-2, v7.294b	Simple progressive method using a distance matrix based on shared k-mers (Katoh et al. 2002).	Progressive approach
MAFFT E-INS-i, v7.294b	Implements Fast Fourier Transforms to optimize protein alignments based on physical properties of the amino acids. This version uses local alignment with generalized affine gap costs (Altschul). It is applicable to sequences with several domains (Katoh and Standley 2013).	Consistency-aware approach
MAFFT L-INS-i, v7.294b	Implements Fast Fourier Transforms to optimize protein alignments based on physical properties of the amino acids. This version uses local alignment (Smith-Waterman). It is designed for sequences containing one alignable domain (Katoh and Standley 2013).	Consistency-aware approach
MUSCLE, v3.8.31	Multiple Sequence Comparison by Log‐Expectation that includes a refinement step where branches of the tree are repeatedly chosen and profiles from either side realigned (Edgar 2004).	Progressive approach;
		permits use of guide tree
PAGAN, v0.61	Phylogeny-aware progressive alignment algorithm that uses graphs to describe the uncertainty in the presence of characters at certain sequence positions (Löytynoja et al. 2012).	Phylogeny-aware approach;
		permits use of guide tree
PRANK, v150803	Probabilistic multiple alignment that uses evolutionary information for the placement of gaps and modeling of the substitution process (Löytynoja and Goldman 2008).	Phylogeny-aware approach;
		permits use of guide tree; program variant +F enforces patterns of insertions consistent with phylogeny

## Results

### Simulated Sequence Data Sets

We simulated protein sequence data sets under a variety of realistic evolutionary scenarios using a combination of several simulation parameters. We first generated an ensemble of phylogenetic trees under the birth-process varying 1) tree height, 2) sampling fraction, and 3) taxon count. Following Hanson-Smith et al. (2010), we chose ultrametric trees to add greater control on ASR conditions, avoiding biases introduced by different branch lengths since shorter branches could bias the ancestral state reconstruction. This removes uncertainty from the problem and makes effects at different depths in the trees more interpretable. Sampling fraction variation affects the tree shape (as shown in supplementary fig. S1, Supplementary Material online) and can be considered as modeling extinction, such that the sampling fraction is the probability of any species surviving extinction (Yang and Rannala 1997), or to model an investigator’s taxon-sampling strategy (Nee et al. 1994). Sampling fraction values were chosen to represent a variety of tree shapes covering realistic cases. A lower sampling fraction yields more “star-like” topologies. Tree height represents the expected number of substitutions per site from root to tip; we chose a tree height of 0.8 to reflect realistic cases from amniote tree estimations (derived from Ensembl Compara, Vilella et al. 2009), and also studied larger heights to show methods’ performance beyond this case.

On each tree, protein sequence evolution was simulated under the WAG model (Whelan and Goldman 2001) using two different indel rates. Parameter values were selected from previous studies to represent realistic scenarios of protein evolution (Whelan et al. 2003; Whelan et al. 2006; Levy Karin et al. 2015; Md Mukarram Hossain et al. 2015; see Materials and Methods for details). We tested indel rates of 0.01 and 0.05, inspired by observations in amniote (Westesson et al. 2012) and mammalian genes (Cooper et al. 2004). For each simulation, we recorded the simulated sequences at the tips, the true alignments, and the true ancestral sequence for every internal node.

Table 2 shows the range of values of simulation parameters used. In total, 72 scenarios were analyzed (36 tree configurations under two indel rates), incorporating a gradient of difficulty for MSA.

**Table 2.** Parameters for Data Simulations.

Parameter	Value
Number of taxa^a^	16 | 32 | 64
Tree sampling fraction^a^	0.01 | 0.25 | 0.99
Tree height^a^	0.8 | 1.0 | 1.2 | 2.0
Birth-death tree rates^a^	Birth: 6 Death: 3
Indel rate^b^^,c^	0.01 | 0.05
Root length^b^	408 aa
Substitution model^b^	WAG + Γ (α = 1.8, 4 categories)^d^
Indel length distribution^b^	Power law with constant factor of 1.7 and maximum length of 20

Note.—Data simulations were performed using the 72 combinations of the given parameters. Parameters separated by “|” represent values used in different combinations. For each combination, ten trees were generated using evolver (Yang 2007) and, for each tree, ten sequence data sets were generated using INDELible (Fletcher and Yang 2009).

a BD kernel density parameters for phylogenetic tree simulation (evolver).

b Parameters for protein sequence simulation (INDELible).

c Rates of insertion and deletion are relative to an average substitution rate of 1. Insertion and deletion rates are equal.

d + Γ: including rate variation as described by the gamma distribution (Yang 1994).

### Estimated MSAs and Ancestral Sequences

We aligned the tip sequences from the simulated data sets above using each MSA tool listed in table 1. Aligners allowing user-specified guide trees were additionally evaluated with this option using the true tree. We denote such use of an optional guide tree with an asterisk (e.g., PAGAN*).

The character states at ancestral nodes were reconstructed from each aligner’s MSA using FastML (Ashkenazy et al. 2012). The true alignment of sequences at the tips, as simulated, was used to establish a baseline. We specify the true tree, substitution model and rates used in the simulation during reconstruction in order to isolate the influence of MSA tools and avoid biases introduced by, for example, inaccuracies in phylogenetic inference methods (note this is independent of the use of the true tree as guide tree in MSA tools, which is evaluated separately).

### Reconstruction Accuracy on Different Scenarios

The accuracy of an internal node’s reconstructed sequence to its corresponding true sequence was measured using a score based on the method of Paten et al. (2008). The score ranges from zero to one, representing the proportion of pairwise aligned sites that are correctly aligned; a perfect match has a score of one (see Materials and Methods for further details).

**Fig. 1.**
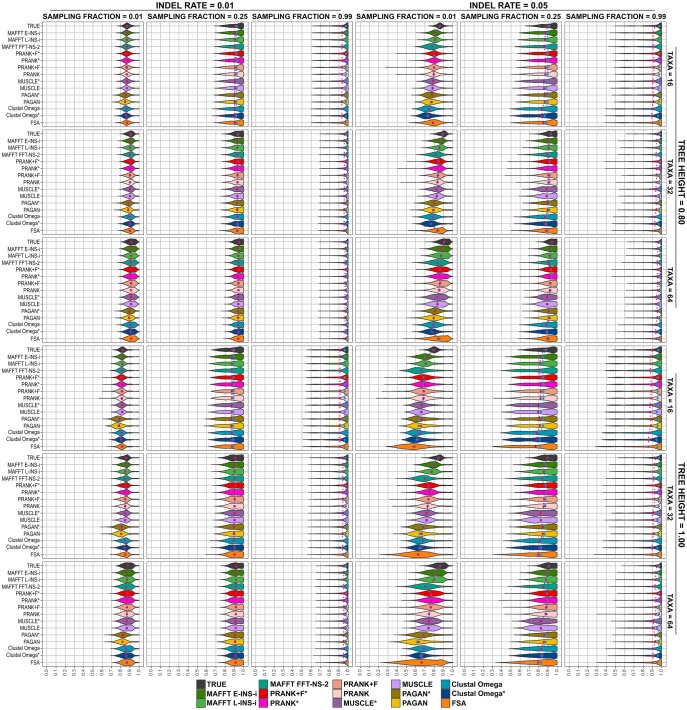
Reconstruction accuracies of MSA tools for simulated scenarios under tree heights of 0.8 and 1.0. Plots show the overall accuracy distribution for each parameter combination using tree heights of 0.8 and 1.0. Blue dots indicate the median, and red dots indicate the mean.

We first analyzed the overall accuracy trends of each MSA tool for each scenario. Figure 1 shows distributions of accuracies for tree heights 0.8 and 1.0, recorded for each tool over all reconstructed internal nodes and including all sequences and trees replicates (100 replicates for each scenario, comprising ten tree replicates with ten alignments simulated for each tree). Therefore, the number of nodes in each distribution is equal to the number of internal nodes in the rooted tree (#taxa—1) multiplied by 100. We found many conditions where ASR scored with high accuracy (distributions concentrated to the right on the *x*-axis) and few differences between methods. At sampling fraction 0.99, all methods have excellent and virtually equal performance (*P* value < 0.01, supplementary table S1, Supplementary Material online). Reducing the sampling fraction to 0.25 decreases the overall accuracy slightly, but results are still similar compared with the baseline (reconstruction using the true alignment). Differences become evident with sampling fraction of 0.01, indel rate of 0.05, and tree height of 1.0, and particularly when these difficult conditions are combined. In such cases, we start to observe clear differences between tools, with accuracies from estimated MSAs considerably lower than the true alignment, and some tools presenting particularly low accuracies for some ancestral nodes, especially FSA.

**Fig. 2.**
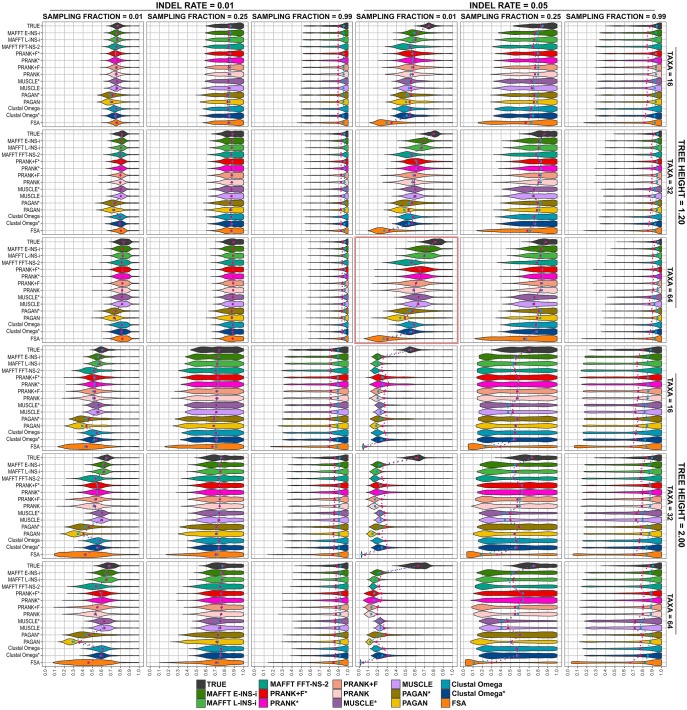
Reconstruction accuracies of MSA tools for simulated scenarios under tree heights of 1.2 and 2.0. Plots show the overall accuracy distribution for each parameter combination using tree heights of 1.2 and 2.0. Blue dots indicate the median, and red dots indicate the mean. Highlighted plot (red box) indicates the scenario with 64-taxon trees, tree height 1.2, sampling fraction 0.01, and indel rate 0.05, further explored in figures 4–6 and 8.

Under more challenging simulation conditions, we noticed the intensification of trends induced by each MSA tool. Figure 2 shows the accuracy distributions for simulations with tree heights of 1.2 and 2.0, where we find methods performing poorly. In the most difficult cases (e.g., indel rate 0.05, tree height 2.0, and sampling fraction 0.01), we see accuracies generally below 0.3 for all MSA methods, considerably below the baseline values obtained using the true alignment (*P* value < 0.01, supplementary table S1, Supplementary Material online). In general, we observe simulations with sampling fraction of 0.99 (later divergences) are more easily solvable: even in the most challenging situations (indel rate 0.05 and tree height > 1.0), reconstruction accuracies are high (>0.7 on average). A lower indel rate of 0.01 also results in good performance (except when combined with the most difficult tree height of 2.0 and sampling fraction of 0.01), as does lower tree height. Increasing the number of taxa leads to a modest improvement in accuracies overall.

Accuracy as a function of individual parameter choices, summarized over all other conditions and all aligners, is shown in supplementary figure S3, Supplementary Material online. Taken in combination with figures 1 and 2, these confirm our expectations about which features make a given ASR problem more of less difficult. Given the increased information available from having more extant sequences, trees with more taxa display a slightly higher reconstruction accuracy. The sampling fraction drastically affects accuracy, with a higher fraction (later divergences) yielding more accurate reconstructions. This reflects closeness of the internal nodes and leaf sequences making alignment easier. Tree height is also a critical variable, with longer trees (more divergent sequences) presenting more difficult scenarios and lower reconstruction accuracies. The lower indel rate of 0.01 produced higher accuracies than the rate of 0.05: sequences with few indels are clearly easier to align, in turn leading to better ASR performance.

**Fig. 3.**
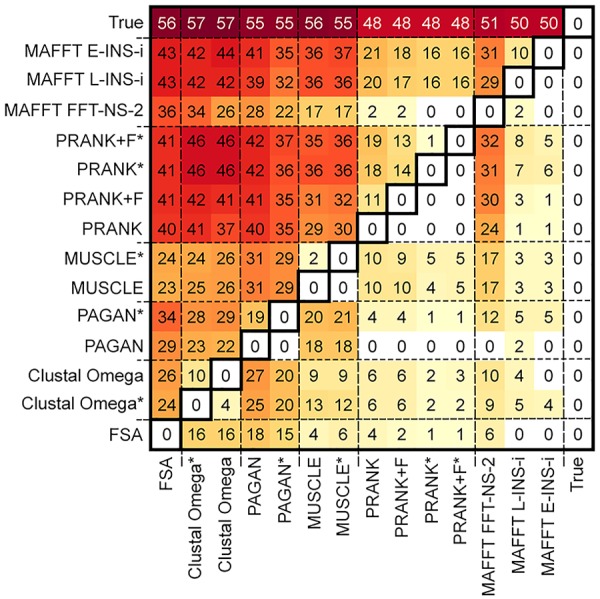
Number of scenarios with statistically significant differences in overall accuracy between each MSA. The reconstruction accuracies obtained by each MSA tool in 72 scenarios with varying parameter configurations were compared pairwise using a Mann–Whitney–Wilcoxon test. Figure shows counts of scenarios with significant differences (FDR adjusted *P* value < 0.01), where the entry in the *i*-th row and *j*-th column shows the number of times method *i* was better than method *j* (higher median accuracy).

Pairwise comparisons between MSA methods allowed us to calculate the number of scenarios under which the MSA tools differed significantly, providing an overview of their performance across multiple conditions (fig. 3). In instances where differences were observed, reconstructions using the true alignment (baseline) led to better results (higher median accuracies) than MSA tools (fig. 3, top row). Among the MSA tools, PRANK using the guide tree (PRANK* and PRANK + F*) achieved the best results by this measure, showing significant differences when compared with the baseline in 48 of the 72 scenarios simulated (67%). PRANK without a guide tree (PRANK and PRANK + F) and the MAFFT aligners performed similarly to PRANK* variants. Clustal Omega performed worst, showing differences in 57 of 72 scenarios (79%); FSA, PAGAN and MUSCLE gave similar results to Clustal.

Applying the same comparisons between results from different MSAs indicated that methods were more similar to each other than they were to the baseline reconstruction using the true alignment (fig. 3). Different variants of the same MSA tool tended to perform similarly (notably, PRANK* and PRANK + F* differed in only 1 scenario, and MUSCLE and MUSCLE* in only 2). We also found similarities between tools; for example, MAFFT E-INS-i showed significantly different accuracy from PRANK + F in only 19 scenarios (∼26%). However, when these differences were present, MAFFT was better in 18 of them. The same was observed with other combinations. Generally, MAFFT’s INS-i variants and PRANK variants performed better than other tools; FSA performed worst. Finally, some tools displayed balanced trends; for instance, MAFFT FFT-NS-2 and MUSCLE were significantly different in 34 scenarios (∼47%), and each tool was better in half of them.

### Reconstruction Accuracy Variation along Trees

**Fig. 4.**
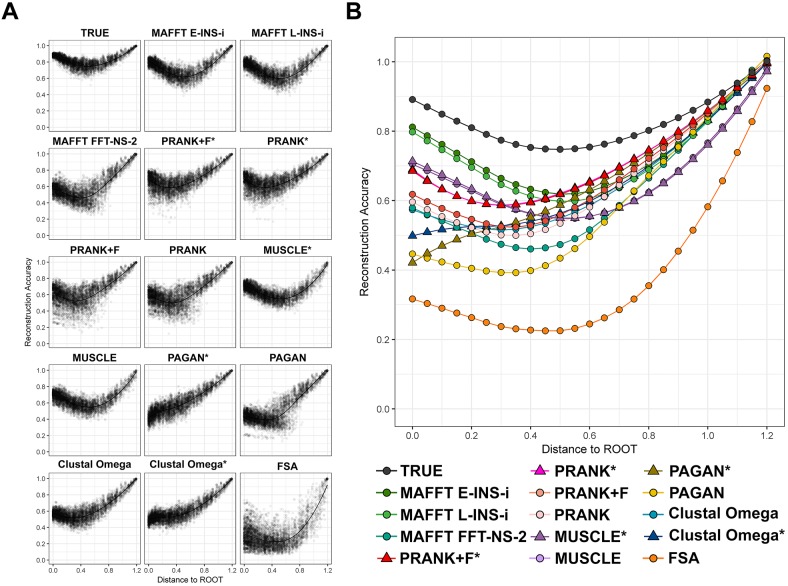
Reconstruction accuracy by distance to root. Reconstruction accuracy at different distances from the root using simulation parameters of 64 taxa, tree height 1.2, sampling fraction 0.01 and indel rate 0.05. (*A*) Scatter plots of accuracies for each MSA. (*B*) Combined chart showing the locally weighted scatterplot smoothing (LOESS) of average reconstruction accuracy by distance to root for each MSA tool.

To further explore method performance, we concentrated on a single set of simulation conditions that exhibited contrasting results, with some good reconstructions but substantial differences between MSA tools. We examined simulations with 64-taxon trees, tree height 1.2, sampling fraction 0.01, and indel rate 0.05 (fig. 2, highlighted plot). Figure 4 shows reconstruction accuracy as in the corresponding summary plot within figure 2, but now stratified along the true tree, according to each node’s distance from the root (the corresponding figures for other simulation conditions are available in the supplementary additional file S1, Supplementary Material online). Analyzing the accuracies of all reconstructed internal nodes (fig. 4*A*), we observed that FSA, PRANK + F, PRANK, PAGAN, and MAFFT FFT-NS-2 exhibited highest variation in reconstruction accuracy with more dispersed accuracies along trees (supplementary table S2, Supplementary Material online). Supplying the true tree as the guide tree to tools permitting this option (PRANK*, PRANK + F*, PAGAN*, Clustal Omega*, and MUSCLE*) reduced this variation.

Comparing the average accuracy along the tree for each MSA tool (fig. 4*B*), we observed that, with the exception of FSA, all aligners performed similarly well for ancestors close to the tips of the tree (to the right of *x*-axis) compared with reconstruction using the true alignment (baseline). The accuracies decrease moving along the tree (moving left on the *x*-axis, i.e., towards the root)—deeper ancestors are harder to reconstruct accurately—but tend to increase again near the root (with the exception of Clustal Omega* and PAGAN*). This increase is explained by the influence of the information conveyed by the denser-sampled nodes concentrated in the root region, which is a consequence of the sampling fraction of 0.01 (for sampling rates of 0.25 and 0.99, the accuracy decreased monotonically nearer to the root; see supplementary fig. S4, Supplementary Material online).

Overall, the differences between MSA tools observed in figure 4*B* showed MAFFT E-INS-i and MAFFT L-INS-i to have the best performance in nodes close to the root with an accuracy of approximately 0.8; MUSCLE*, MUSCLE, PRANK* and PRANK + F* have accuracies around 0.7; PRANK, PRANK + F, Clustal Omega and MAFFT FFT-NS-2 have accuracies near 0.6. For intermediate depth nodes (slope change region, around distance 0.4), we see accuracies ranging from 0.5 to over 0.6 for most of the MSA tools, except for FSA (accuracy of approximately 0.2), PAGAN (0.4) and MAFFT FFT-NS-2 (around 0.45). For nodes close to the tips (distance to root 1.0–1.2), nearly all tools performed well, with accuracies higher than 0.8. MUSCLE variants were slightly worse, with accuracies around 0.05 below other tools in this region, and FSA had the worst results, rapidly decreasing in accuracy to below 0.6. These differences show not only how each tool behaves in relation to the cumulative error introduced in each level along the tree (from root to tip, along the *x*-axis of fig. 4), but also the capability of correction from the reconstruction method in the final stages when there is more information available. Despite overall similar performances at initial nodes near the tips, the discrepancy caused by the MSA tool in the most ancestral nodes is shown to be considerable.

Using the true tree as guide tree for MSA led to intriguing results. For PRANK variants, using the guide tree consistently improved the accuracy along all trees (Mann–Whitney–Wilcoxon, *P* value < 0.01). In contrast, MUSCLE and MUSCLE* gave virtually the same results, showing no considerable differences when using the guide tree. For Clustal and PAGAN the use of the guide tree improved accuracies in almost all regions, but worsened performance considerably for nodes close to the root.

### Biases for Insertion and Deletion in Reconstructed Sequences

**Fig. 5.**
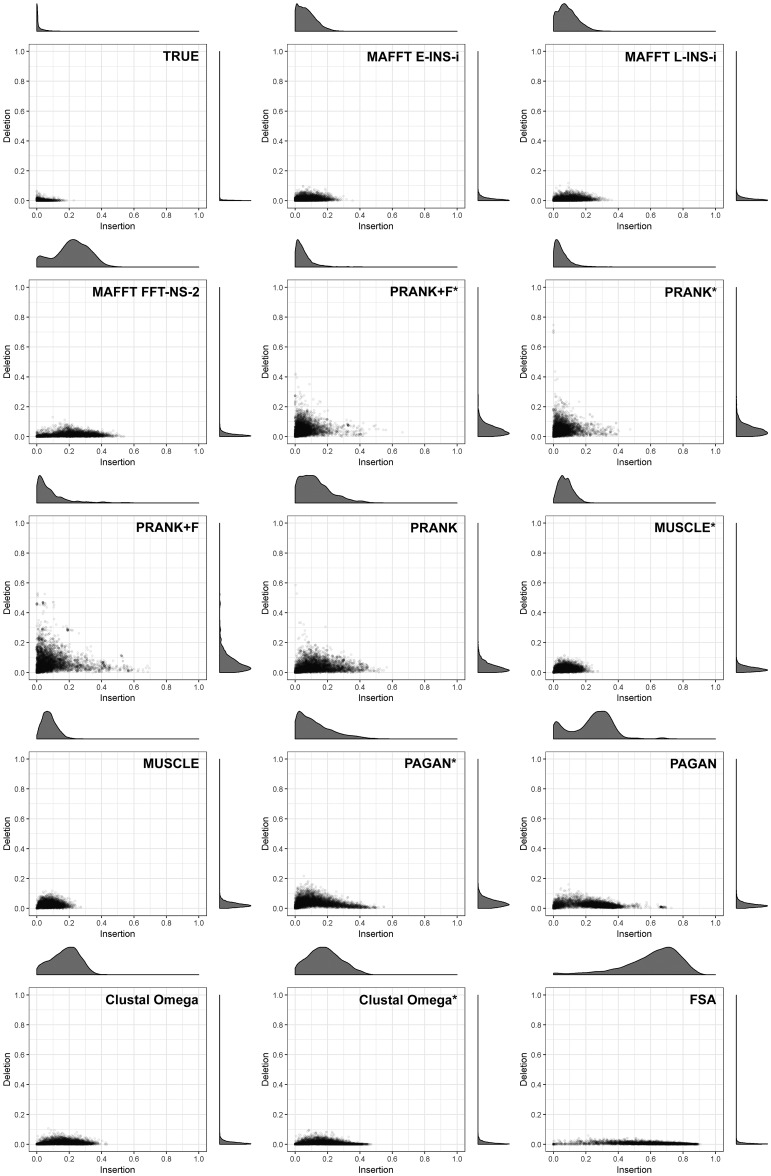
Distributions of insertion and deletion error metrics. Scatterplots show insertion and deletion error metrics for different MSA methods, based on the simulation parameters: 64 taxa, tree height 1.2, sampling fraction 0.01, and indel rate 0.05. Insertions are shown on the *x*-axis, deletions on the *y*-axis. Density distribution for each axis is also plotted.

We analyzed the contribution of insertion and deletion errors to the accuracy measure to discover specific biases in the MSA tools. Insertion and deletion errors are included in the accuracy measure (see Materials and Methods) and represent the percentage of residues present (insertion) or not present (deletion) in the reconstructed ancestral node compared with the true sequence. Recall that correct ASR would result in insertion and deletion error scores of 0 (see above). Again, concentrating on simulation conditions where the MSA methods had contrasting results (64 taxa, tree height 1.2, sampling fraction 0.01, and indel rate 0.05), we discovered biases in all tools, including reconstructions based on the true alignment (fig. 5). Deletion errors (plotted on the *y*-axis) were low for most of the tools, with PRANK variants showing worst results. PRANK + F had slightly higher percentage of error attributed to deletions compared with PRANK, and using the guide tree resulted in similar distributions. PAGAN* also showed deletion errors marginally higher than other tools, but lower than PRANK.

For insertion errors (fig. 5, *x*-axis), we observed considerable biases in some tools. By this measure, PRANK + F*, PRANK*, PRANK + F, MUSCLE variants, and MAFFT’s INS-i variants showed best results, all with overall insertions errors below 0.2 (with differences in dispersion). Other MSA tools displayed a strong bias towards insertions, particularly FSA, which yielded insertion errors of >0.8 (i.e., 80% of pairwise alignment length composed by gaps in true sequence).

**Fig. 6.**
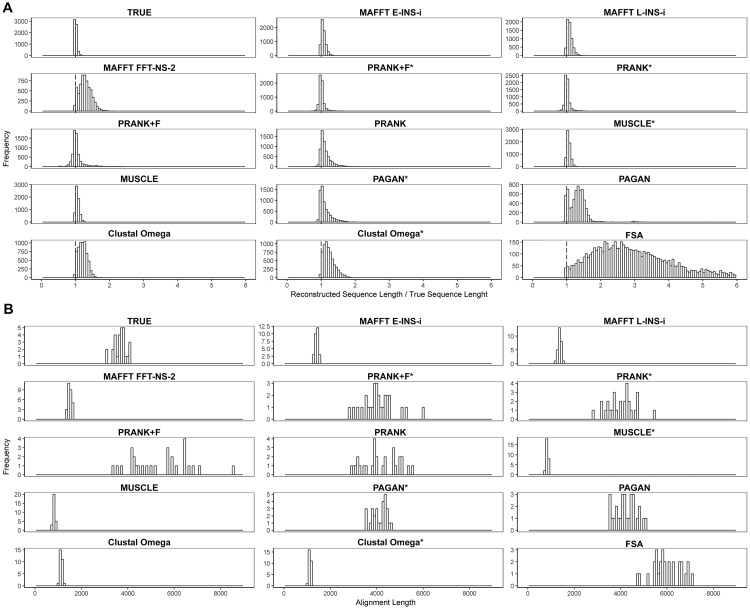
Reconstructed sequence lengths and alignment lengths. Distributions of sequence and alignment lengths for each alignment method (simulation parameters: 64 taxa, tree height 1.2, sampling fraction 0.01, indel rate 0.05). (*A*) Distribution of ratios of reconstructed to true sequence lengths measured for all reconstructed nodes. Values higher than one represent reconstructed sequences longer than expected. (*B*) MSA length distributions for each method measured for each scenario replicate (100: ten trees, and ten alignments for each tree).

The bias towards insertions results in longer reconstructed sequences (fig. 6*A*). However, looking at the multiple alignment lengths from each tool from all scenario replicates (100 replicates: ten trees, and ten sequences for each tree), the impact of any balance between insertions and deletion errors is unclear (fig. 6*B*). Although virtually all MSA tools overestimate the number of insertions compared with deletions, alignment lengths do not show correlation with ancestral sequence lengths. Overall, shorter than expected alignments, such as those from Clustal, MUSCLE and MAFFT, did not induce shorter reconstructions. Such differences may be due to a given method’s tendency to balance two types of error: too many insertions and overalignment. Under such conditions, sparse alignments are expected (see true alignment, supplementary fig. S5, Supplementary Material online) and PRANK, PAGAN, and FSA display this property. However, FSA gap regions may be a consequence of how it penalizes overalignments. Since FSA (by default) stops aligning characters when the probability that a character is aligned is equal to the gapped probability, it leads to incorrect indel placement (resulting in underalignments). In this context, alignments from PRANK variants were more consistent with simulations.

Although the alignment length may give some insights into the performance and utility for downstream analyses of different MSA methods, its accurate estimation has no particular value itself. Rather, the ability of the MAFFT INS-i, PRANK, and MUSCLE variants to give individual inferred ancestral sequences with lengths most closely resembling true values is an important measure of their superior performance.

### Comparison of Reconstruction Accuracy and MSA Quality Measures

**Fig. 7.**
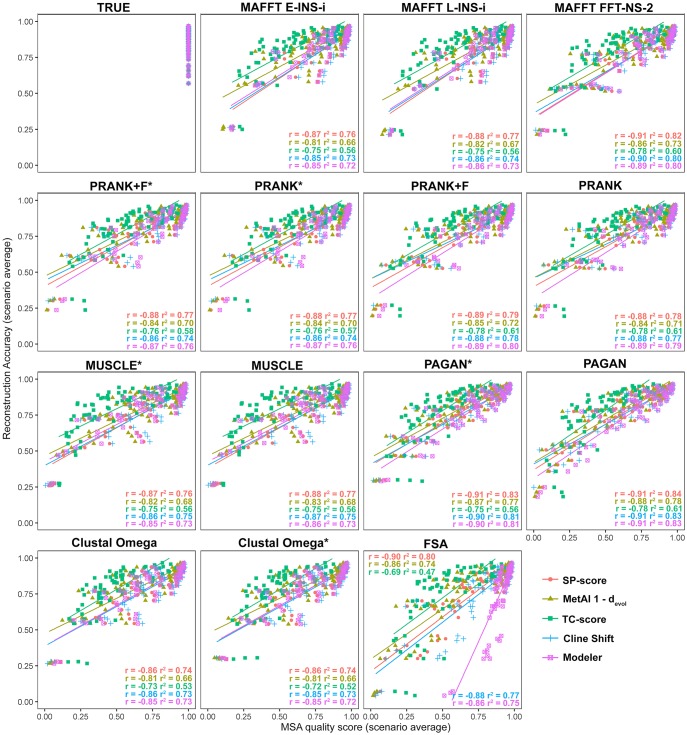
Relationship between reconstruction accuracy and MSA quality metrics. Average reconstruction accuracy and average MSA quality scores calculated for each simulated scenario (72 scenarios) using each MSA tool. MSA quality metrics described in the text are computed by comparing the MSA with the true simulated alignment. *MetAl* was used under the *d*_evol_ metric which corresponds to a dissimilarity score, so values were subtracted from 1 for ease of comparison. (*r*: Pearson’s correlation; *r*^2^: coefficient of determination).

We compared reconstruction accuracy with measures of MSA quality. MSA quality measures were calculated using the *d*_evol_ measure from *MetAl* (Blackburne and Whelan 2012) and the following scores from Q-Score (Edgar 2004): Developer score (also called the SP-score, for sum-of-pairs), Modeler score, Total Column score, and Cline Shift score. As the *MetAl* score represents an error metric (ranging from 0, representing no error, to 1, maximum error), values were subtracted from 1 to produce an accuracy measure, more-readily related to the other metrics. Figure 7 shows plots of reconstruction accuracy against the measures of MSA quality for all 72 simulation conditions. For each scenario, we considered the average reconstruction accuracy (covering all nodes within all scenario replicates) and the average MSA quality of all replicates. Overall the MSA quality measures produced similar results, showing good correlation with reconstruction accuracy with coefficient of determination values (*r^2^*) typically higher than 0.75 for most of MSA tools and quality measures. An exception was the TC score, which showed lower correlation (*r^2^* around 0.60) when compared with other quality measures.

Only small differences were observed for specific aligners. The most notable of these is the Modeler score, which yielded anomalously high values for FSA when compared with other measures and aligners (fig. 7, FSA plot). This specific discrepancy is a consequence of how the Modeler score is normalized, favoring situations of underalignment and neglecting indel regions for normalization. As FSA produces long and sparse alignments, even a few correctly inferred homologies, when divided by few aligned regions, leads to higher scores. For this reason, the Modeler score is usually combined with the SP-score (Developer) (Wang and Dunbrack 2004).

**Fig. 8.**
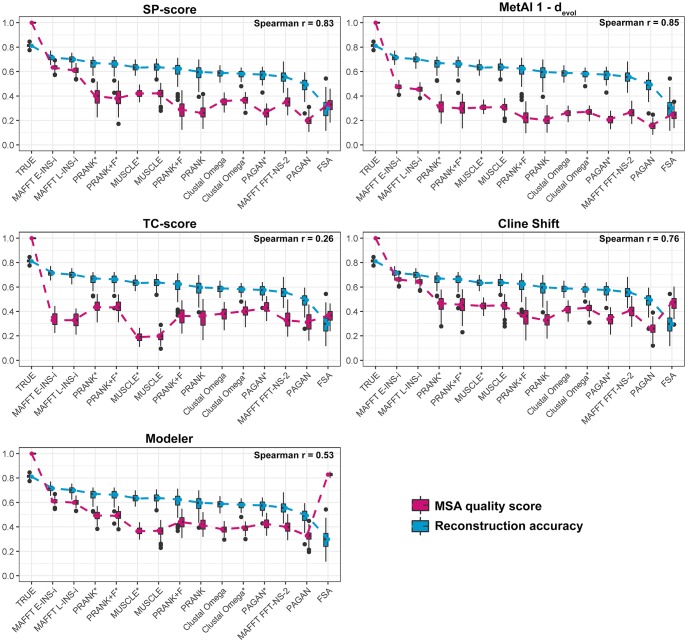
MSA quality scores compared with reconstruction accuracy over different MSA tools. Differences of quality measures between MSA tools under simulation parameters of 64 taxa, tree height 1.2, sampling fraction 0.01, and indel rate 0.05. MSA quality scores (pink) represent values for each scenario replicate (ten trees and ten alignments for each tree). In all plots, reconstruction accuracies (blue) are shown for comparison, representing the expected behavior in terms of differences between tools. Values of reconstruction accuracies were measured as averages of all reconstructed node accuracies in each replicate, and are the same in each chart. MSA tools are ordered by reconstruction accuracy means (best to worst). Spearman rho correlations between MSA quality scores and reconstruction accuracies are shown for each metric. *MetAl* scores are shown as 1 − *d*_evol_, to produce a similarity measure.

Despite the generally good overall correlations between MSA quality measures and reconstruction accuracy within specific MSA tools, the comparison between metrics over different alignment tools, especially in contrasting scenarios, shows some alignment quality metrics orthogonal to reliable reconstruction. Figure 8 shows the average reconstruction accuracy and MSA quality measures for simulations with 64-taxon trees, tree height 1.2, sampling fraction 0.01 and indel rate 0.05 (the same parameters studied previously, figs. 4–6). We observed differences in reconstruction accuracy amongst tools (in blue) are not captured for some quality metrics (in pink). Other than the Modeler/FSA discrepancy, other differences can be discerned, especially the TC-score presenting unexpected results for many MSA tools. Such differences show how well each quality metric can capture the differences observed with the reconstruction accuracies. Thus, under these simulation conditions (considered challenging for reconstruction), the TC-score yields the worst predictions of ASR accuracy (correlation of 0.26), whereas the *MetAl* (1 − *d*_evol_) and SP-score measures performed best (correlation > 0.85).

### Alternative Indel Parameters

In our primary analysis, we simulated sequences using indel rate parameters of 0.01 and 0.05. However, analyses of mammalian and bacterial orthologs from the OrthoMam (Douzery et al. 2014) and COG (Tatusov et al. 2003) databases suggest an indel rate of 0.02 and a power law distribution constant of 1.125 for mammalian proteins. Estimates from COG suggest an indel rate of 0.125 and power law distribution constant of 1.3 (Levy Karin et al. 2015). Therefore, we simulated data with these indel rates on 32-taxon trees, tree height of 1.0 and sampling fraction of 0.01 and 0.99 (supplementary material—additional file S2, Supplementary Material online). The maximum indel length allowed was 50 amino acid residues. The results for the mammalian rates were similar to those obtained with the parameters of indel rate of 0.05, with slightly better accuracies. The indel parameters estimated from COG orthologs represent far more challenging conditions. No MSA tool achieved good reconstruction accuracies using the higher indel parameter value (0.125), with accuracies in most ancient nodes below 0.2. Accurate reconstructions were obtained near the tips. The high indel rate inferred by COG could be due its generalist aspect, which, by definition, includes several groups of orthologs (Trachana et al. 2011; Douzery et al. 2014). Therefore, reliable reconstructions of the most ancestral nodes are not possible; this does not represent a viable case for ASR of proteins.

## Discussion

We tested the impact of MSA tools on ancestral state reconstruction accuracy using amino acid sequences simulated under various realistic conditions (tables 1 and 2). We found undemanding conditions (low indel rate, high birth-death process sampling probability, and low tree height) result in effectively no differences between any alignment methods, with reconstruction accuracy as good as using the true alignment, frequently permitting near-perfect ASR (fig. 1). Increased taxon sampling does improve accuracy.

However, we found the choice of MSA method can impact on ASR under more demanding conditions. Our analyses reveal that some factors, like tree topologies and indel rates, have a more significant impact on ASR than others (e.g., number of taxa). Altering tree shape by lowering the sampling fraction, increasing the number of substitutions per site and increasing indel rates all reduce reconstruction accuracy; in these difficult cases, differences between MSA tools were revealed (figs. 1 and2). MAFFT consistency aligners (MAFFT E-INS-i and MAFFT L-INS-i) and PRANK variations performed best overall, frequently with indistinguishable reconstruction accuracies. No one method performed uniformly better over all conditions tested. There were also differences in the MSA tools’ ability to reconstruct ancestral sequences at different depths within phylogenies (figs. 3 and4). The MAFFT consistency algorithms do not employ an explicit model of indels when aligning sequences but this does not negatively impact the resulting reconstruction accuracy of sequences simulated under a high indel rate. The progressive aligners MUSCLE, Clustal Omega and MAFFT FFT-NS-2 had lower accuracy performance. PAGAN performed poorly in some cases, especially at lower indel rate, but using a guide tree improved reconstruction accuracy. FSA performed worst in most contexts, particularly in the more challenging cases. We found FSA was especially sensitive to indel rate.

There was a notable bias towards insertions by all aligners (fig. 5). A slight tendency to overestimate insertions, even when using the true alignment, suggests some influence of the FastML ASR algorithm. Only PRANK variations (specifically PRANK*, PRANK + F, and PRANK + F*) demonstrated an ability to balance insertions and deletions and to estimate ancestral sequences of approximately correct length (fig. 6). However, the insertion bias we revealed was considerable among all other aligners, especially FSA. This inherent bias may underlie its poor performance in ASR accuracy. Since FSA by default tries to maximize specificity, indel events are inferred through a maximum parsimony interpretation that minimizes “gap openings” leading to underalignment (Bradley et al. 2009). In contrast, phylogeny-aware methods like PRANK and PAGAN deal much more carefully with this issue; indel events are treated using the phylogenetic information (Löytynoja and Goldman 2005). It was expected that this approach would invariably lead to better reconstructions. However, the good performance obtained by progressive and consistency aligners (typically prone to overalignment) in comparison to phylogeny-aware approaches exposes the robustness of the ancestral reconstruction method.

The problem between overalignment and underalignments has been extensively discussed (Löytynoja and Goldman 2005; Schwartz and Pachter 2007; Löytynoja and Goldman 2008; Bradley et al. 2009; Redelings 2014; Katoh and Standley 2016). We observed methods that tended to overalign (Clustal, MAFFT, MUSCLE), to underalign (FSA, PRANK + F) or were largely unbiased (PAGAN, other PRANK variants). The optimal approach will depend on multiple factors, including the final purpose of analysis, similarities between sequences and computational costs. When gapped regions are not of interest (e.g., BAliBASE: Bahr et al. 2001), filtering methods and manual intervention are usually applied (Castresana 2000; Capella-Gutiérrez et al. 2009; Penn et al. 2010; Chang et al. 2014). Many studies of ASR take this approach (Perez-Jimenez et al. 2011; Cole et al. 2013; Gumulya and Gillam 2017) which is not recommended due the introduction of subjectivity into the analysis (Anisimova et al. 2010). In our study, we used controlled simulation scenarios, and evaluated the ability of MSA tools to deal with homologous sequences without any additional interference. Under such conditions, we noticed the reconstruction algorithm deals better with overalignment than underalignment conditions. Also, the robustness of reconstructions regarding other factors such as taxon sampling (Randall et al. 2016) was confirmed in our simulations.

Multiple sequence alignment quality scores can assist researchers when choosing algorithms and tools for ASR accuracy. We tested five different alignment quality scores and showed they were highly correlated with reconstruction accuracy across different scenarios (fig. 7). However, some metrics did not capture the complexity within specific scenarios. The TC and Modeler scores were less useful than other measures to inform on reconstruction accuracy. On the other hand, *MetAl d*_evol_ and SP-score achieved good correlation overall (fig. 8).

Our results are, of course, limited by the simulations we could perform. Alternative tree topologies may change MSA behavior: for example, very unbalanced trees could amplify biases. In addition, other ASR methods and different runtime configurations may impact the outcome. We measured reconstruction accuracy using a “neutral” character comparison that did not account for amino acids’ properties or other evolutionary trends. The MSA methods themselves use a variety of amino acid substitution matrices during the alignment process. Therefore, using an accuracy score that utilised a particular amino acid substitution matrix could bias the results—a neutral measure does not seem better or worse than other criteria. There are many complex evolutionary processes at work in real data. For example, gene tree/species tree discordance, gene gain and loss, horizontal gene transfer, and unequal rates and sizes of insertions and deletions could all complicate MSA and ASR methods. In principle, MSA methods accounting for these phenomena could improve their performance, not least with respect to ASR. In our simulations, we were specifically interested in the effects of MSA on ASR and, therefore, avoided other complicating factors. In summary, results such as ours can help to identify parameter combinations that delineate reliable and accurate reconstruction limits. Although certain MSA tools introduce bias, some biases may not be relevant for common use cases (e.g., easily solvable scenarios). In more-challenging situations, MSA methods must be chosen with caution to provide reliable reconstructions of ancestral states.

## Materials and Methods

### Simulating Phylogenetic Trees

Ultrametric phylogenetic trees were simulated using *evolver* from the PAML suite (Yang 2007) under a birth-death process (Yang and Rannala 1997). Trees with 16, 32, and 64 taxa were generated with sampling fraction of 0.01, 0.25, and 0.99 and tree heights of 0.8, 1.0, 1.2, and 2.0 (table 2). In total, we used 36 combinations of parameters and simulated ten trees for each combination, resulting in 360 phylogenetic trees.

### Simulating Protein Sequences

For each tree, ten sets of amino acid sequences were simulated using INDELible (Fletcher and Yang 2009; option “AMINOACID 1”). The length of the ancestral sequence at the root of the trees was 408 sites, and substitutions were modelled using the WAG substitution model (Whelan and Goldman 2001) with gamma-distributed among-site rate variation (α = 1.8 and 4 categories) (Yang 1994). Insertion and deletion length distributions were specified as Zipfian (i.e., a power law distribution) with the constant factor of 1.7, in accord with empirical estimations (Benner et al. 1993; Gu and Li 1995; Zhang and Gerstein 2003; Yamane et al. 2006; Cartwright 2009), and not permitting indels longer than 20 amino acid residues (Md Mukarram Hossain et al. 2015).

### Multiple Sequence Alignment Tools

We evaluated aligners that use a variety of different approaches, comprising the progressive aligners Clustal Omega (Sievers et al. 2011), MUSCLE (Edgar 2004), and MAFFT FFT-NS-2 (Katoh et al. 2002); the consistency aligners FSA (Bradley et al. 2009), MAFFT E-INS-i and MAFFT L-INS-i (Katoh and Standley 2013); and the phylogenetically aware aligners PAGAN (Löytynoja et al. 2012) and PRANK (Löytynoja and Goldman 2008) (table 1). We evaluated all aligners with their default parameters. PRANK was evaluated with and without the “permanent insertions” option (Löytynoja and Goldman 2008), denoted PRANK + F and PRANK, respectively. MSA tools allowing the stipulation of a guide tree were additionally evaluated with this option using the true tree.

### Reconstructing Ancestral Sequences

We calculated ASRs using FastML (Ashkenazy et al. 2012). Marginal reconstruction was used to simulate cases of interest in reconstructing ancestral roots as advised by Pupko et al. (2000). We used the true tree and branch lengths, WAG substitution model and among-site rate variation in accordance with simulation conditions. Indel reconstruction was calculated using maximum likelihood, and we used the default indel probability cut-off (i.e., the most likely character states in the ancestral nodes were reported only in positions inferred to be nongapped with probability ≥ 0.50).

### Measuring Reconstruction Accuracy

Reconstruction accuracy was evaluated as in Paten et al. (2008). Reconstructed internal node sequences were pairwise aligned with the corresponding true ancestral sequences using MUSCLE. Three error scores were calculated:

1. Insertion error: the number of residues present in the reconstructed sequence but absent in the true sequence, divided by the length of the alignment.
2. Deletion error: the number of residues present in the true sequence but absent in the reconstructed sequence, divided by the length of the alignment.
3. Substitution error: the number of mismatched residues divided by the length of alignment.

All error measures range from 0 to 1, with error equal to 0 being the ideal case with no differences between reconstructed and true sequences according to the metric. Subtracting the sum of these error scores from 1 provides a measure of overall accuracy, representing the proportion of the pairwise alignment sites at which a correctly aligned residue appears (e.g., see supplementary fig. S2, Supplementary Material online). Note we are not inferring evolutionary events here—there will have been true indels and substitutions in the evolutionary histories—but using the terms insertion, deletion, and substitution errors to describe differences between actual and inferred ancestral sequences.

## Supplementary Material

Supplementary Data
