# Supplementary material for: Alignment Modulates Ancestral Sequence Reconstruction Accuracy
Source: Mol Biol Evol. 2018 Apr 3;35(7):1783–97. doi: 10.1093/molbev/msy055 (PMC5995191; doi:10.1093/molbev/msy055)
Supplement: Supplementary Data [file msy055_supp.zip › MBE_Vialle_Revised_Supp_Figures_and_Tables.docx]

SUPPLEMENTARY MATERIAL – FIGURES AND TABLES

Alignment modulates ancestral sequence reconstruction accuracy

**Ricardo Assunção Vialle^1,2,3^, Asif U Tamuri^1,4^ and Nick Goldman^1^**

^1^European Molecular Biology Laboratory, European Bioinformatics Institute,
Wellcome Genome Campus, Hinxton CB10 1SD, UK

^2^Department of Biochemistry and Immunology, Federal University of Minas Gerais, Belo Horizonte, Minas Gerais, Brazil

^3^Laboratory of Human and Medical Genetics, Federal University of Pará, Belém, Pará, Brazil

^4^University College London, London WC1E 6BT, UK

**SUPPLEMENTARY FIGURES**


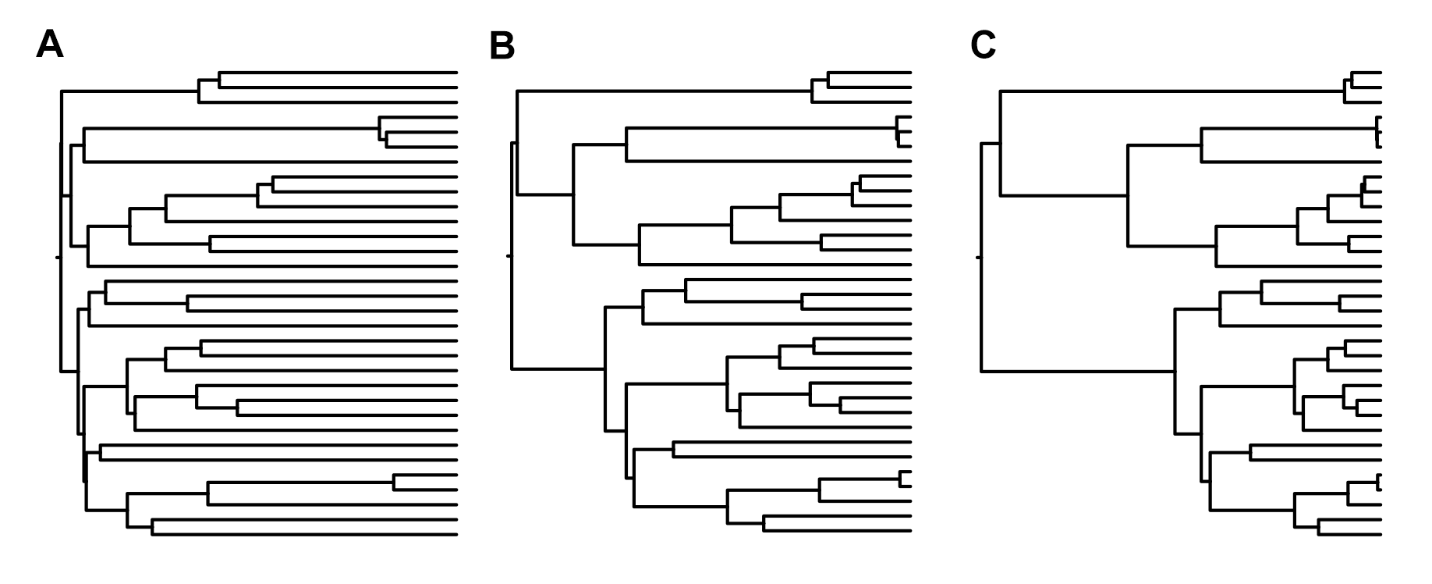


**Supplementary Figure S1 - Sampling fraction effects on tree shape.** Example 32-taxon trees generated from the birth-death process with species sampling using sampling fractions of (A) 0.01, (B) 0.25 and (C) 0.99.


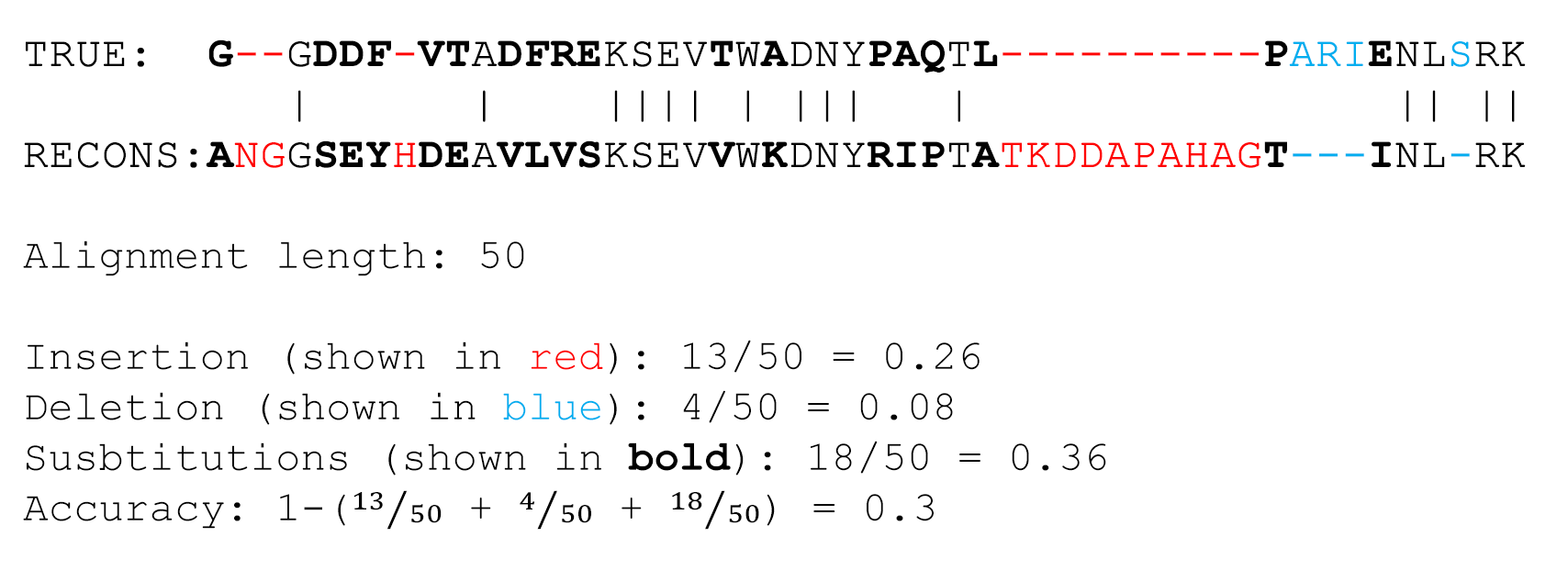


**Supplementary Figure S2 - Reconstruction accuracy calculation.** An example of calculated reconstruction accuracy utilizing alignment between the true sequence at a node and its reconstruction.


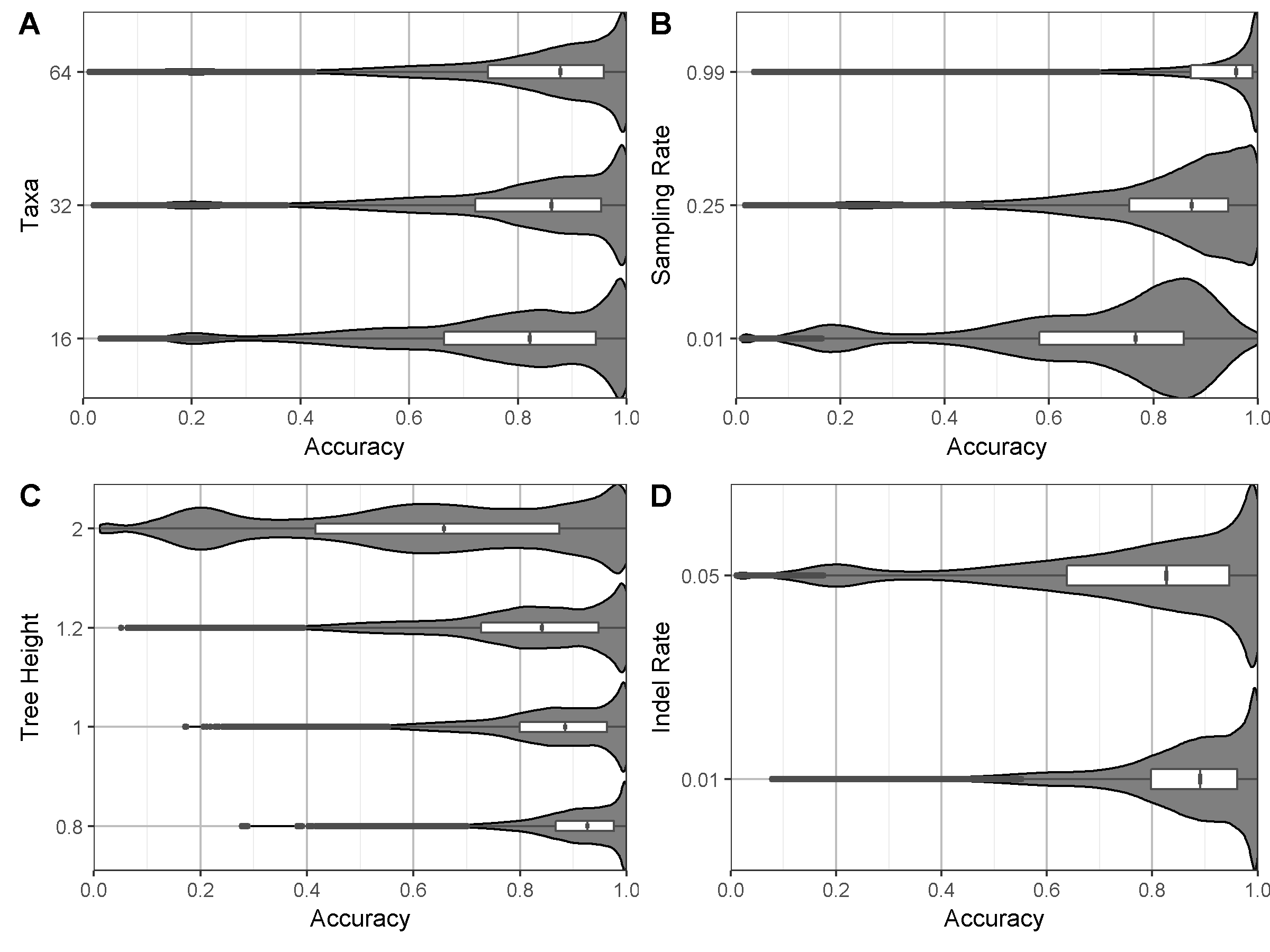


**Supplementary Figure S3 - Overview comparison of parameter effects on reconstruction accuracy.** Accuracy distribution of all reconstructed nodes as a function of one simulation parameter, summarized over all remaining conditions and all aligners. The distributions highlight how each parameter configuration affects the difficulty for ASR in terms of accuracy. Plots show reconstruction accuracy by (A) number of taxa, (B) sampling fraction, (C) tree height and (D) indel rate. Differences are significant for all pairs of accuracy distributions within each panel (pair-wise t-test, Benjamini-Hochberg/False Discovery Rate adjusted *P-*value < 0.01).


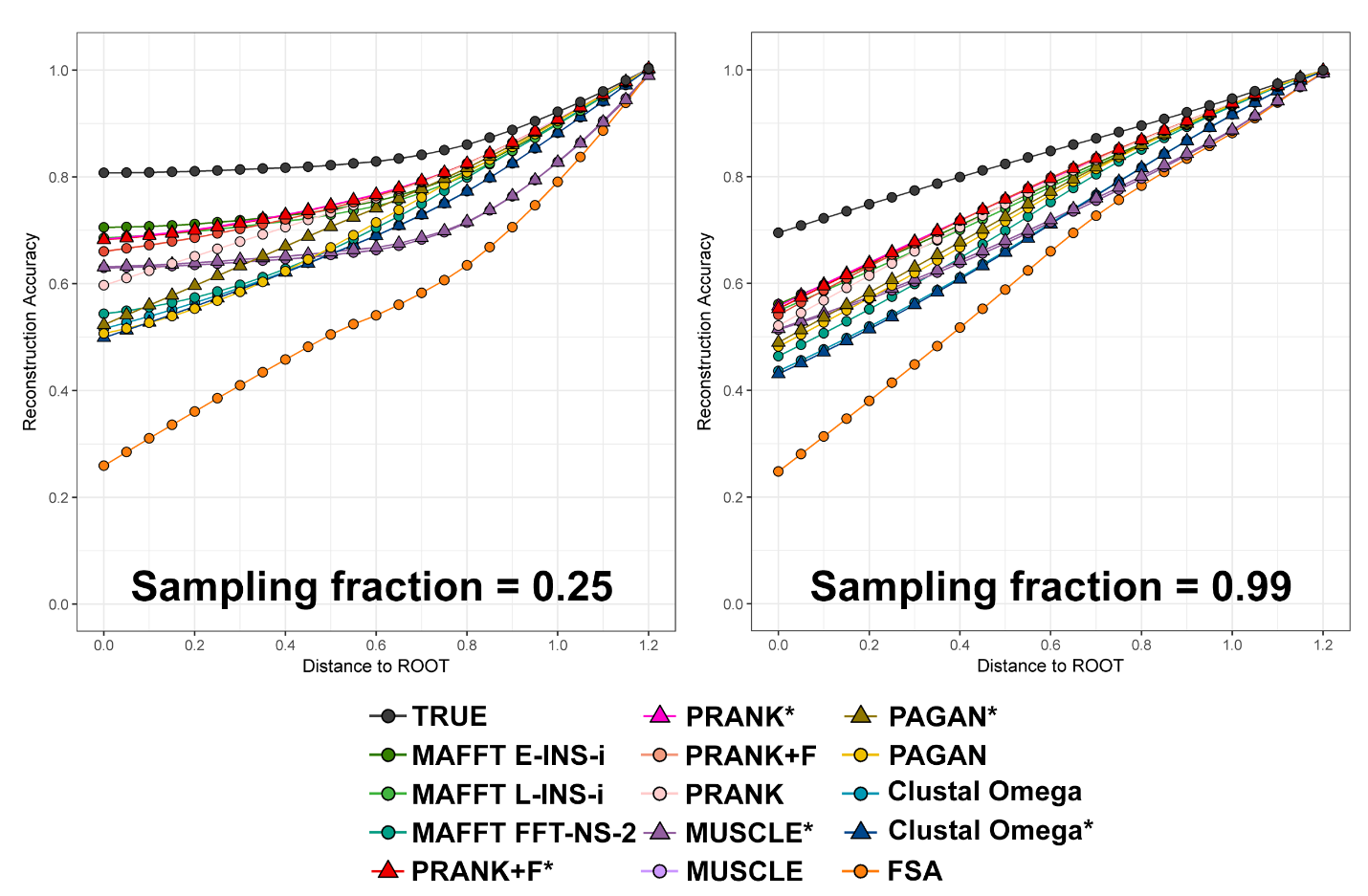


**Supplementary Figure S4 - Reconstruction accuracy by distance to root under sampling fraction of 0.25 and 0.99.**  Reconstruction accuracy at different distances from the root using simulation parameters: 64 taxa, tree height 1.2, indel rate 0.05 and sampling fraction parameter 0.25 (left) and 0.99 (right). Curves are the locally weighted scatterplot smoothing (LOESS) of average reconstruction accuracy by distance to root for each MSA tool.


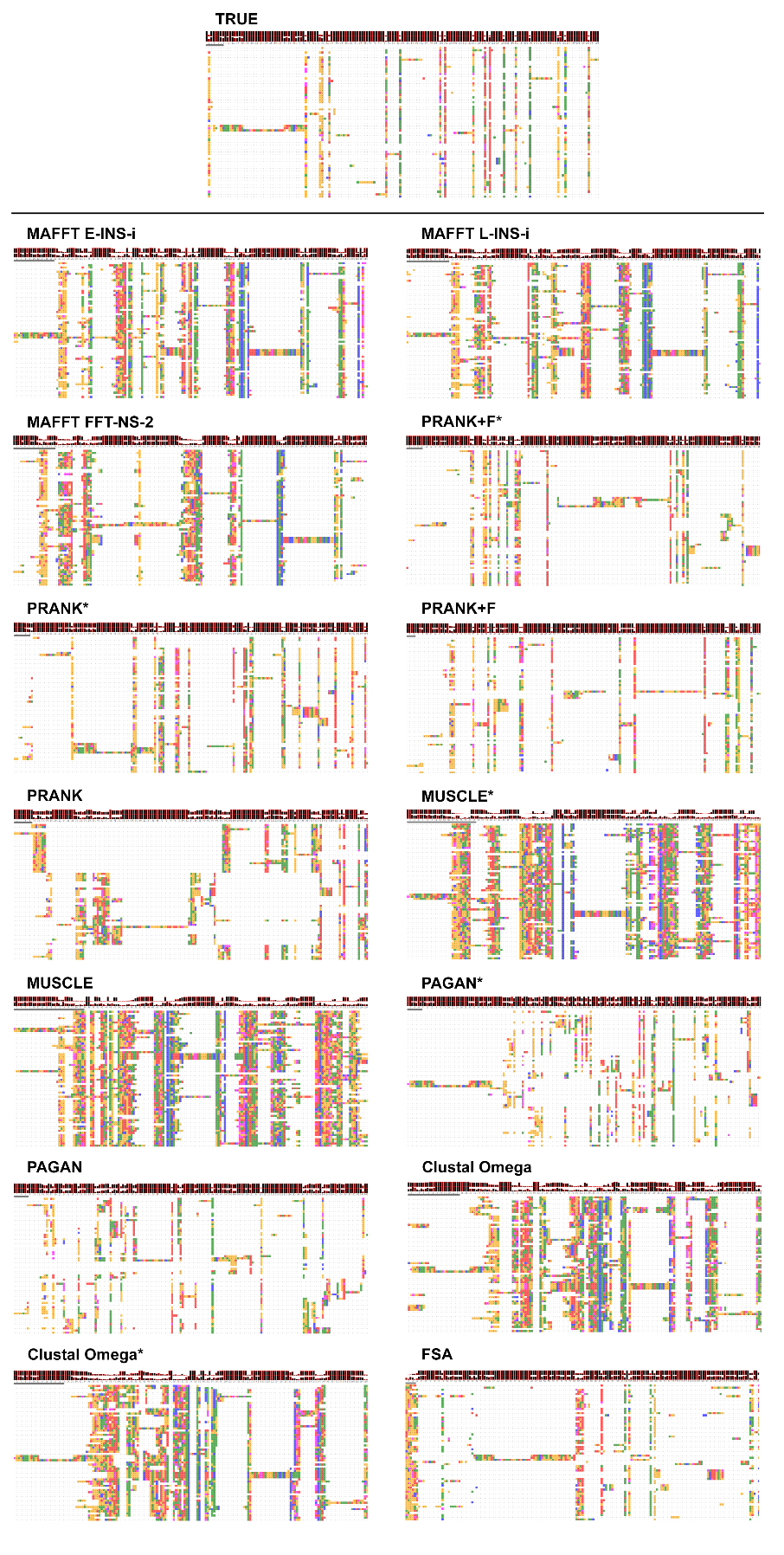


**Supplementary Figure S5 - Alignment overview.** Illustrative partial alignments for each method using sequences simulated with scenario parameters of 64 taxa, tree height 1.2, sampling fraction 0.01 and indel rate 0.05. Alignment visualizations performed using BioJS MSA Viewer (Yachdav et al. 2015).

**SUPPLEMENTARY TABLES**

**Supp. Table S1 - Number of MSA tools with significant differences compared with the baseline for each scenario**

| **Indel Rate = 0.01** | | | | | | | | | | | | |
| --- | --- | --- | --- | --- | --- | --- | --- | --- | --- | --- | --- | --- |
|  | **Tree Height 0.8** | | | **Tree Height 1.0** | | | **Tree Height 1.2** | | | **Tree Height 2.0** | | |
|  | **16 Taxa** | **32 Taxa** | **64 Taxa** | **16 Taxa** | **32 Taxa** | **64 Taxa** | **16 Taxa** | **32 Taxa** | **64 Taxa** | **16 Taxa** | **32 Taxa** | **64 Taxa** |
| **Sampling Fraction 0.01** | 14 | 14 | 14 | 14 | 14 | 14 | 14 | 14 | 14 | 14 | 14 | 14 |
| **Sampling Fraction 0.25** | 0 | 0 | 6 | 0 | 4 | 8 | 0 | 7 | 14 | 14 | 14 | 14 |
| **Sampling Fraction 0.99** | 0 | 0 | 0 | 0 | 0 | 0 | 0 | 0 | 0 | 0 | 0 | 7 |
| **Indel Rate = 0.05** | | | | | | | | | | | | |
|  | **Tree Height 0.8** | | | **Tree Height 1.0** | | | **Tree Height 1.2** | | | **Tree Height 2.0** | | |
|  | **16 Taxa** | **32 Taxa** | **64 Taxa** | **16 Taxa** | **32 Taxa** | **64 Taxa** | **16 Taxa** | **32 Taxa** | **64 Taxa** | **16 Taxa** | **32 Taxa** | **64 Taxa** |
| **Sampling Fraction 0.01** | 14 | 14 | 14 | 14 | 14 | 14 | 14 | 14 | 14 | 14 | 14 | 14 |
| **Sampling Fraction 0.25** | 14 | 14 | 14 | 14 | 14 | 14 | 14 | 14 | 14 | 14 | 14 | 14 |
| **Sampling Fraction 0.99** | 5 | 10 | 14 | 5 | 14 | 14 | 14 | 14 | 14 | 14 | 14 | 14 |
| Note: Table showing the number of aligners (out of 14) with a statistical difference (Mann-Whitney-Wilcoxon test, Benjamin-Hochberg/False Discovery Rate adjusted *P*-value < 0.01) between overall accuracy distribution in comparison with reconstruction using true sequences (baseline). | | | | | | | | | | | | |

**Supp. Table S2 – Reconstruction accuracy variation along the tree**

| **Alignment method** | **Residual Standard Error** |
| --- | --- |
| True | 0.0403 |
| MUSCLE* | 0.0522 |
| Clustal Omega* | 0.0528 |
| MAFFT E-INS-i | 0.0548 |
| MUSCLE | 0.0562 |
| MAFFT L-INS-i | 0.0568 |
| Clustal Omega | 0.0569 |
| PAGAN* | 0.0637 |
| PRANK* | 0.0746 |
| PRANK+F* | 0.0773 |
| MAFFT FFT-NS-2 | 0.0781 |
| PAGAN | 0.0798 |
| PRANK | 0.0894 |
| PRANK+F | 0.0991 |
| FSA | 0.1117 |

Note: Reconstruction accuracy variation is measured in terms of the residual standard error of a Local Polynomial Regression Fitting (LOESS) curve of reconstruction accuracies by distance to root using simulation parameters of 64 taxa, tree height 1.2, sampling fraction 0.01 and indel rate 0.05 (as shown in Figure 4). Values are ordered from the lowest (i.e. low variation) to the highest (i.e. high variation).

**REFERENCES**

Yachdav G, Goldberg T, Wilzbach S, Dao D, Shih I, Choudhary S, Crouch S, Franz M, García A, García LJ, et al. 2015. Anatomy of BioJS, an open source community for the life sciences. Elife 4:e07009.
