# Supplementary material for: Alignment Modulates Ancestral Sequence Reconstruction Accuracy
Source: Mol Biol Evol. 2018 Apr 3;35(7):1783–97. doi: 10.1093/molbev/msy055 (PMC5995191; doi:10.1093/molbev/msy055)
Supplement: Supplementary Data [file msy055_supp.zip › MBE_Vialle_Suppl_Mat_Additional_File_1_reduced.pdf]

Taxa=16 Tree_height=0.8 Sampling_fraction=0.01 Indel_rate=0.01 Indel_model=POW 1.7 20

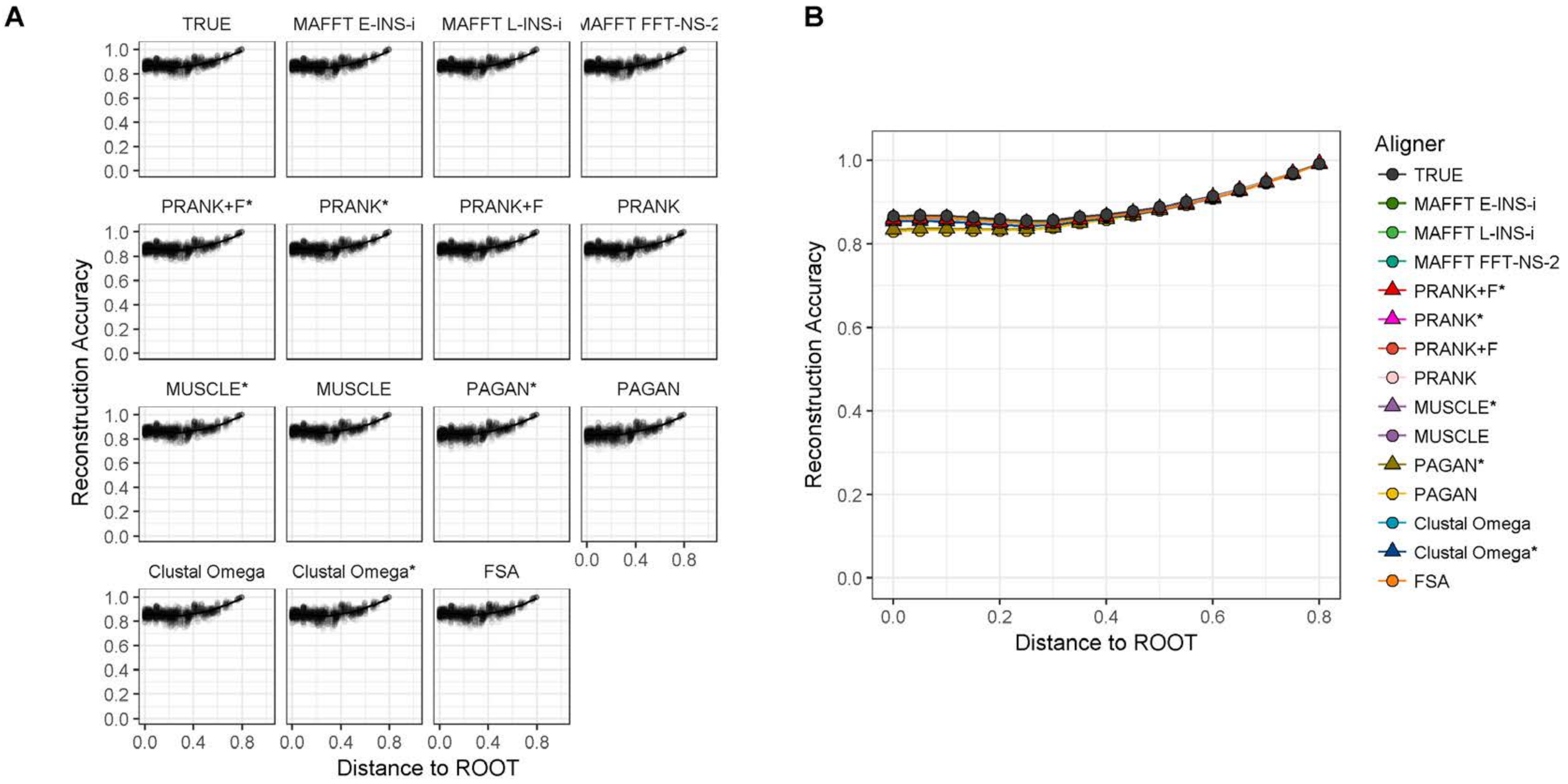

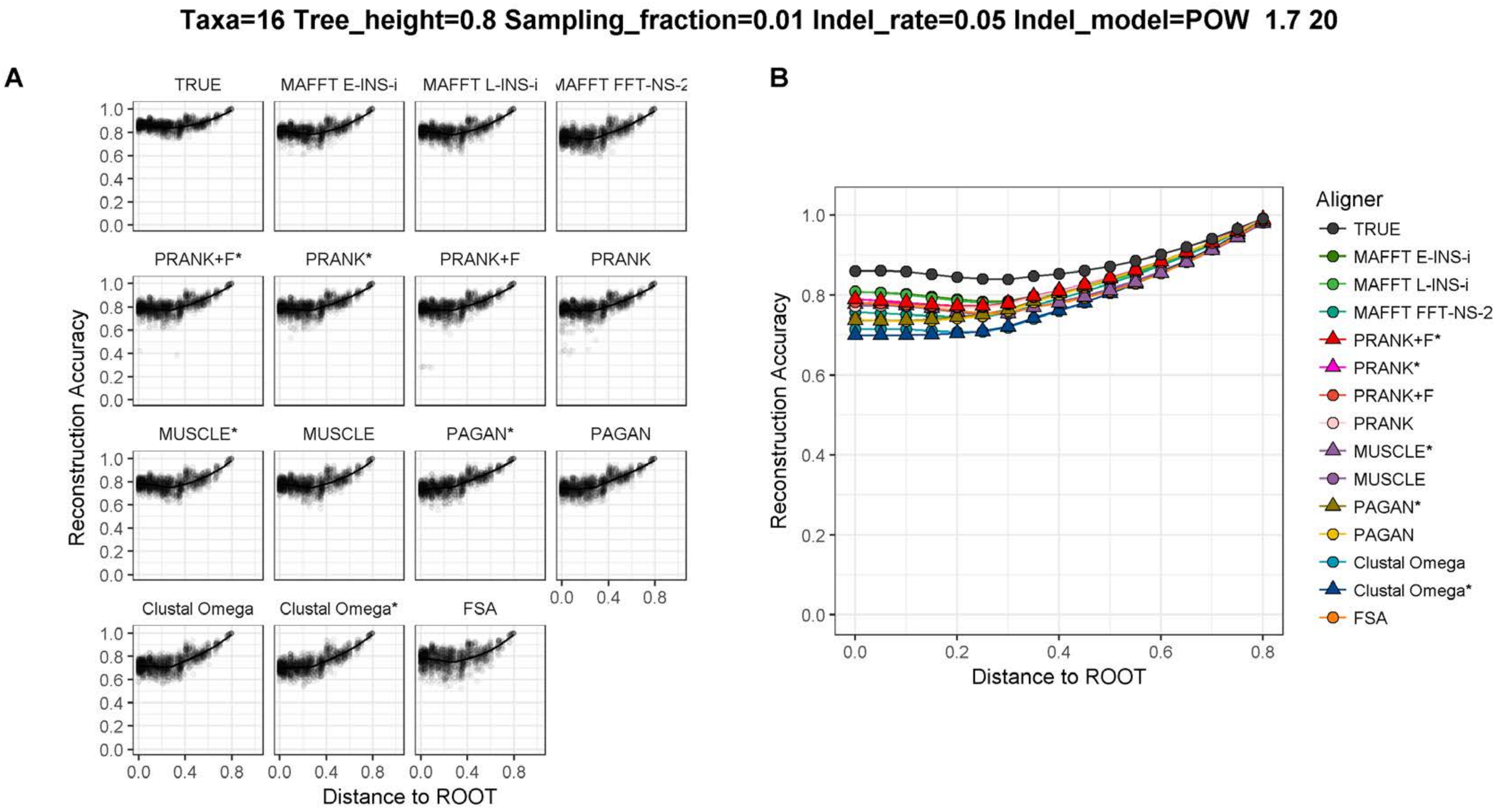
Taxa=16 Tree_height=0.8 Sampling_fraction=0.01 Indel_rate=0.05 Indel_model=POW 1.7 20
A
TRUE
MAFFT E-INS-i
MAFFT L-INS-i
MAFFT FFT-NS-2
PRANK+F*
PRANK*
PRANK+F
PRANK
MUSCLE*
MUSCLE
PAGAN*
PAGAN
Clustal Omega
Clustal Omega*
FSA
Reconstruction Accuracy
Distance to ROOT
B
Reconstruction Accuracy
Distance to ROOT
Aligner
TRUE
MAFFT E-INS-i
MAFFT L-INS-i
MAFFT FFT-NS-2
PRANK+F*
PRANK*
PRANK+F
PRANK
MUSCLE*
MUSCLE
PAGAN*
PAGAN
Clustal Omega
Clustal Omega*
FSA

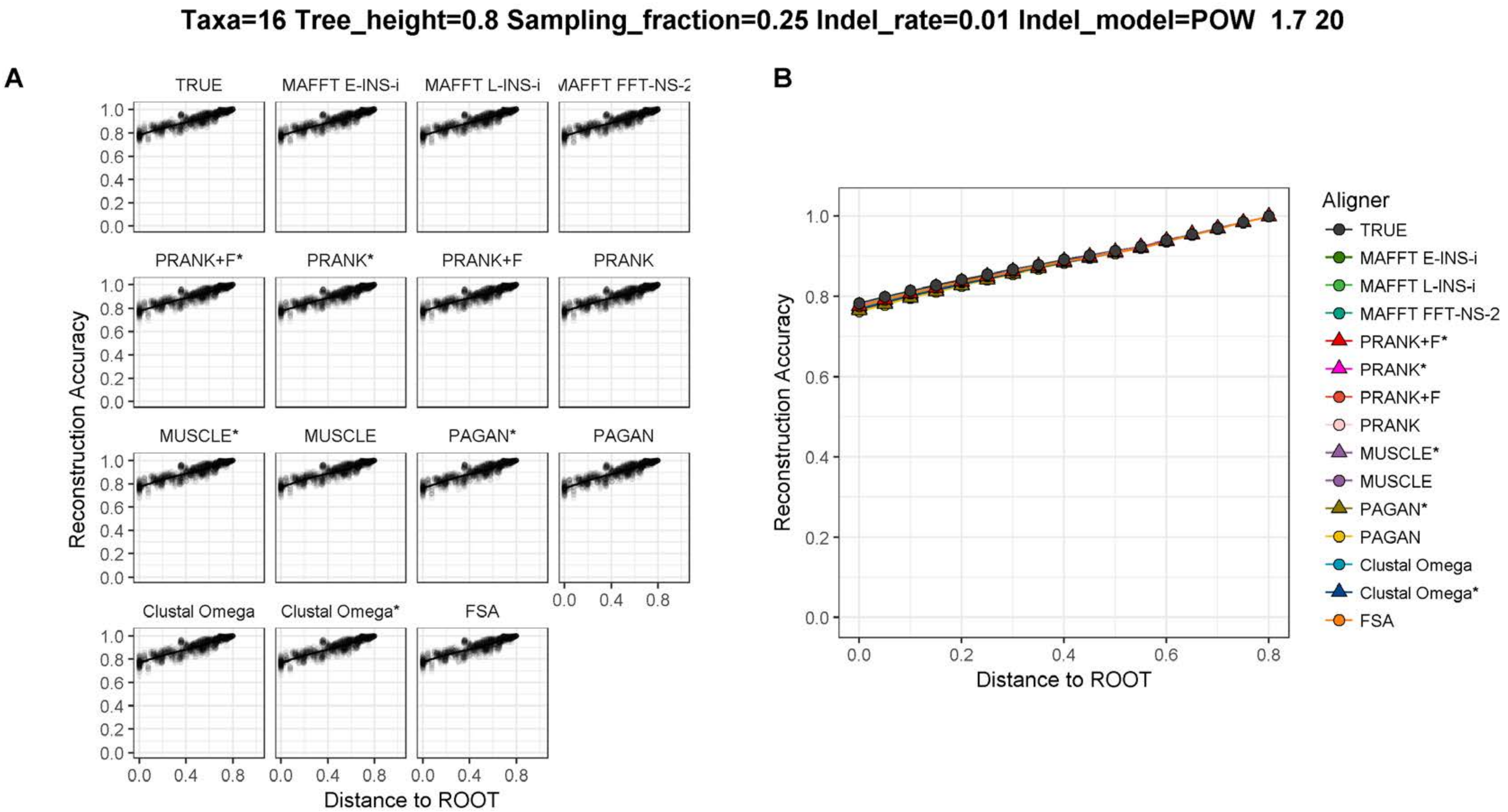

Taxa=16 Tree_height=0.8 Sampling_fraction=0.25 Indel_rate=0.01 Indel_model=POW 1.7 20
A
TRUE
MAFFT E-INS-i
MAFFT L-INS-i
MAFFT FFT-NS-2
PRANK+F*
PRANK*
PRANK+F
PRANK
MUSCLE*
MUSCLE
PAGAN*
PAGAN
Clustal Omega
Clustal Omega*
FSA
Reconstruction Accuracy
Distance to ROOT
B
Reconstruction Accuracy
Distance to ROOT
Aligner
TRUE
MAFFT E-INS-i
MAFFT L-INS-i
MAFFT FFT-NS-2
PRANK+F*
PRANK*
PRANK+F
PRANK
MUSCLE*
MUSCLE
PAGAN*
PAGAN
Clustal Omega
Clustal Omega*
FSA

Taxa=16 Tree_height=0.8 Sampling_fraction=0.25 Indel_rate=0.05 Indel_model=POW 1.7 20

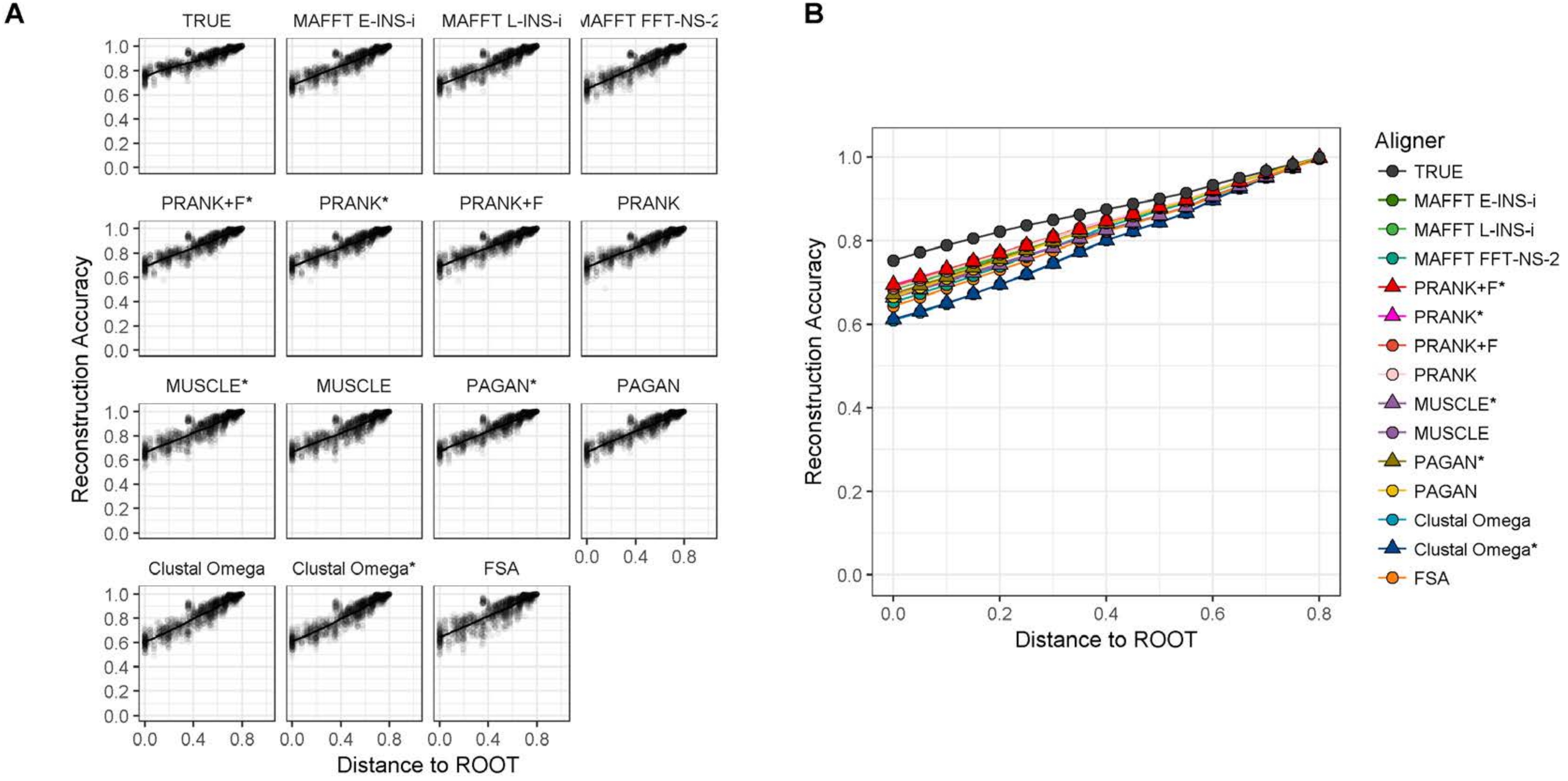

Taxa=16 Tree_height=0.8 Sampling_fraction=0.99 Indel_rate=0.01 Indel_model=POW 1.7 20

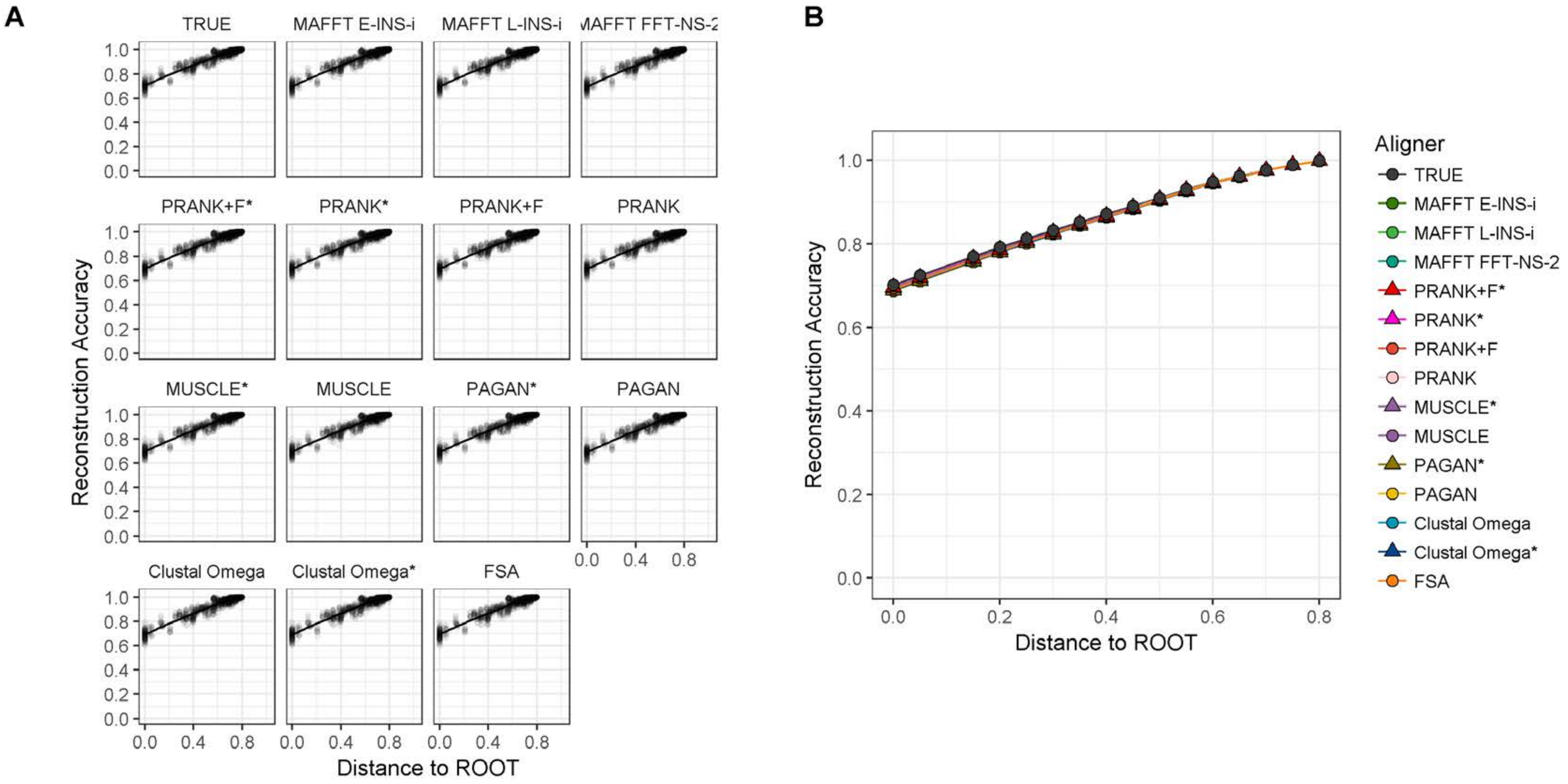

Taxa=16 Tree_height=0.8 Sampling_fraction=0.99 Indel_rate=0.05 Indel_model=POW 1.7 20

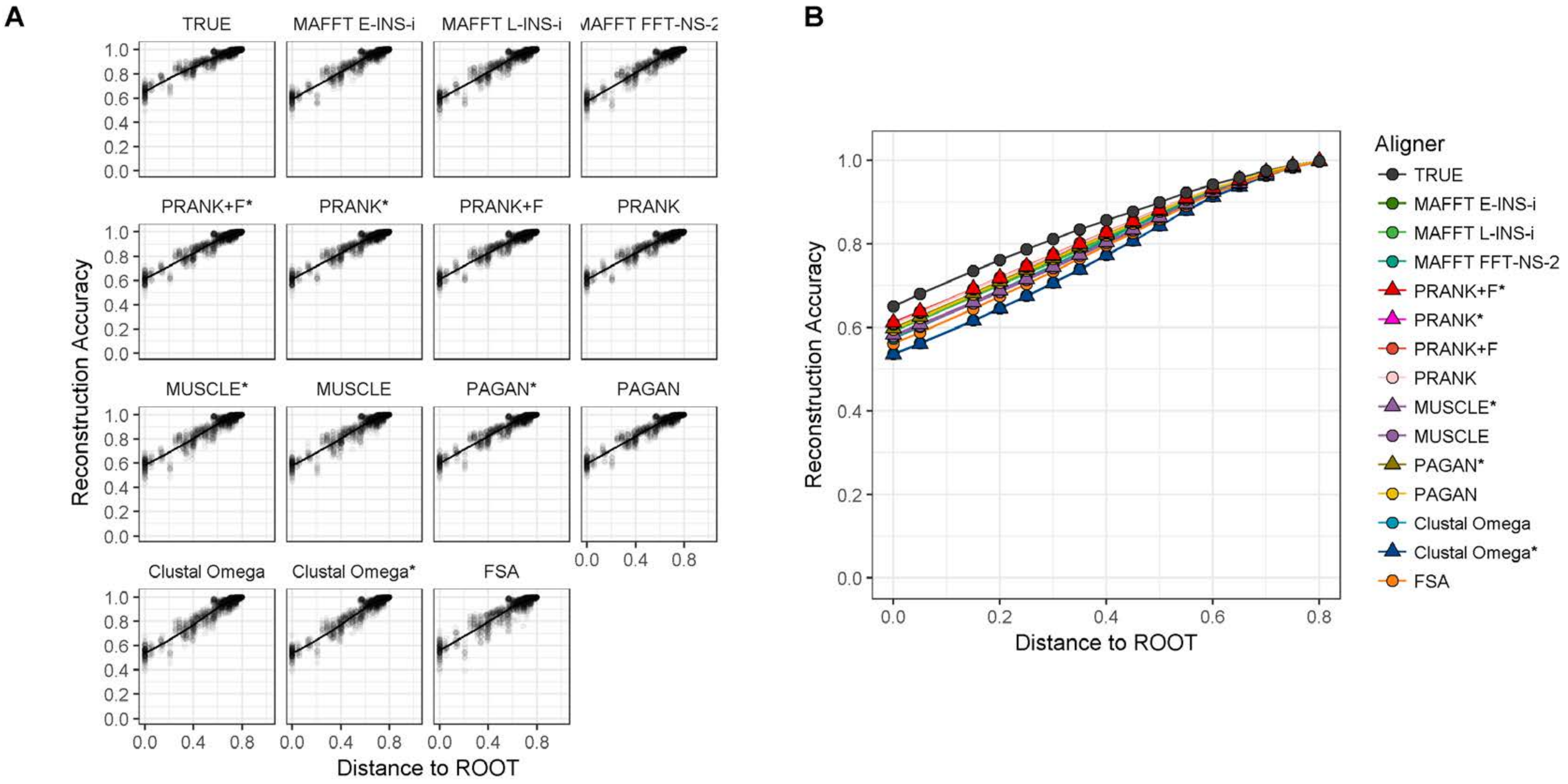

Taxa=32 Tree_height=0.8 Sampling_fraction=0.01 Indel_rate=0.01 Indel_model=POW 1.7 20

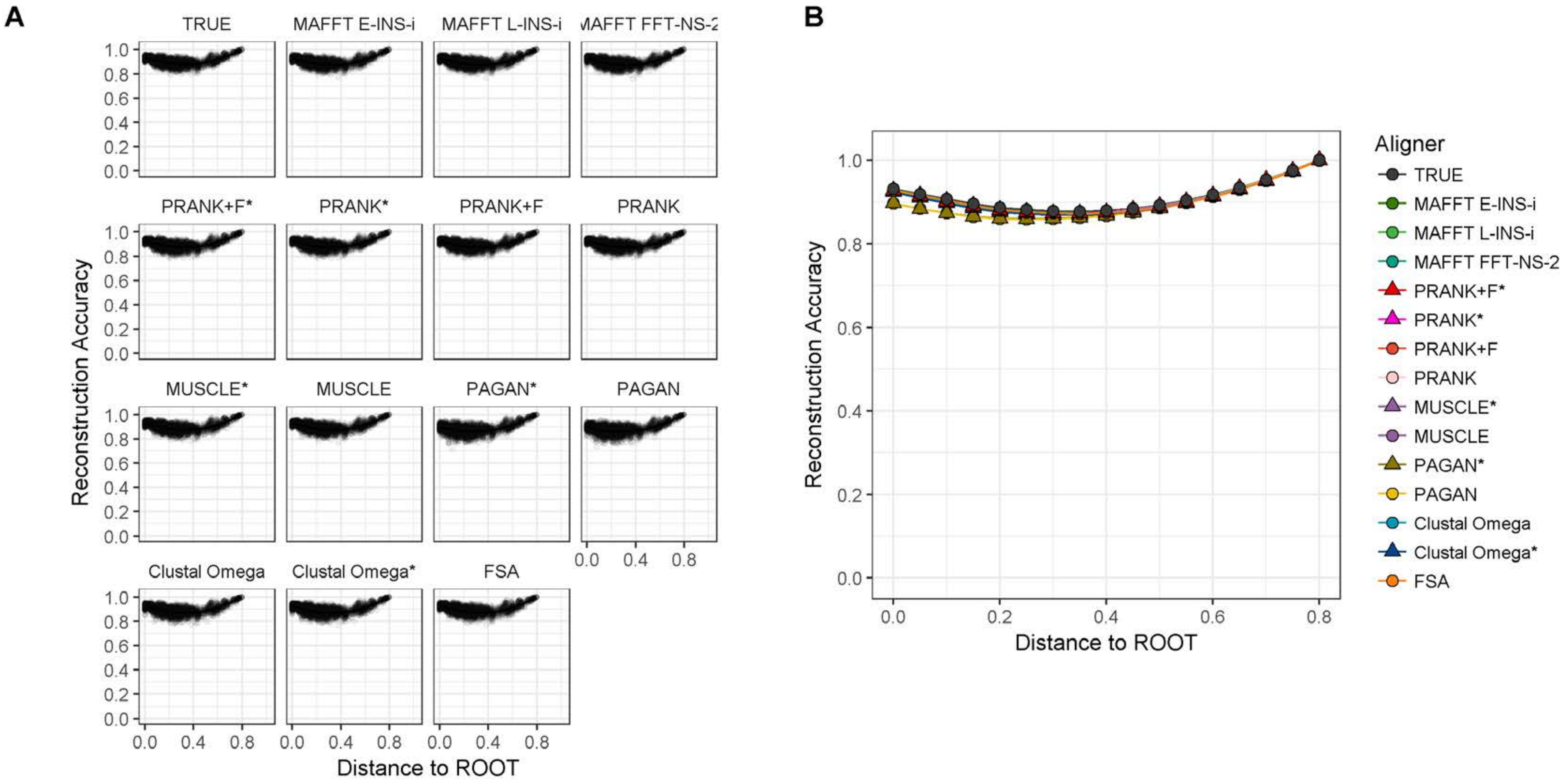

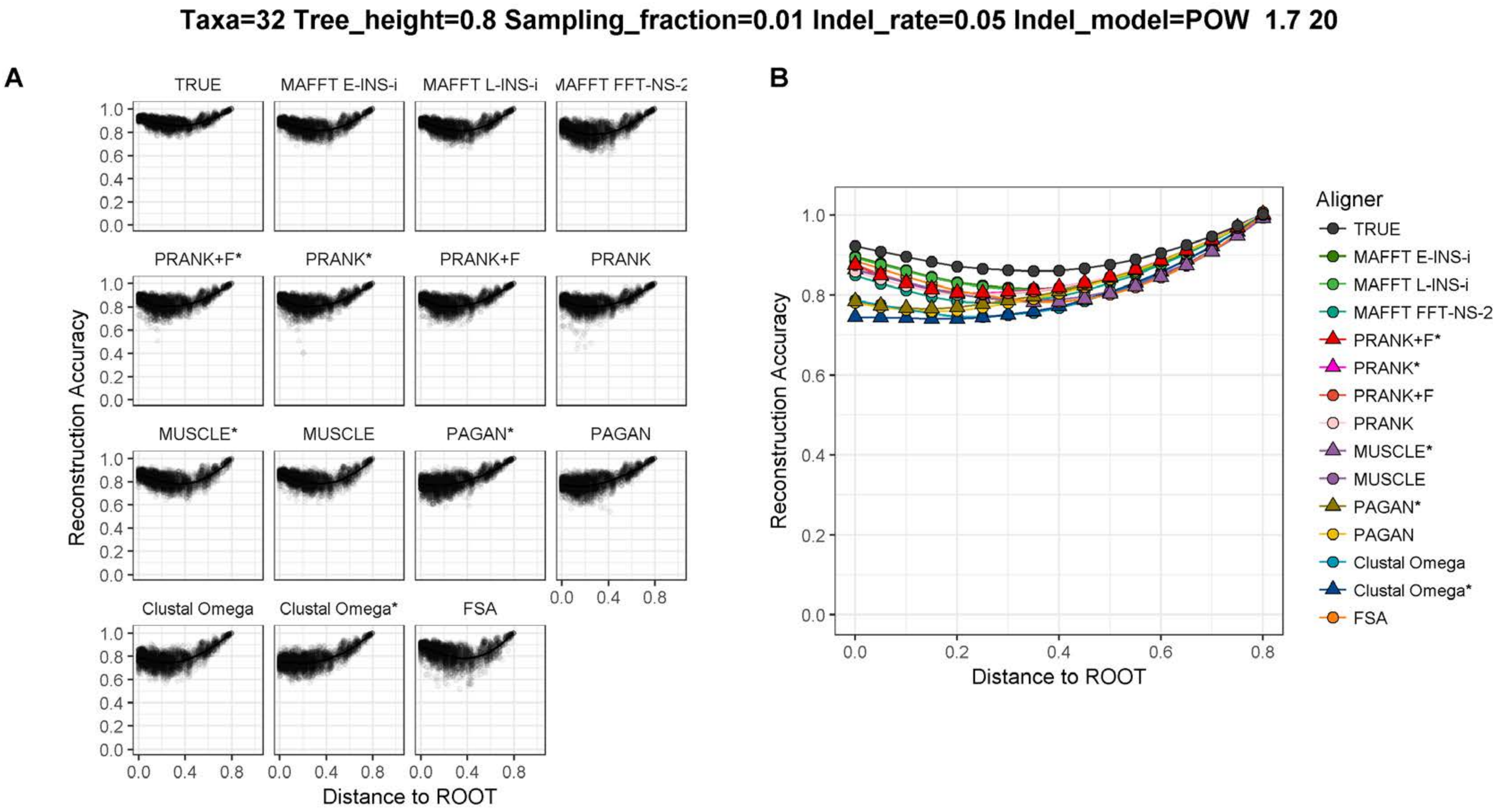

Taxa=32 Tree_height=0.8 Sampling_fraction=0.01 Indel_rate=0.05 Indel_model=POW 1.7 20
A
TRUE
MAFFT E-INS-i
MAFFT L-INS-i
MAFFT FFT-NS-2
PRANK+F*
PRANK*
PRANK+F
PRANK
MUSCLE*
MUSCLE
PAGAN*
PAGAN
Clustal Omega
Clustal Omega*
FSA
Reconstruction Accuracy
Distance to ROOT
B
Reconstruction Accuracy
Distance to ROOT
Aligner
TRUE
MAFFT E-INS-i
MAFFT L-INS-i
MAFFT FFT-NS-2
PRANK+F*
PRANK*
PRANK+F
PRANK
MUSCLE*
MUSCLE
PAGAN*
PAGAN
Clustal Omega
Clustal Omega*
FSA

Taxa=32 Tree_height=0.8 Sampling_fraction=0.25 Indel_rate=0.01 Indel_model=POW 1.7 20

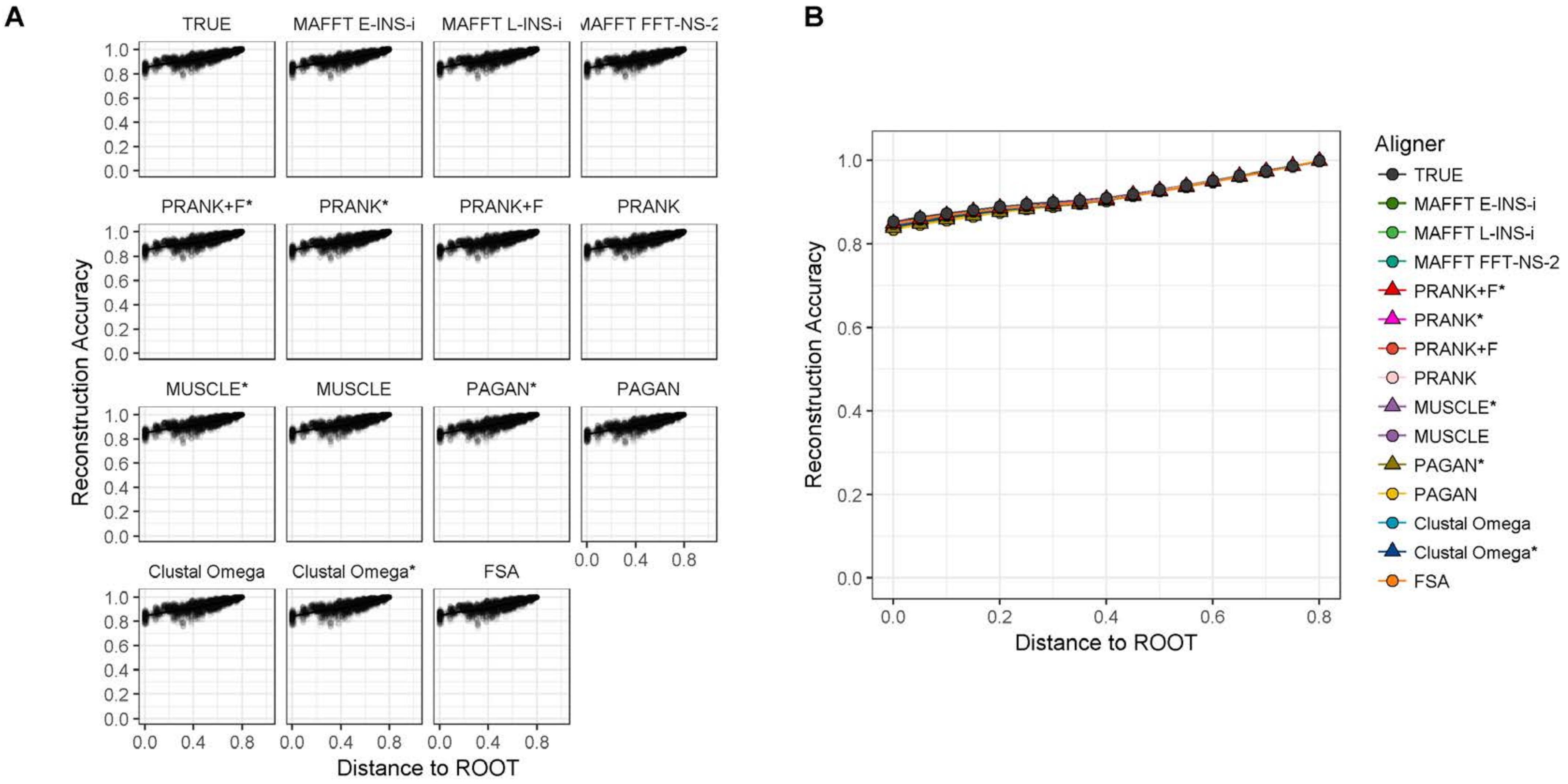

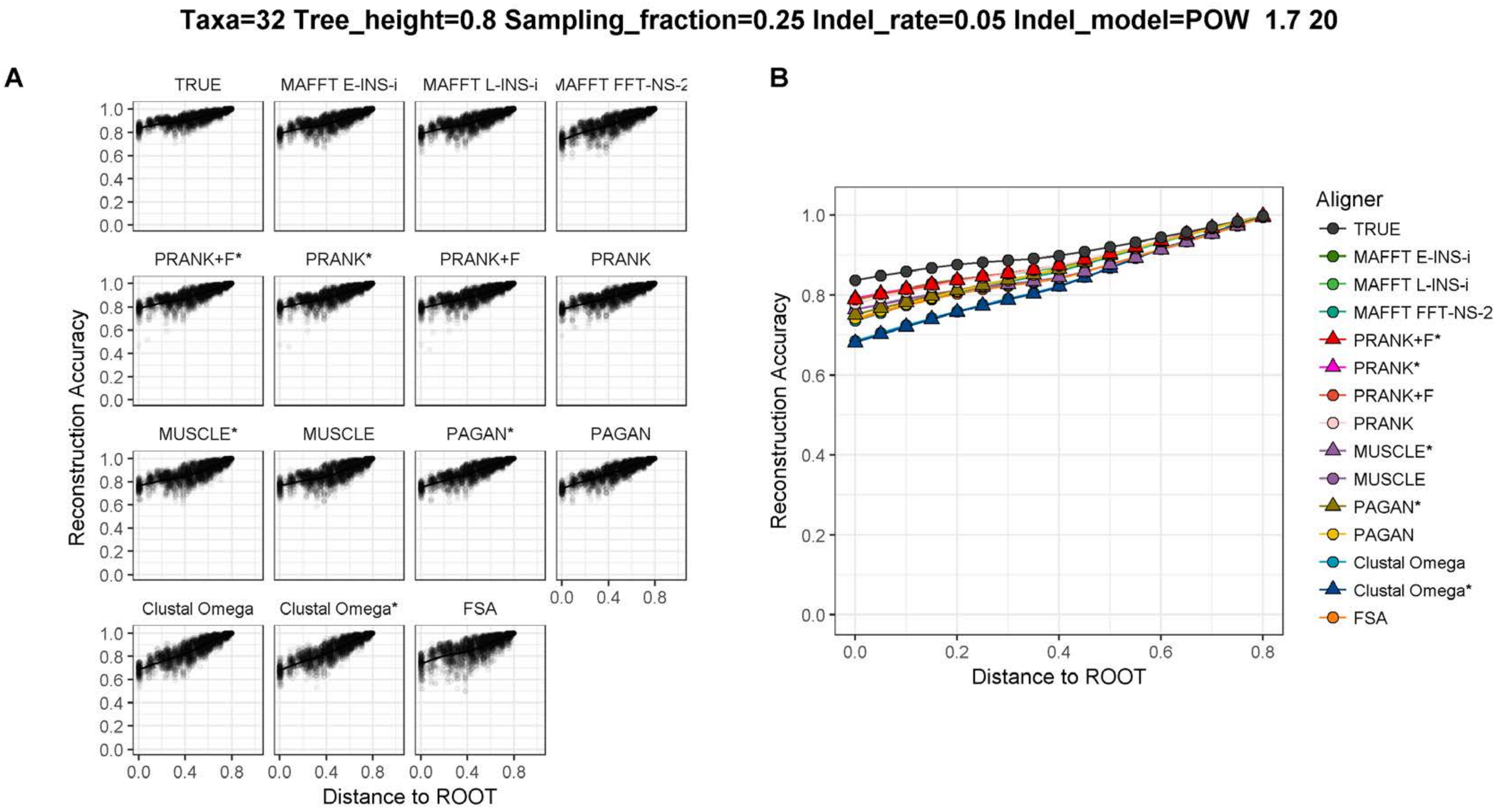

Taxa=32 Tree_height=0.8 Sampling_fraction=0.25 Indel_rate=0.05 Indel_model=POW 1.7 20
A
B
TRUE
MAFFT E-INS-i
MAFFT L-INS-i
MAFFT FFT-NS-2
PRANK+F*
PRANK*
PRANK+F
PRANK
MUSCLE*
MUSCLE
PAGAN*
PAGAN
Clustal Omega
Clustal Omega*
FSA
Reconstruction Accuracy
Distance to ROOT
Aligner

Taxa=32 Tree_height=0.8 Sampling_fraction=0.99 Indel_rate=0.01 Indel_model=POW 1.7 20

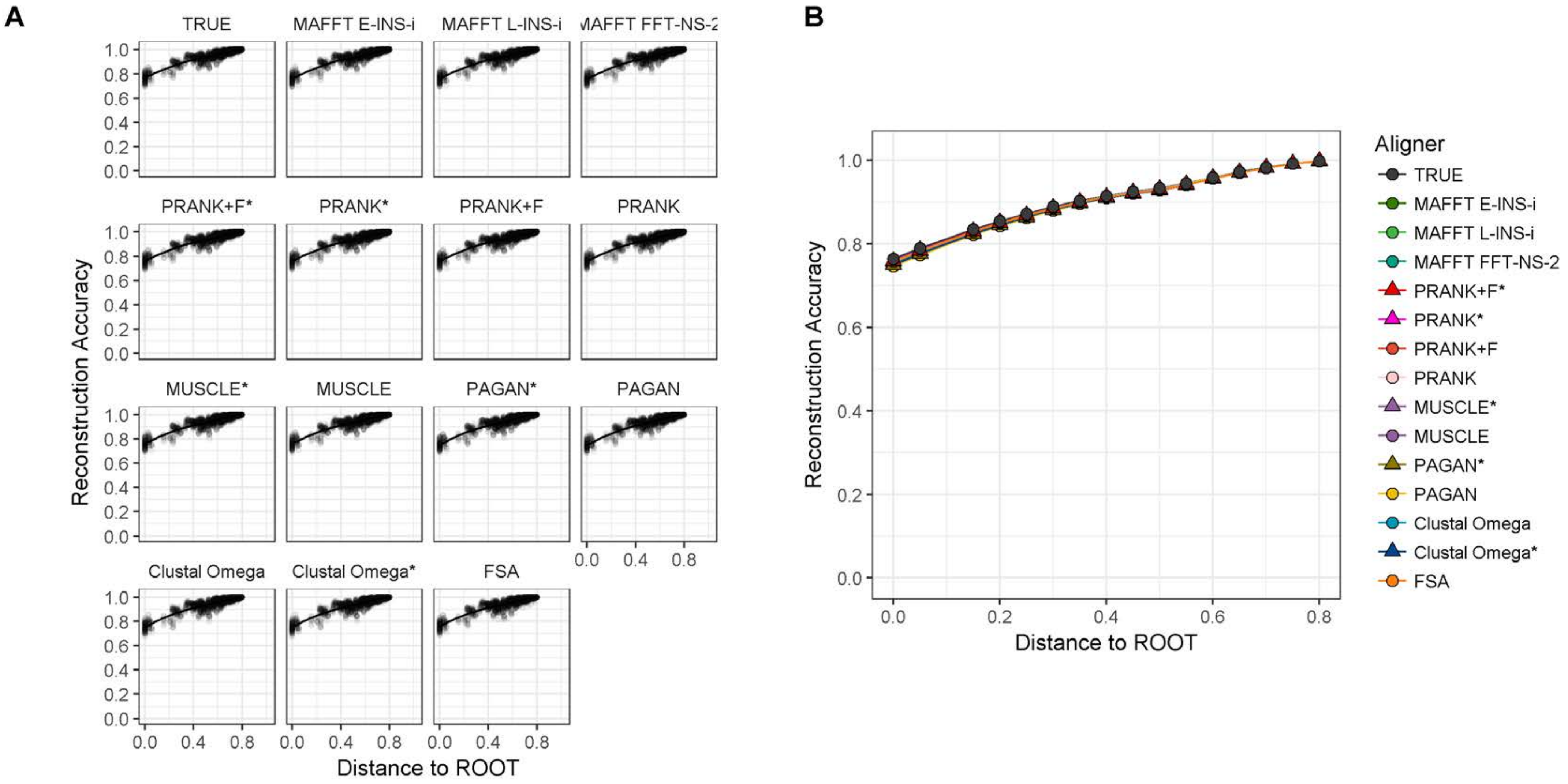

Taxa=32 Tree_height=0.8 Sampling_fraction=0.99 Indel_rate=0.05 Indel_model=POW 1.7 20

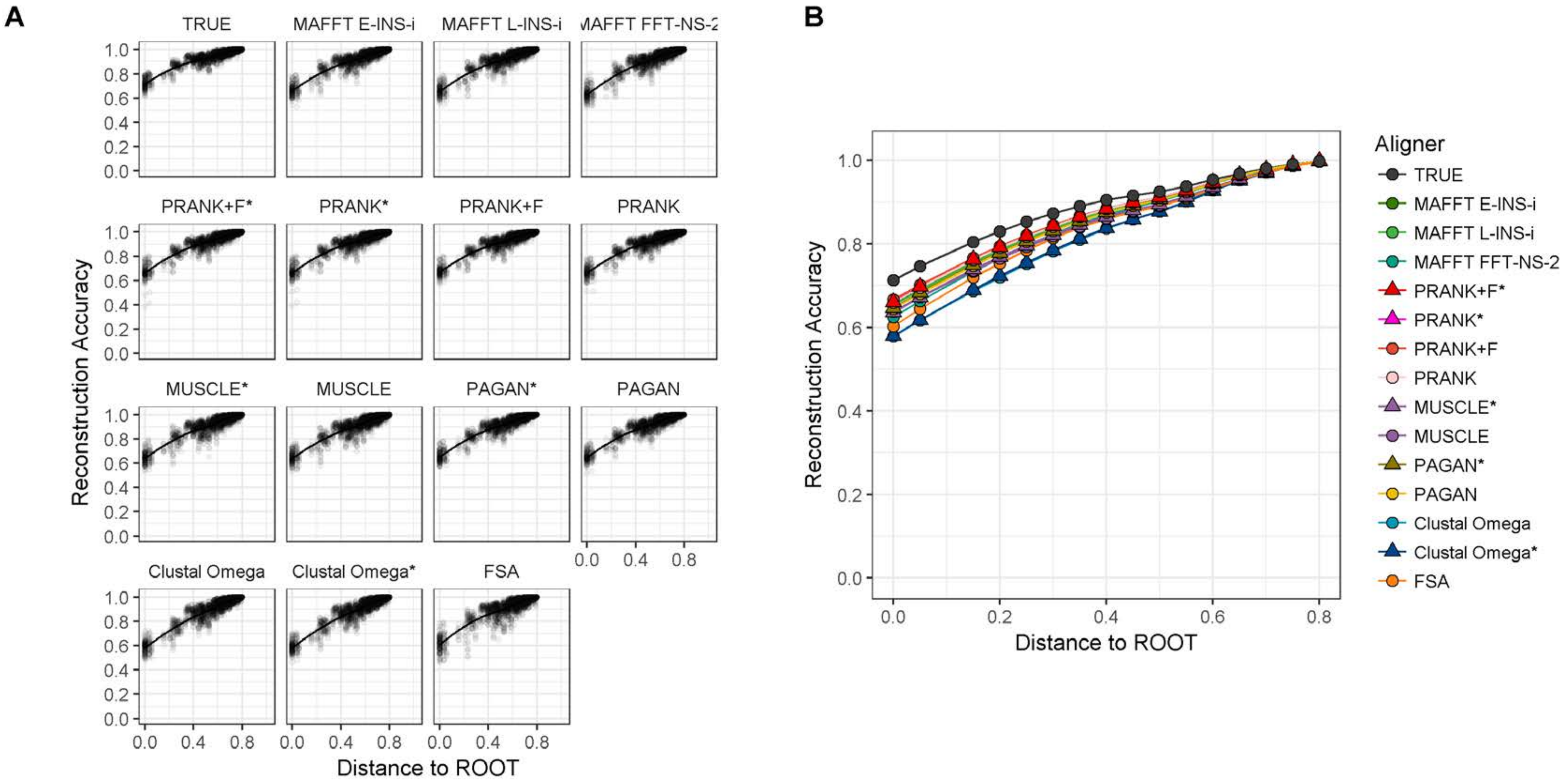

Taxa=64 Tree_height=0.8 Sampling_fraction=0.01 Indel_rate=0.01 Indel_model=POW 1.7 20

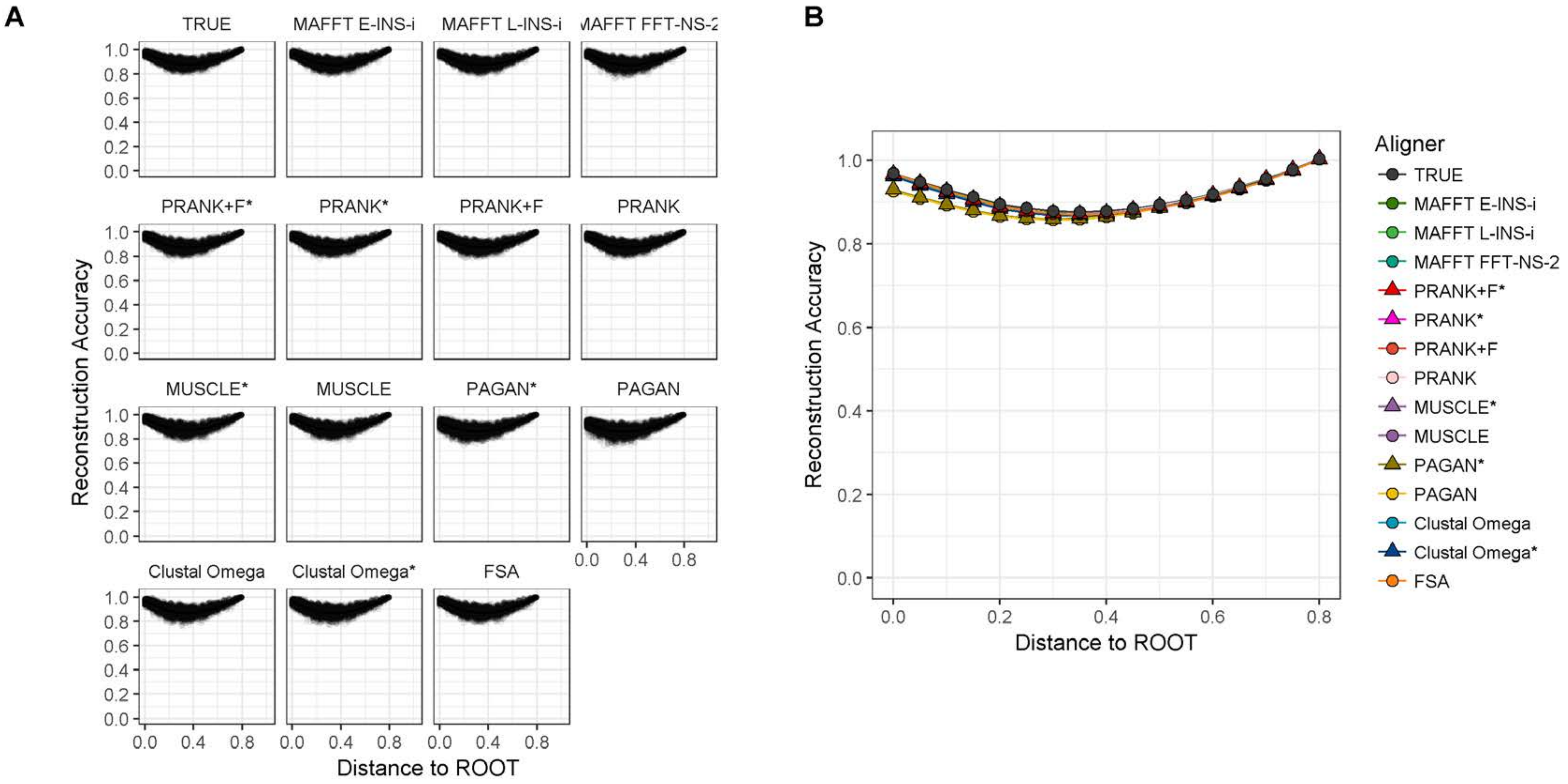

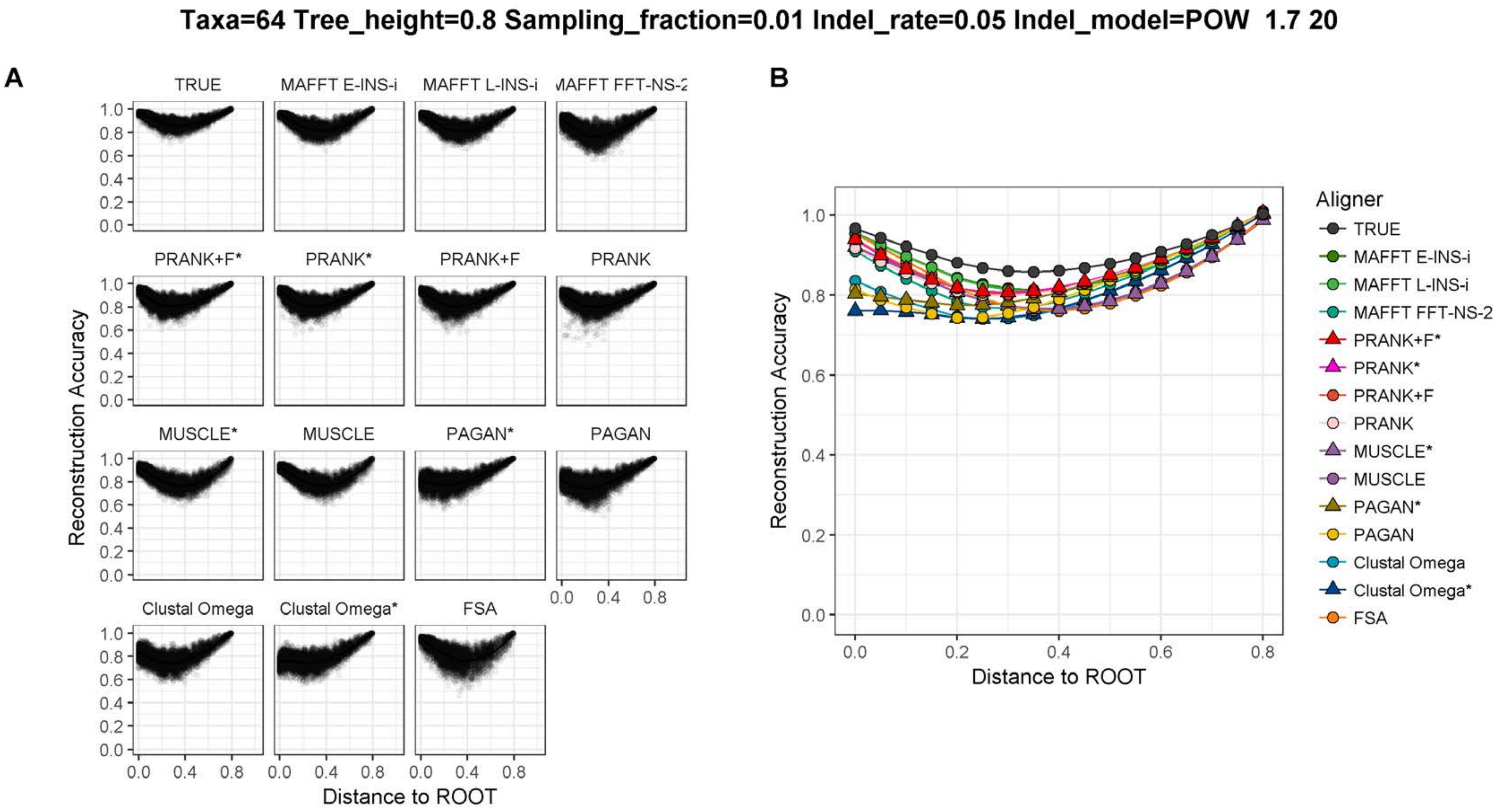
Taxa=64 Tree_height=0.8 Sampling_fraction=0.01 Indel_rate=0.05 Indel_model=POW 1.7 20
A
TRUE
MAFFT E-INS-i
MAFFT L-INS-i
PRANK+F*
PRANK*
PRANK+F
PRANK
MUSCLE*
MUSCLE
PAGAN*
PAGAN
Clustal Omega
Clustal Omega*
FSA
Reconstruction Accuracy
Distance to ROOT
B
Aligner
TRUE
MAFFT E-INS-i
MAFFT L-INS-i
MAFFT FFT-NS-2
PRANK+F*
PRANK*
PRANK+F
PRANK
MUSCLE*
MUSCLE
PAGAN*
PAGAN
Clustal Omega
Clustal Omega*
FSA
Reconstruction Accuracy
Distance to ROOT

Taxa=64 Tree_height=0.8 Sampling_fraction=0.25 Indel_rate=0.01 Indel_model=POW 1.7 20

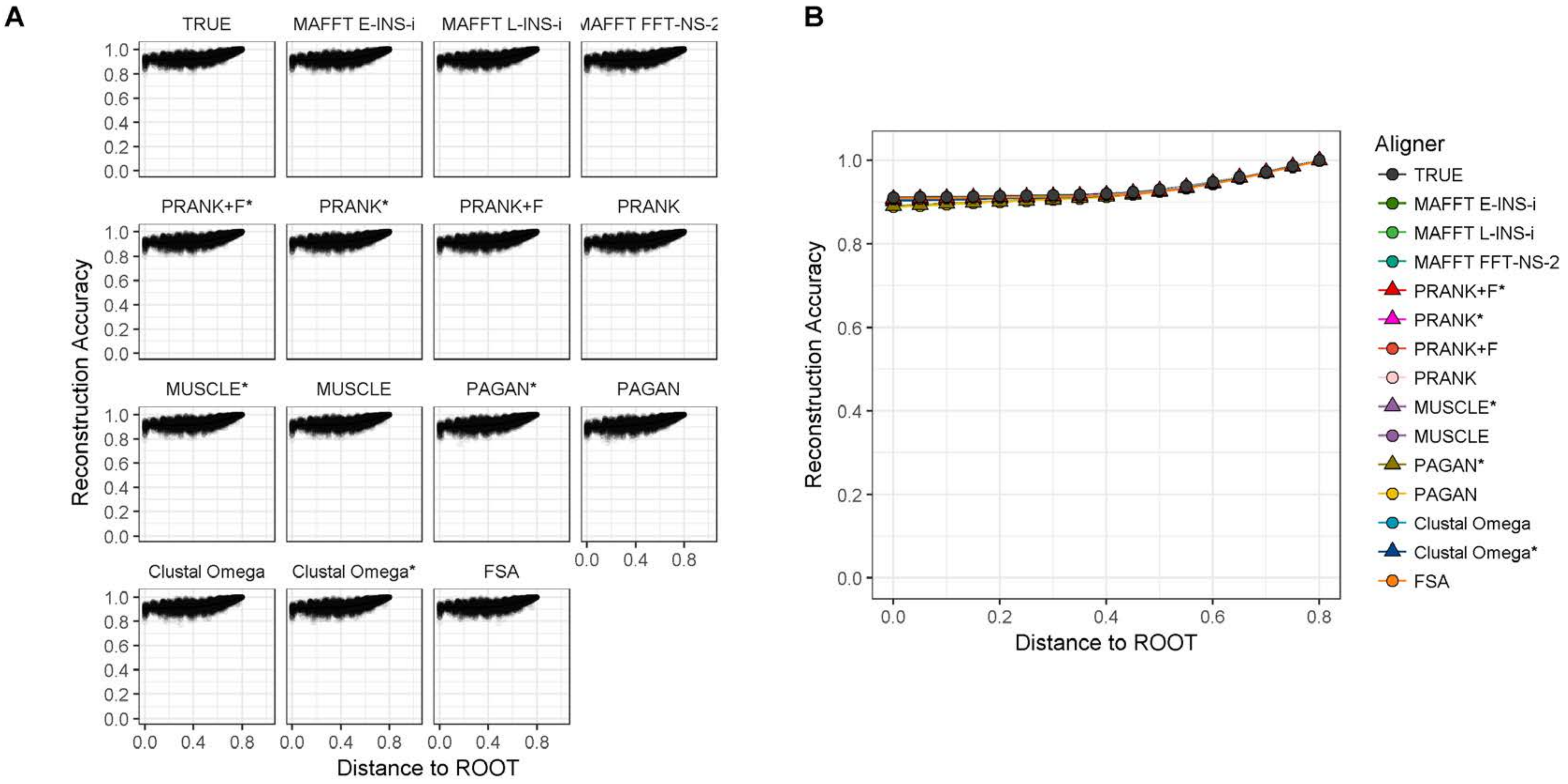

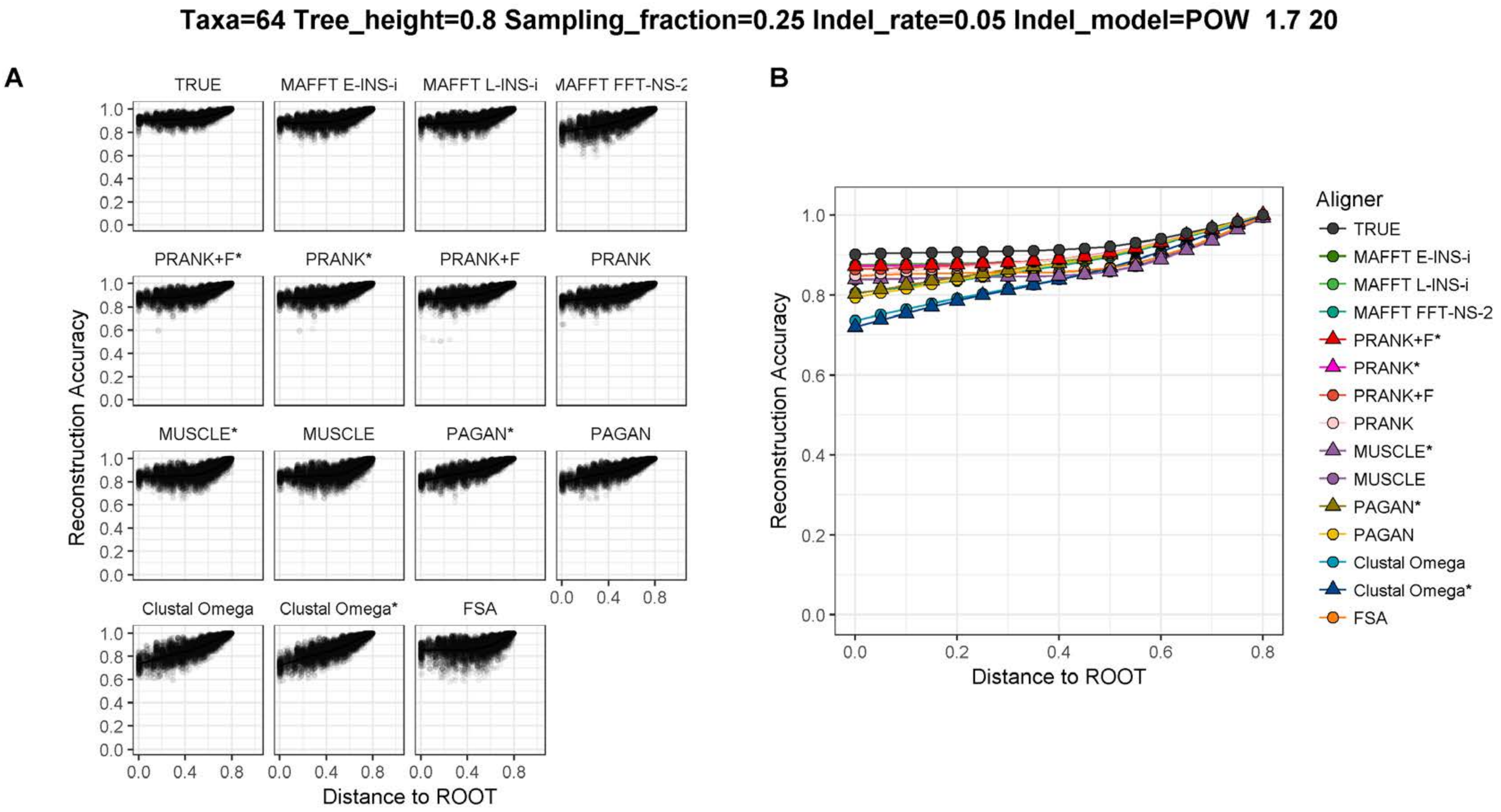

Taxa=64 Tree_height=0.8 Sampling_fraction=0.25 Indel_rate=0.05 Indel_model=POW 1.7 20
A
B
TRUE
MAFFT E-INS-i
MAFFT L-INS-i
MAFFT FFT-NS-2
PRANK+F*
PRANK*
PRANK+F
PRANK
MUSCLE*
MUSCLE
PAGAN*
PAGAN
Clustal Omega
Clustal Omega*
FSA
Reconstruction Accuracy
Distance to ROOT
Aligner

Taxa=64 Tree_height=0.8 Sampling_fraction=0.99 Indel_rate=0.01 Indel_model=POW 1.7 20

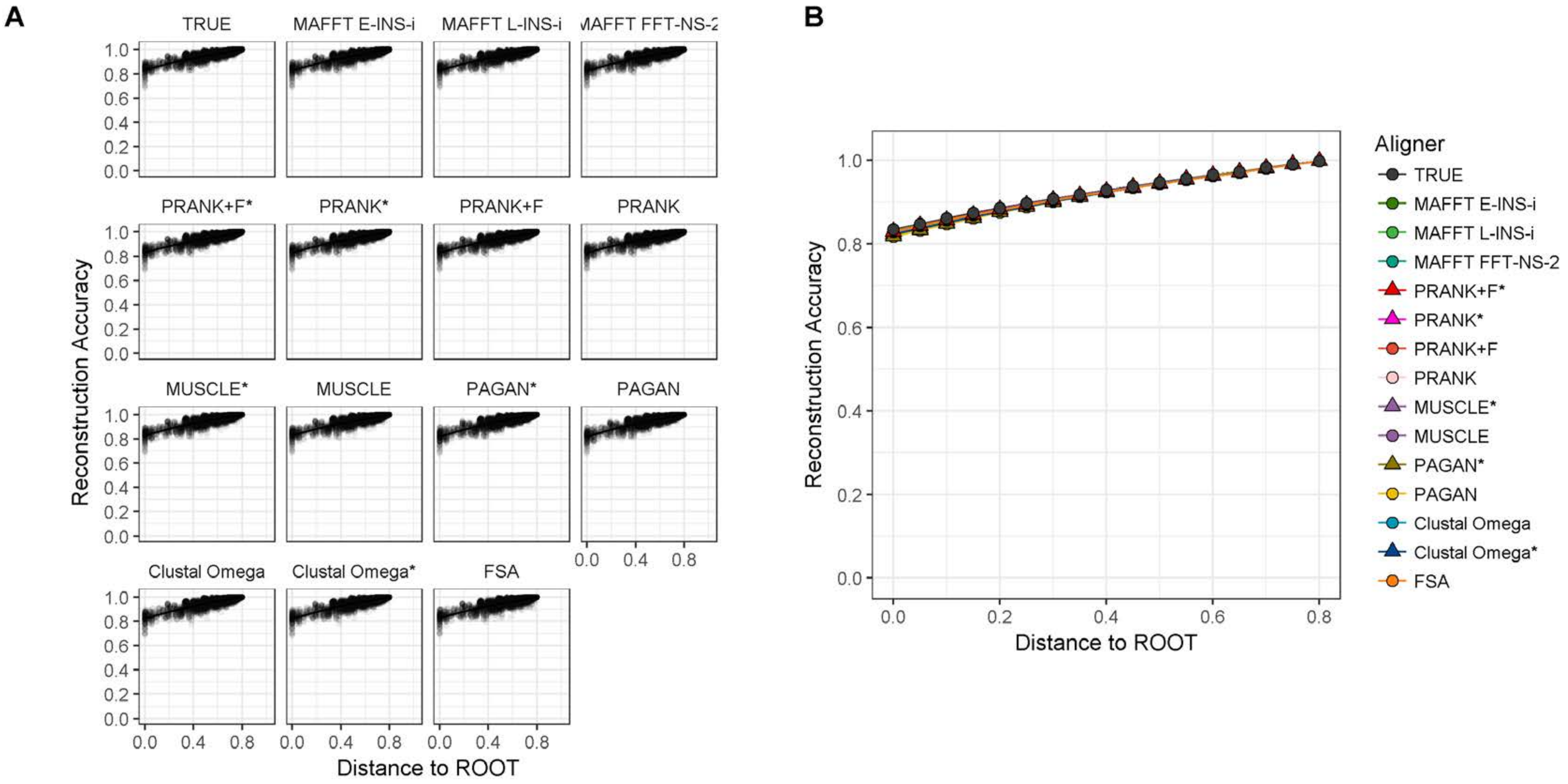

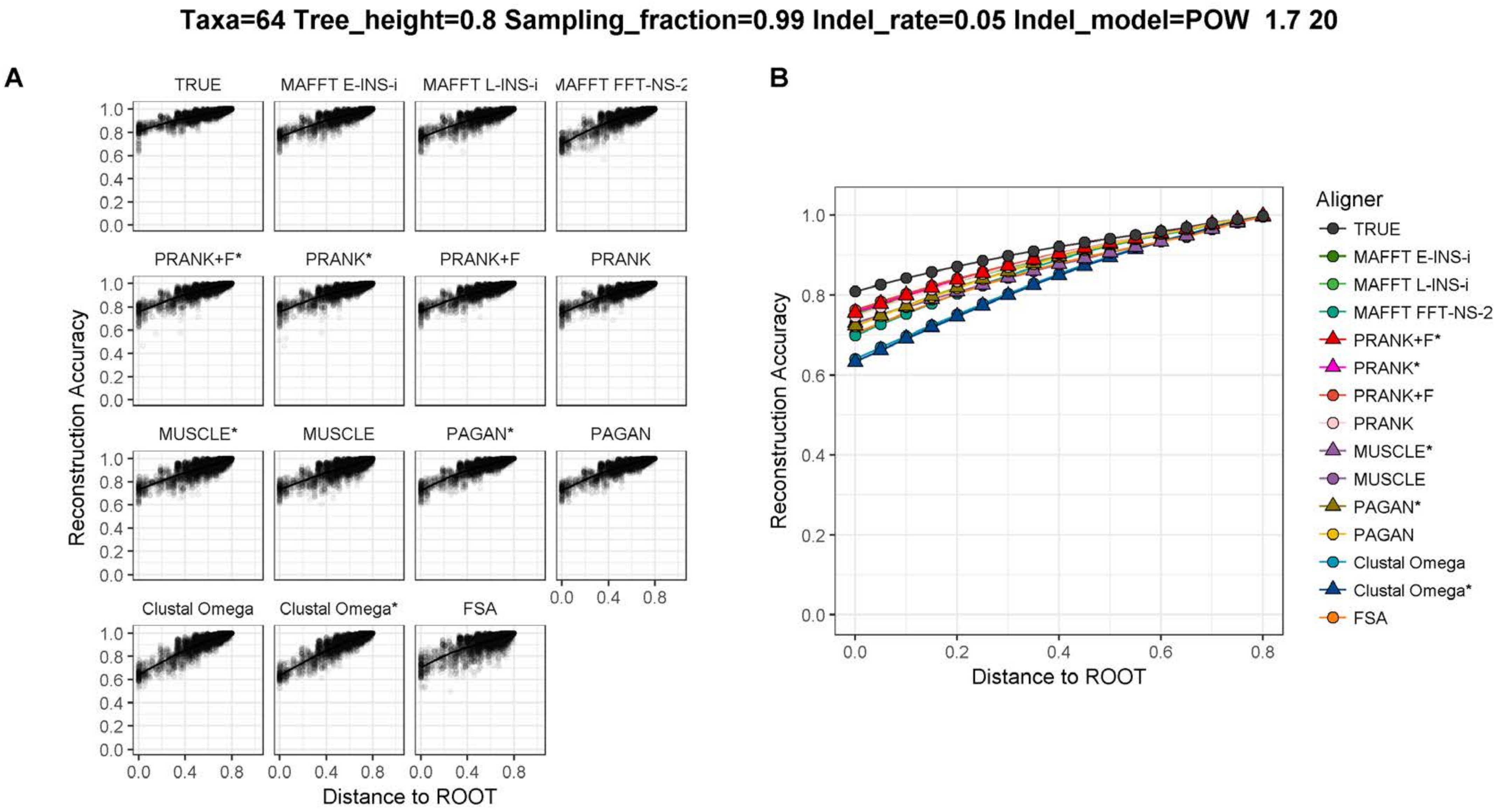
Taxa=64 Tree_height=0.8 Sampling_fraction=0.99 Indel_rate=0.05 Indel_model=POW 1.7 20
A
B
TRUE
MAFFT E-INS-i
MAFFT L-INS-i
MAFFT FFT-NS-2
PRANK+F*
PRANK*
PRANK+F
PRANK
MUSCLE*
MUSCLE
PAGAN*
PAGAN
Clustal Omega
Clustal Omega*
FSA
Reconstruction Accuracy
Distance to ROOT
Aligner

Taxa=16 Tree_height=1 Sampling_fraction=0.01 Indel_rate=0.01 Indel_model=POW 1.7 20

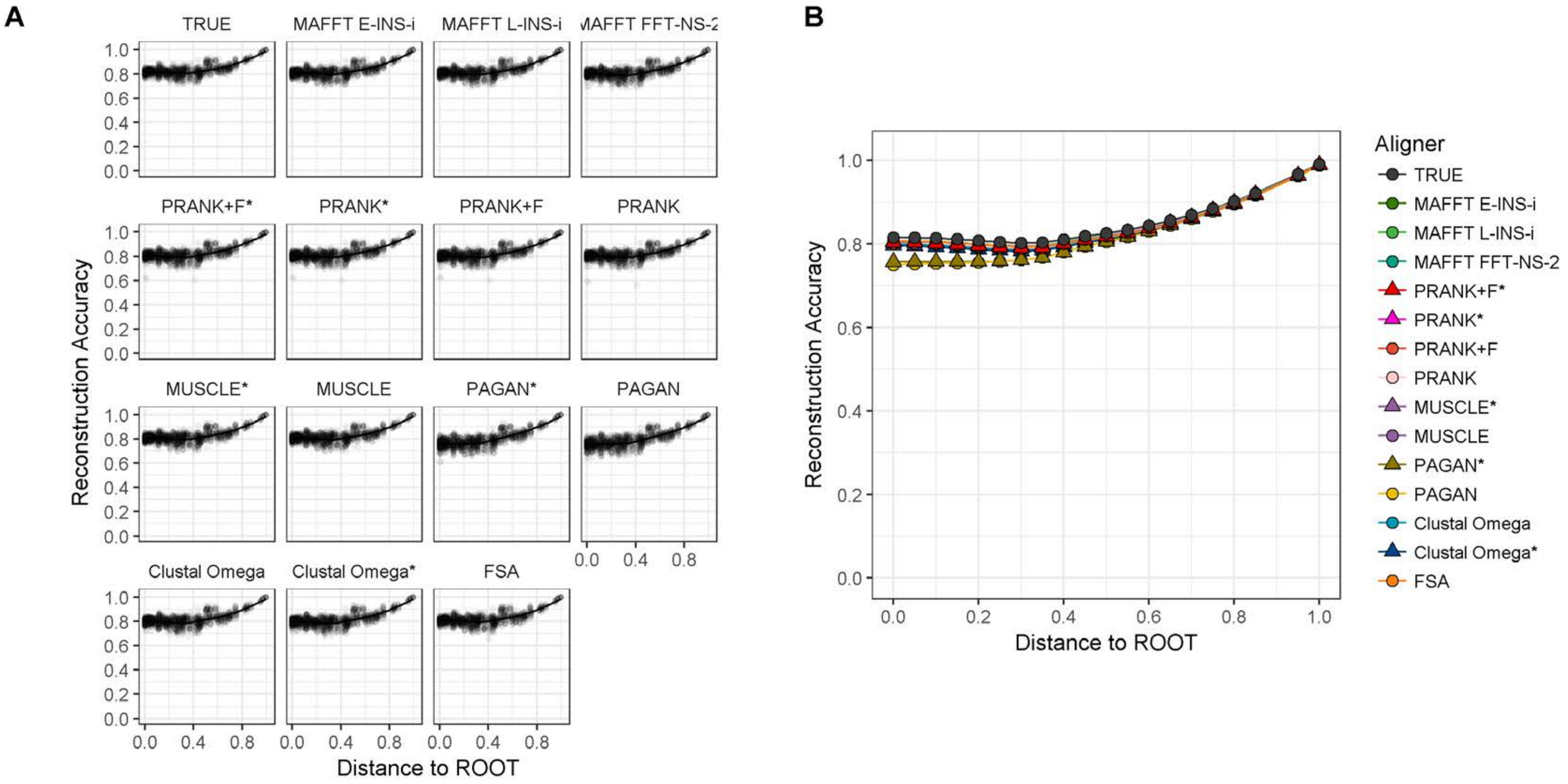

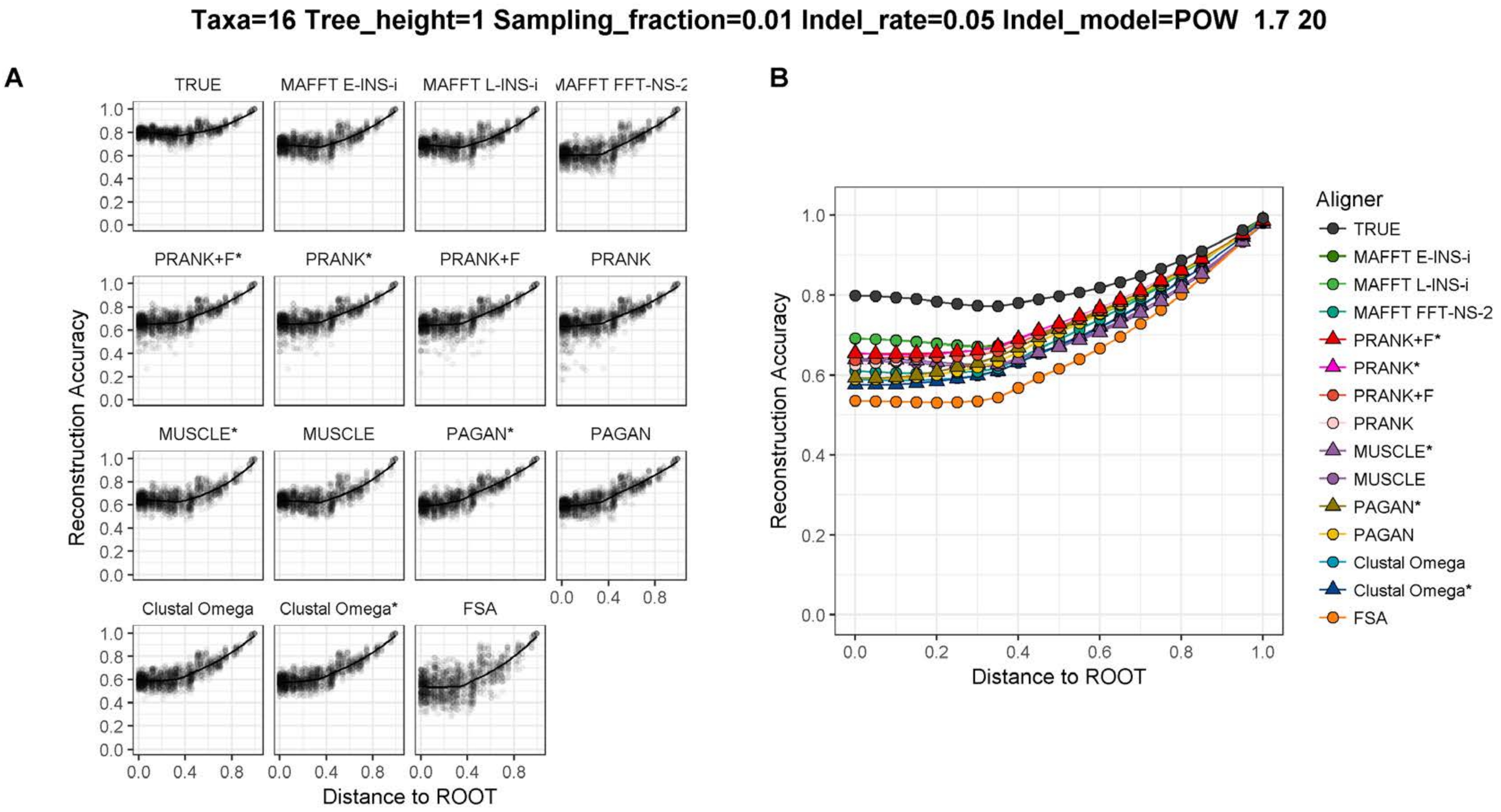
Taxa=16 Tree_height=1 Sampling_fraction=0.01 Indel_rate=0.05 Indel_model=POW 1.7 20
A
TRUE
MAFFT E-INS-i
MAFFT L-INS-i
PRANK+F*
PRANK*
PRANK+F
PRANK
MUSCLE*
MUSCLE
PAGAN*
PAGAN
Clustal Omega
Clustal Omega*
FSA
Reconstruction Accuracy
Distance to ROOT
B
Reconstruction Accuracy
Distance to ROOT
Aligner
TRUE
MAFFT E-INS-i
MAFFT L-INS-i
MAFFT FFT-NS-2
PRANK+F*
PRANK*
PRANK+F
PRANK
MUSCLE*
MUSCLE
PAGAN*
PAGAN
Clustal Omega
Clustal Omega*
FSA

Taxa=16 Tree_height=1 Sampling_fraction=0.25 Indel_rate=0.01 Indel_model=POW 1.7 20

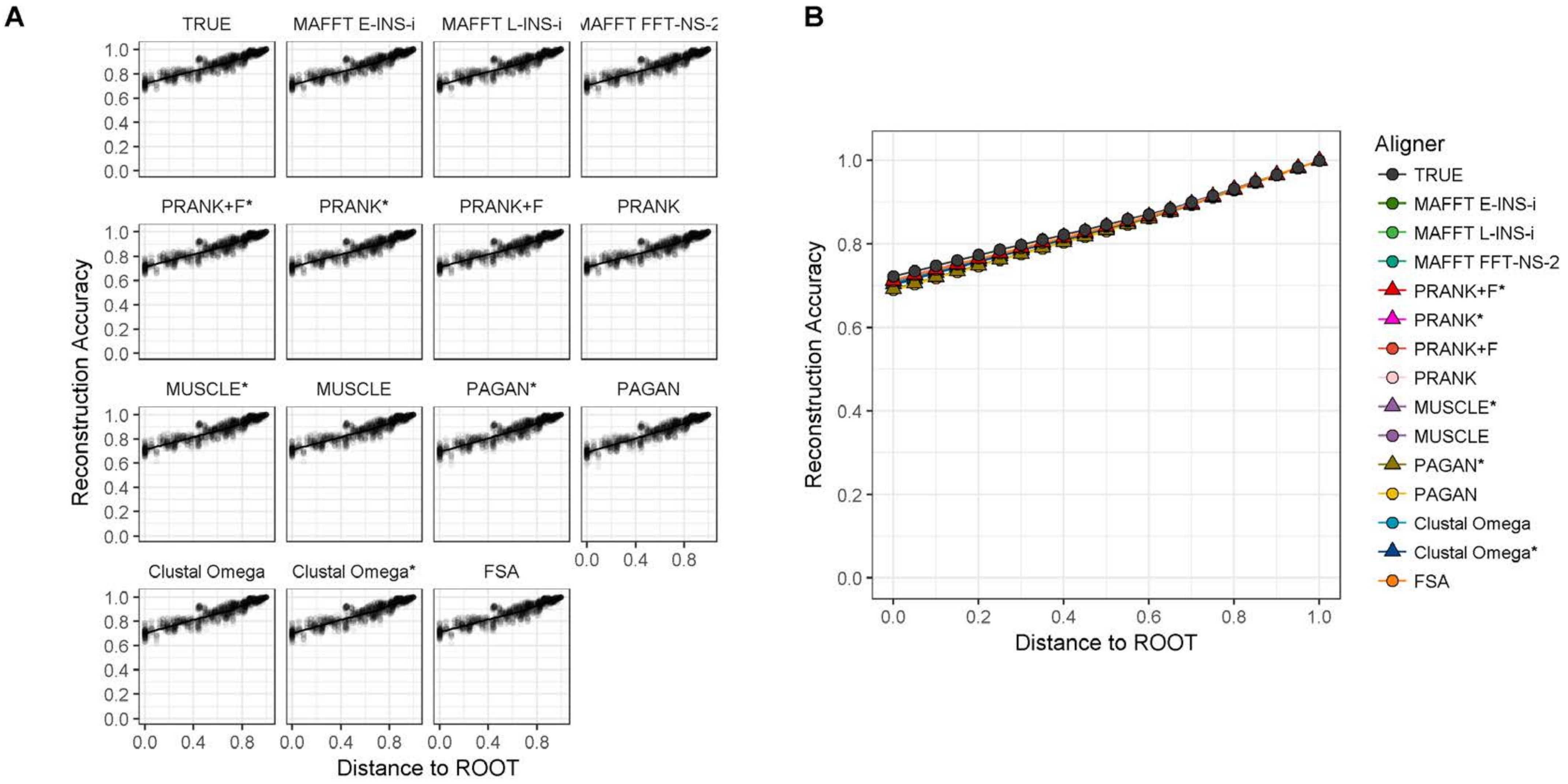

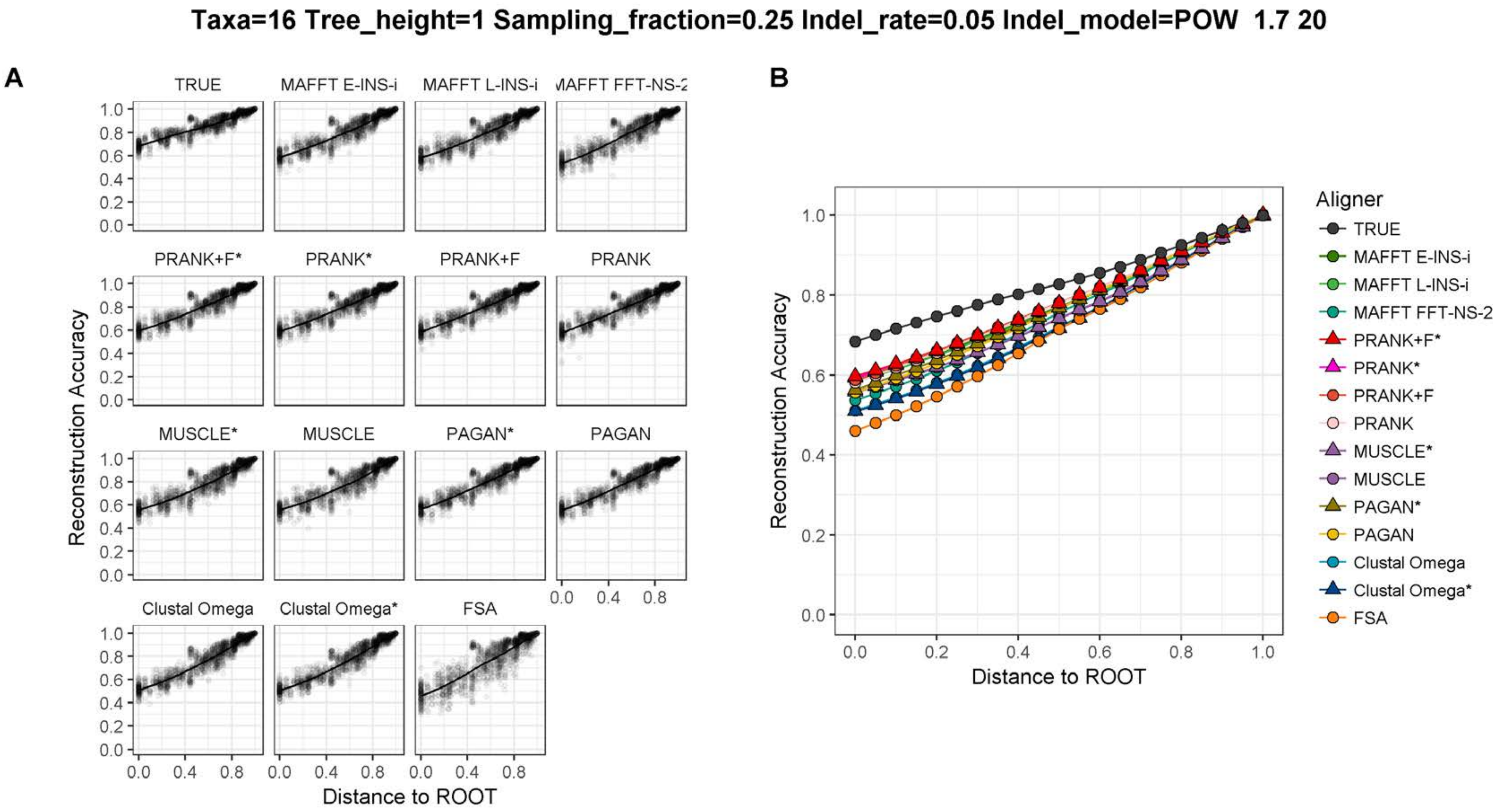
Taxa=16 Tree_height=1 Sampling_fraction=0.25 Indel_rate=0.05 Indel_model=POW 1.7 20
A
TRUE
MAFFT E-INS-i
MAFFT L-INS-i
MAFFT FFT-NS-2
PRANK+F*
PRANK*
PRANK+F
PRANK
MUSCLE*
MUSCLE
PAGAN*
PAGAN
Clustal Omega
Clustal Omega*
FSA
Reconstruction Accuracy
Distance to ROOT
B
Reconstruction Accuracy
Distance to ROOT
Aligner
TRUE
MAFFT E-INS-i
MAFFT L-INS-i
MAFFT FFT-NS-2
PRANK+F*
PRANK*
PRANK+F
PRANK
MUSCLE*
MUSCLE
PAGAN*
PAGAN
Clustal Omega
Clustal Omega*
FSA

Taxa=16 Tree_height=1 Sampling_fraction=0.99 Indel_rate=0.01 Indel_model=POW 1.7 20

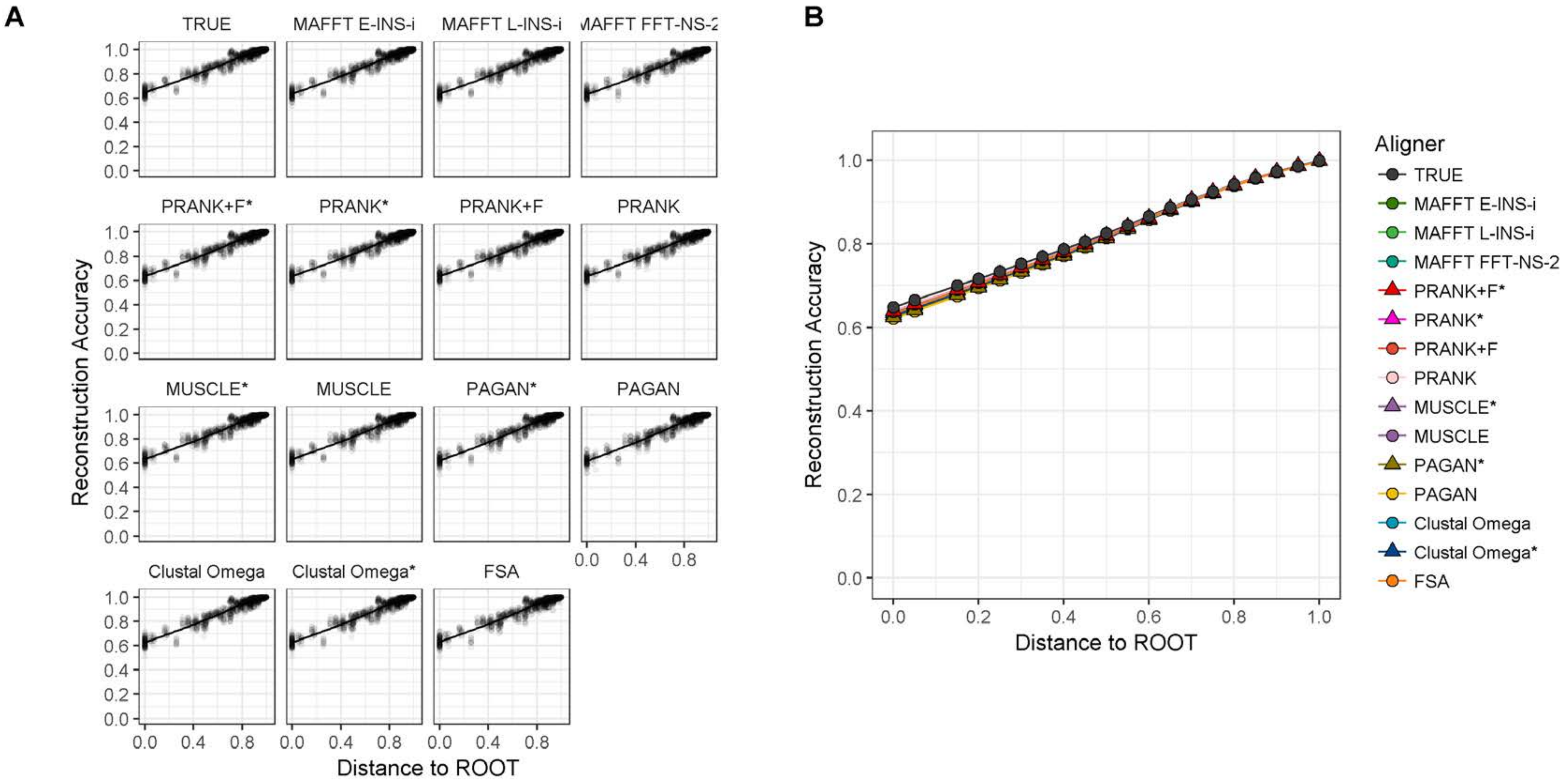

Taxa=16 Tree_height=1 Sampling_fraction=0.99 Indel_rate=0.05 Indel_model=POW 1.7 20

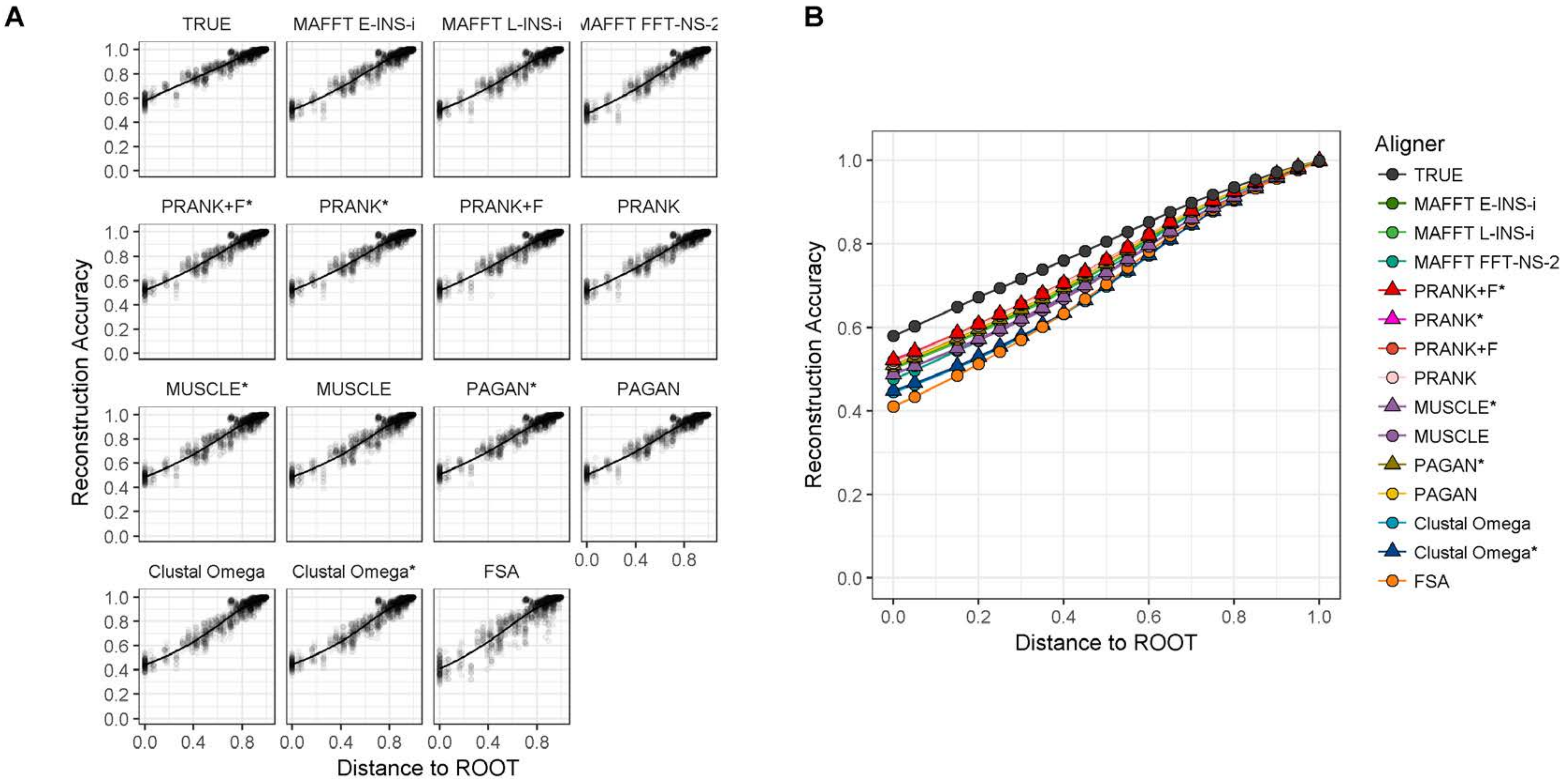

Taxa=32 Tree_height=1 Sampling_fraction=0.01 Indel_rate=0.01 Indel_model=POW 1.7 20

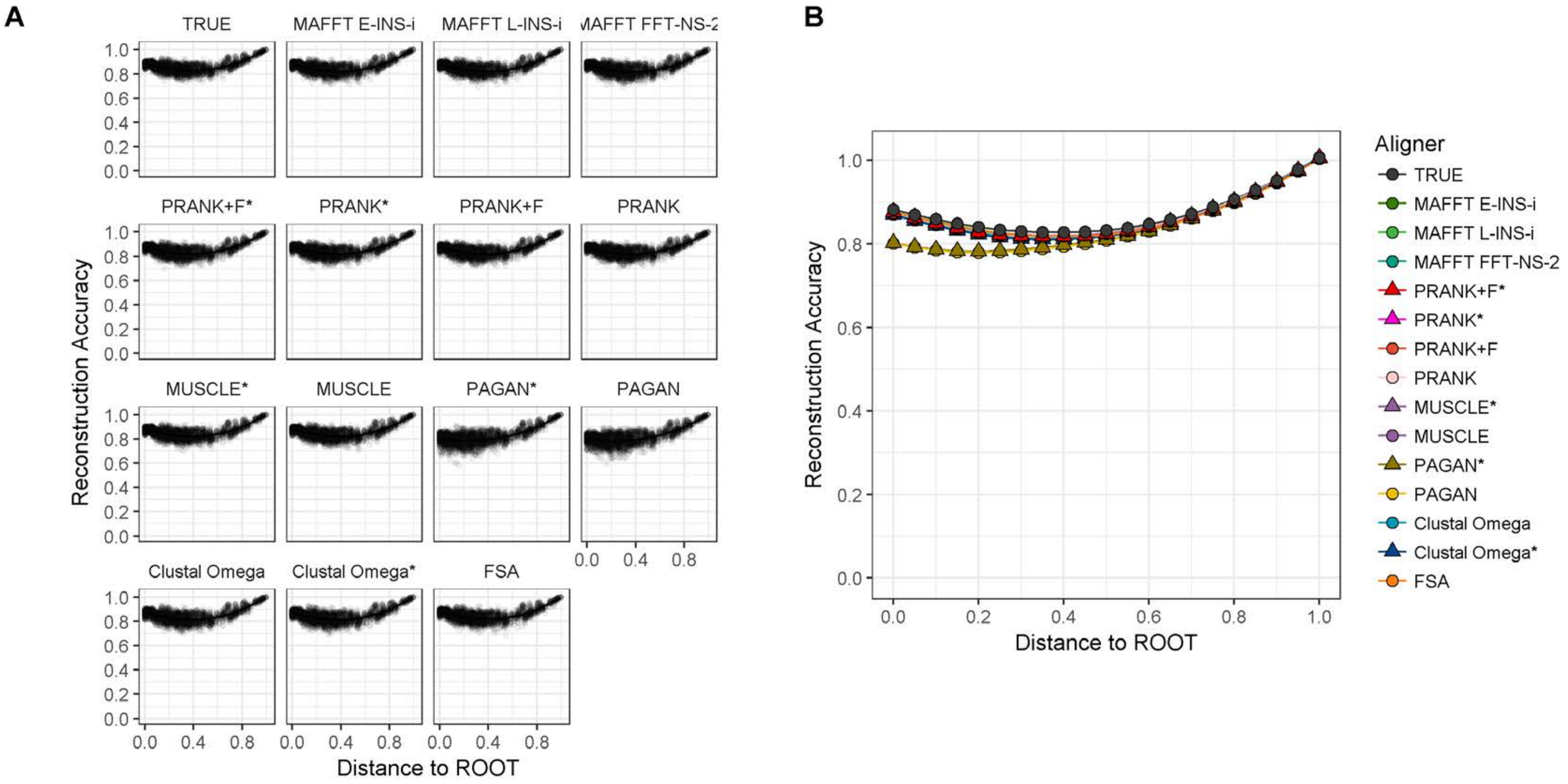

Taxa=32 Tree_height=1 Sampling_fraction=0.01 Indel_rate=0.05 Indel_model=POW 1.7 20

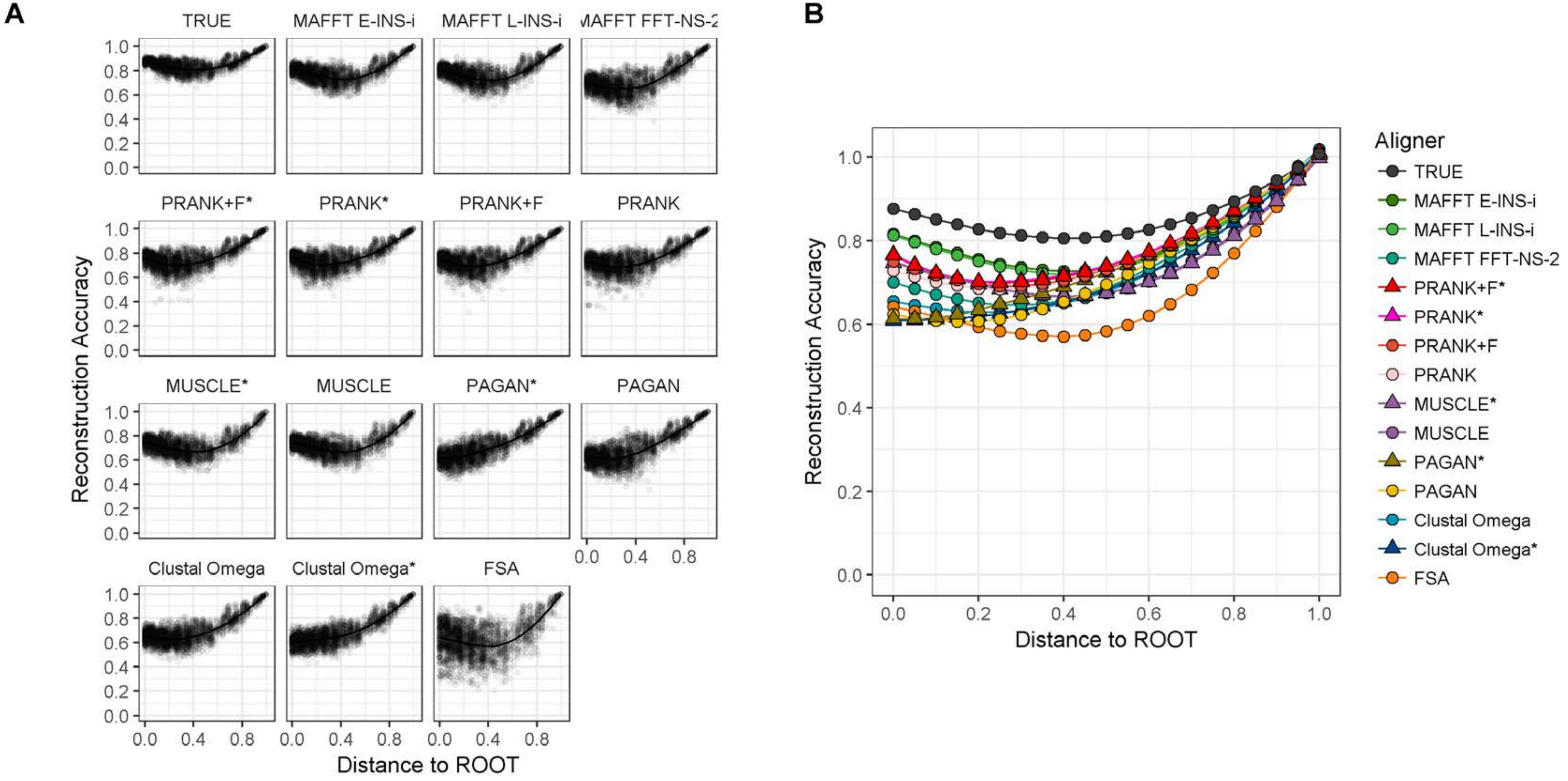

Taxa=32 Tree_height=1 Sampling_fraction=0.25 Indel_rate=0.01 Indel_model=POW 1.7 20

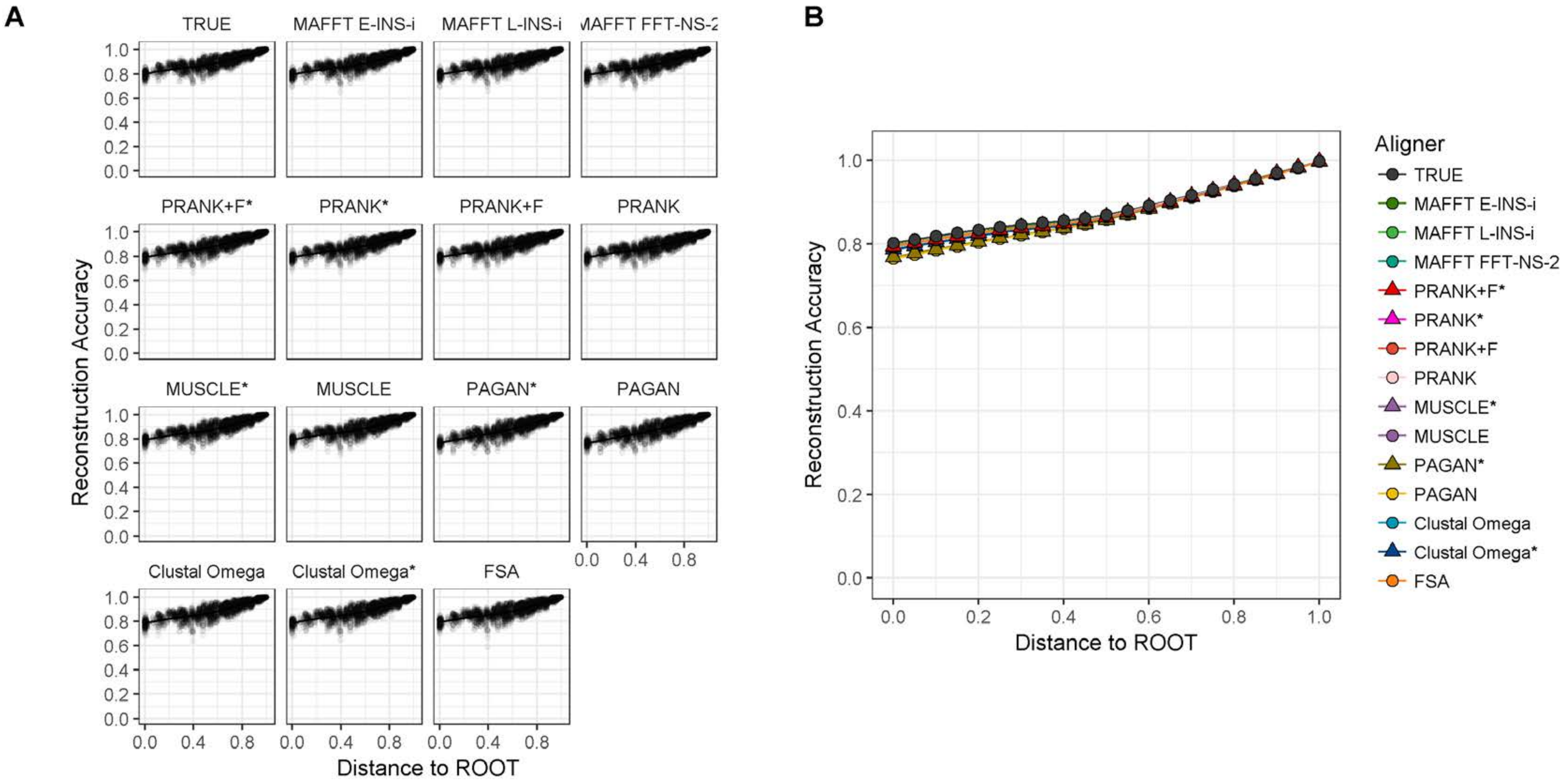

Taxa=32 Tree_height=1 Sampling_fraction=0.25 Indel_rate=0.05 Indel_model=POW 1.7 20

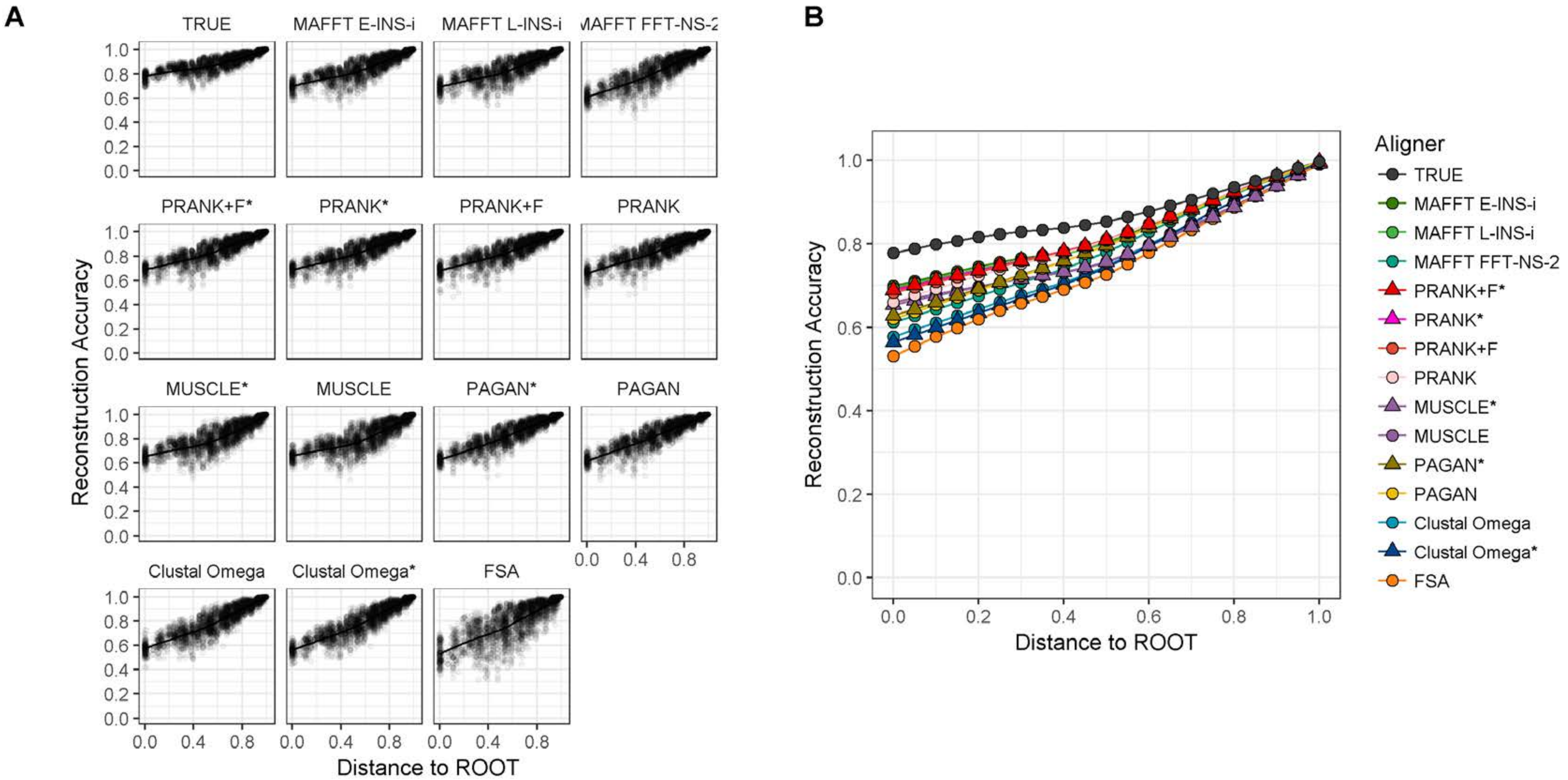

Taxa=32 Tree_height=1 Sampling_fraction=0.99 Indel_rate=0.01 Indel_model=POW 1.7 20

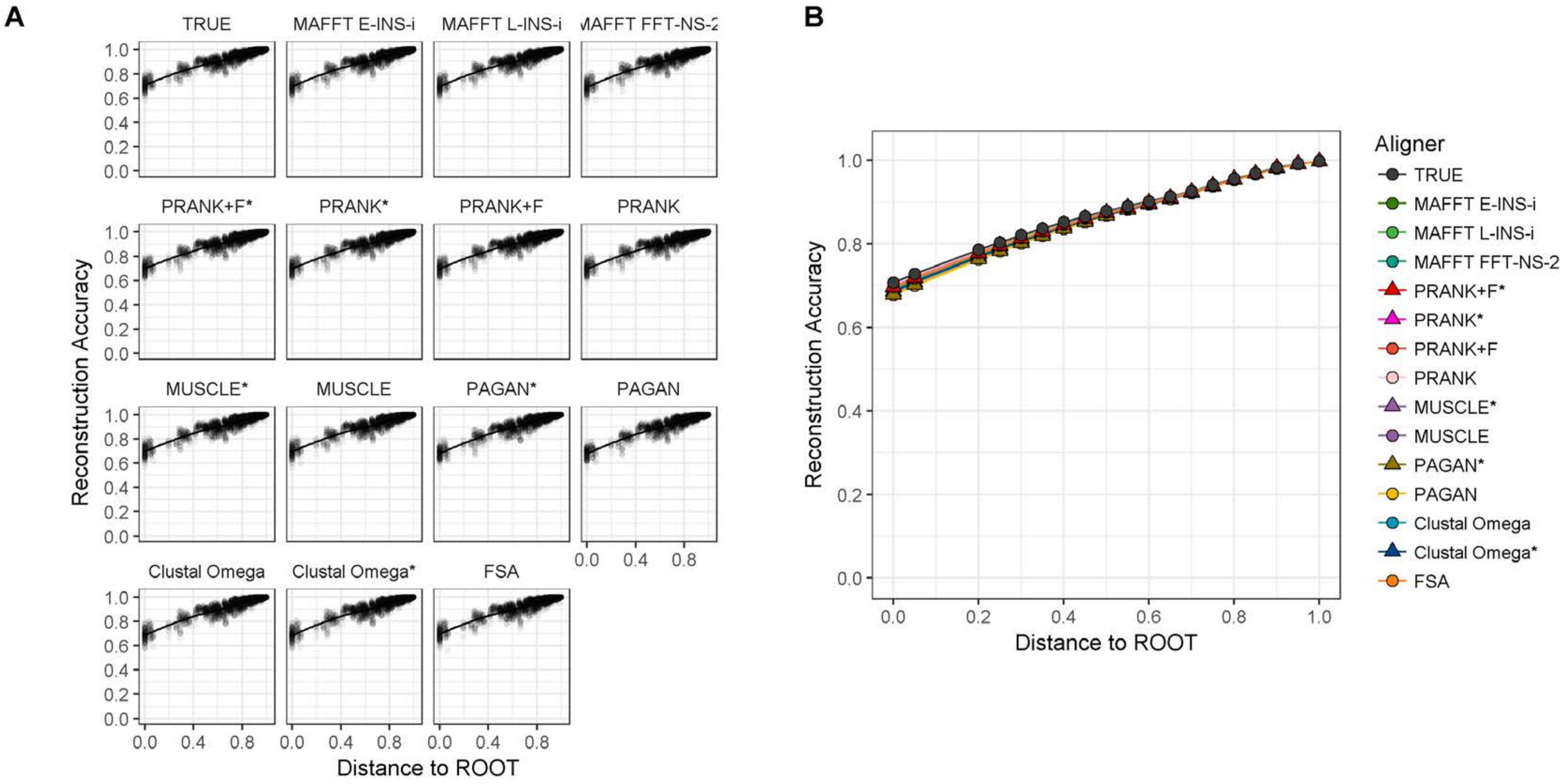

Taxa=32 Tree_height=1 Sampling_fraction=0.99 Indel_rate=0.05 Indel_model=POW 1.7 20

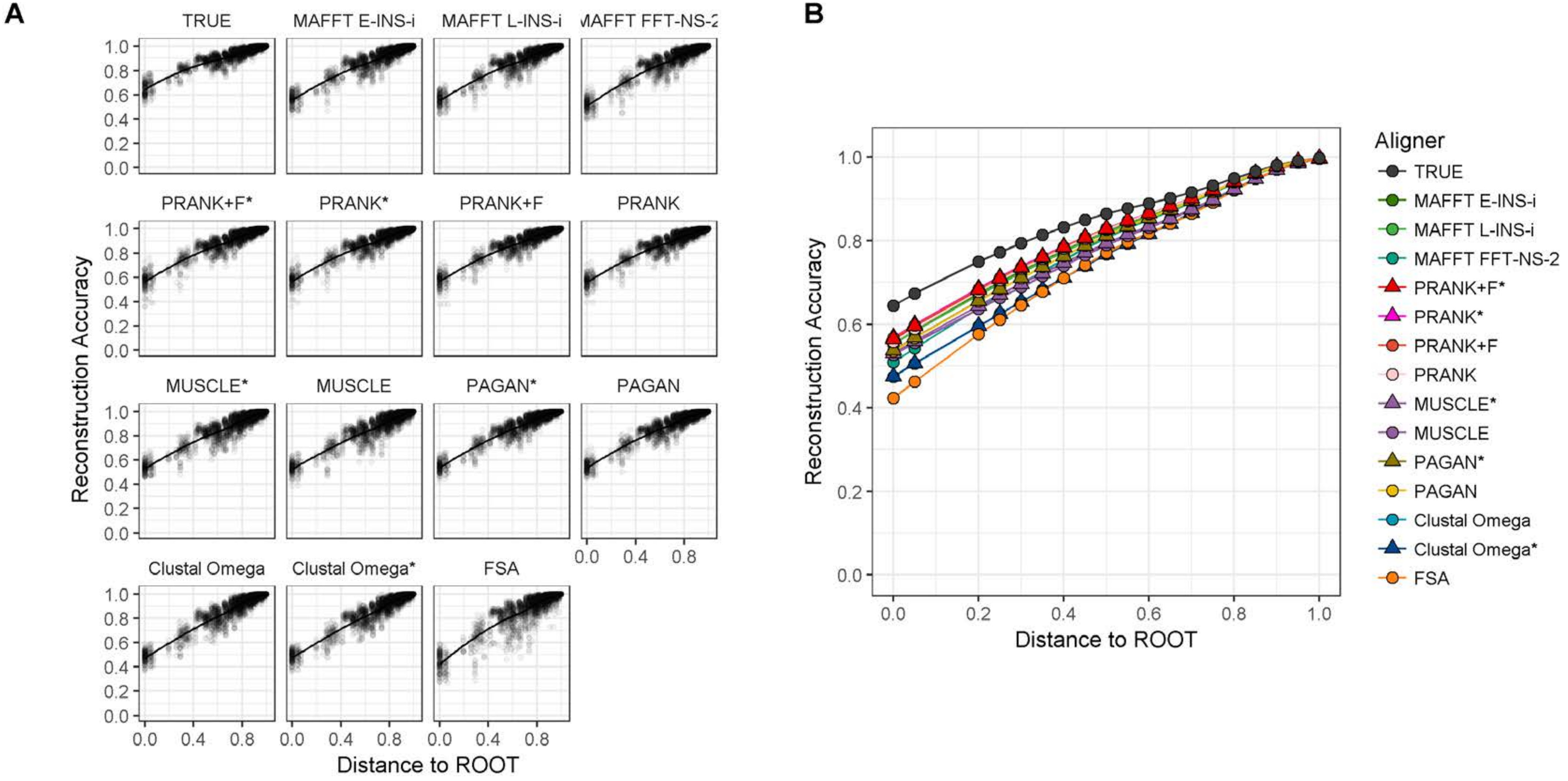

Taxa=64 Tree_height=1 Sampling_fraction=0.01 Indel_rate=0.01 Indel_model=POW 1.7 20

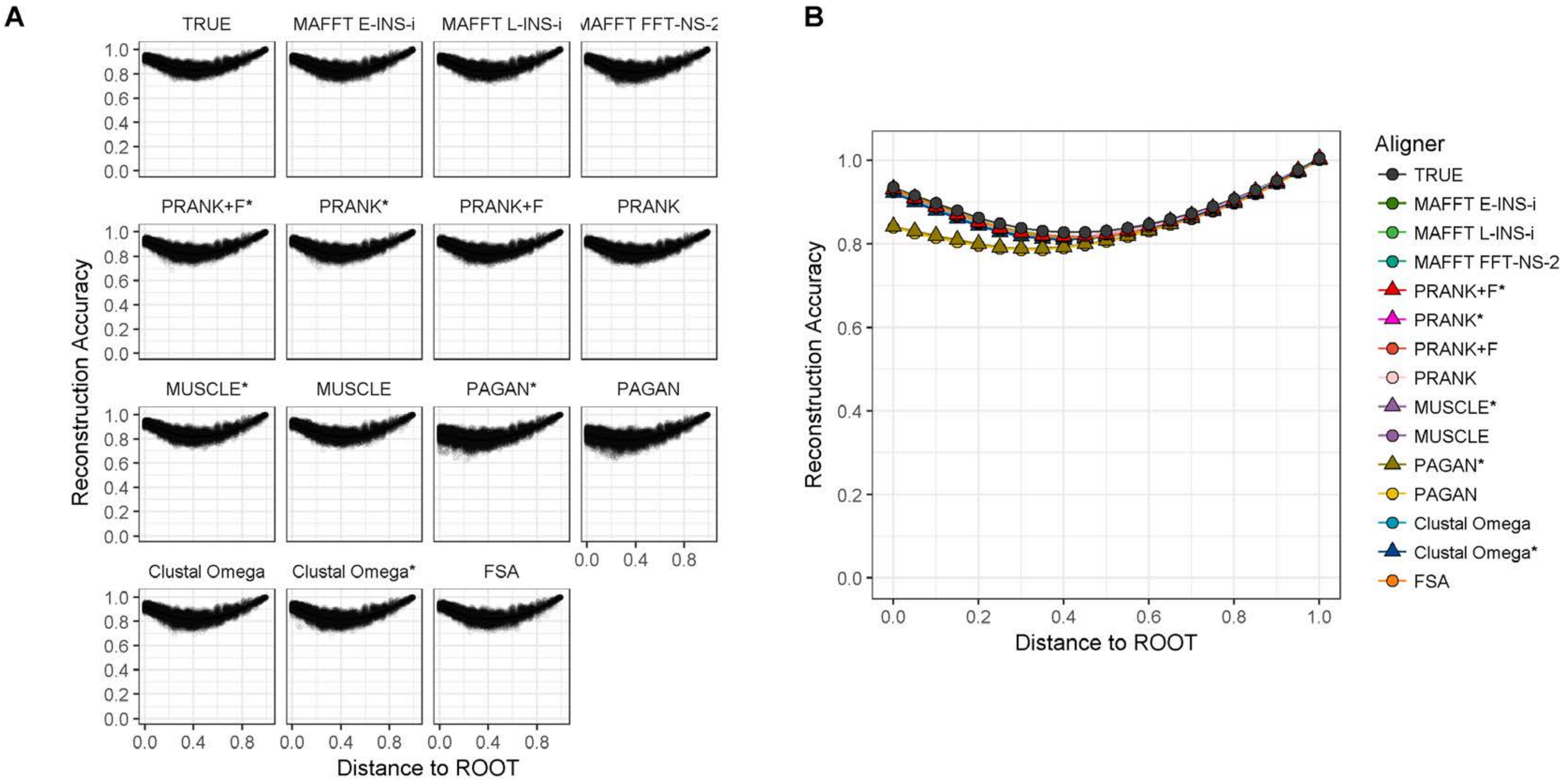

Taxa=64 Tree_height=1 Sampling_fraction=0.01 Indel_rate=0.05 Indel_model=POW 1.7 20

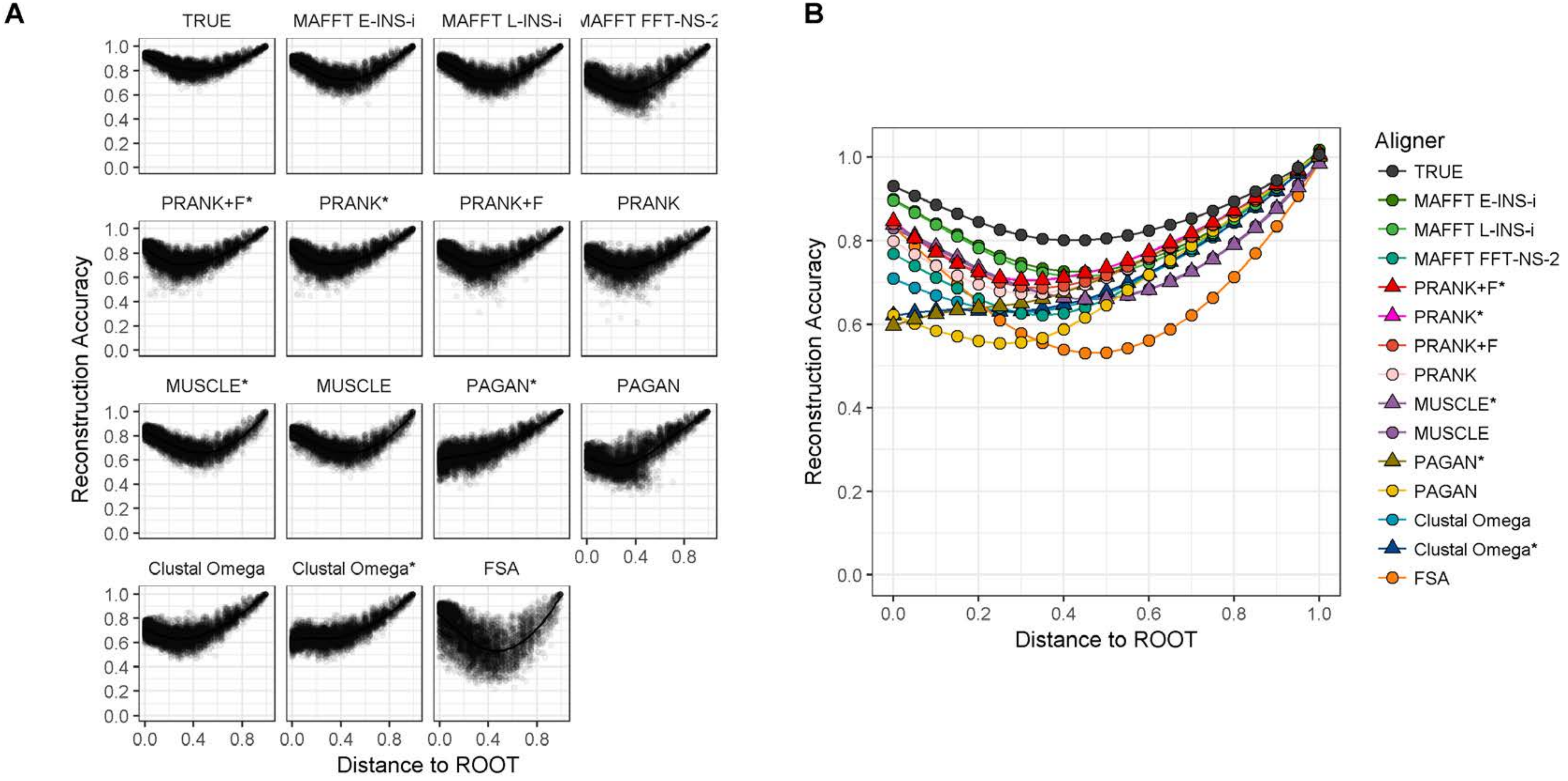

Taxa=64 Tree_height=1 Sampling_fraction=0.25 Indel_rate=0.01 Indel_model=POW 1.7 20

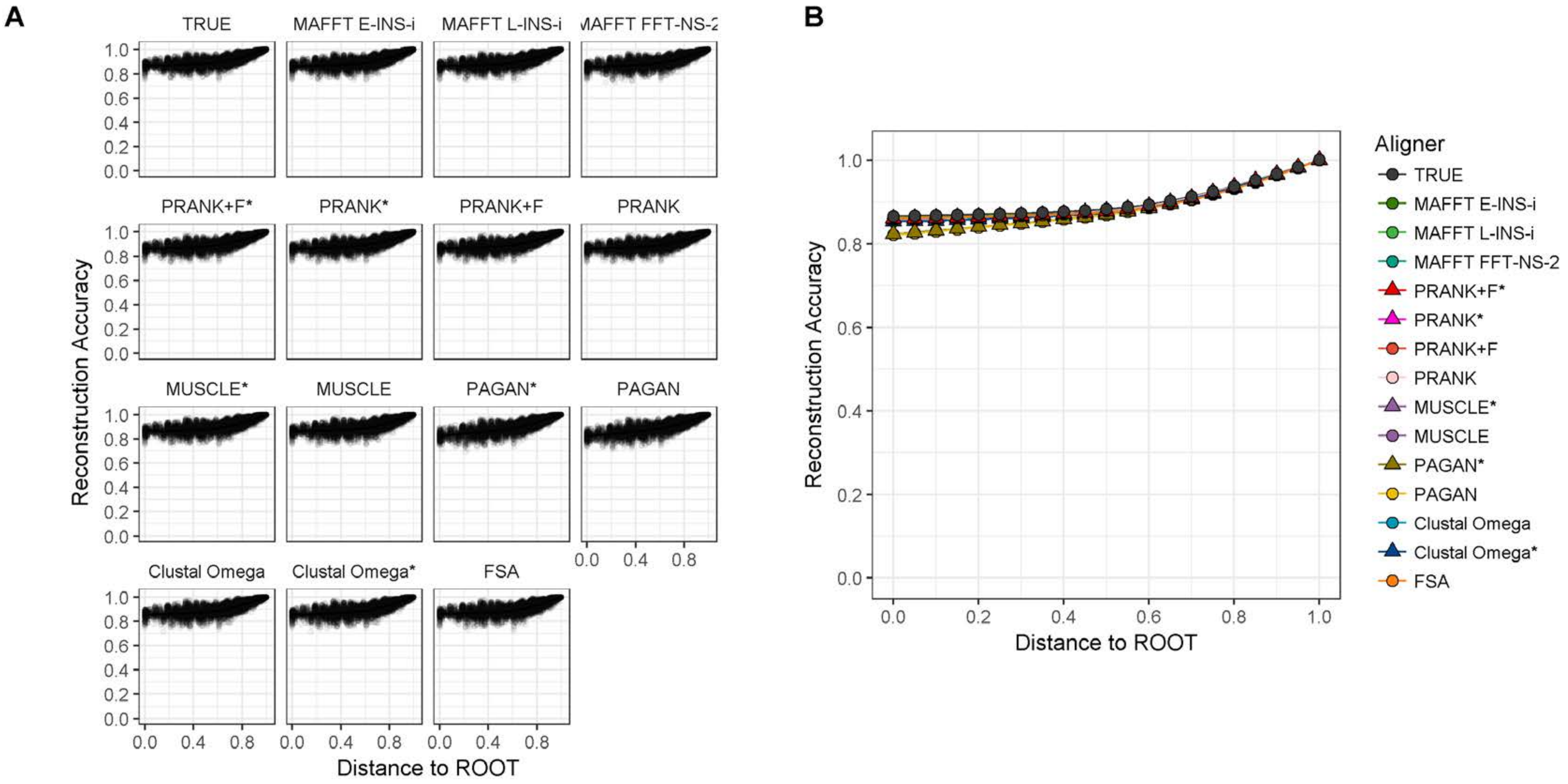

Taxa=64 Tree_height=1 Sampling_fraction=0.25 Indel_rate=0.05 Indel_model=POW 1.7 20

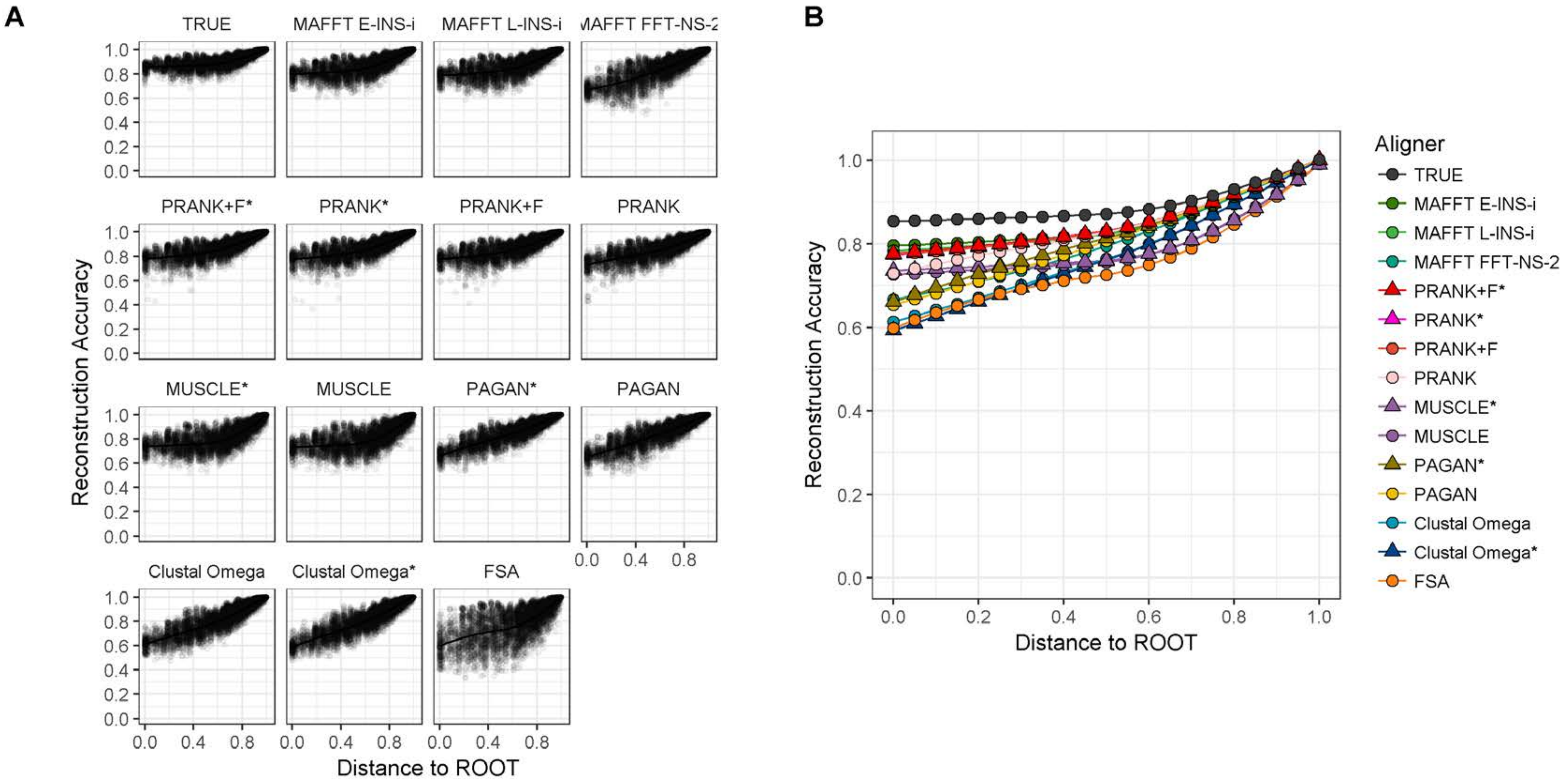

Taxa=64 Tree_height=1 Sampling_fraction=0.99 Indel_rate=0.01 Indel_model=POW 1.7 20

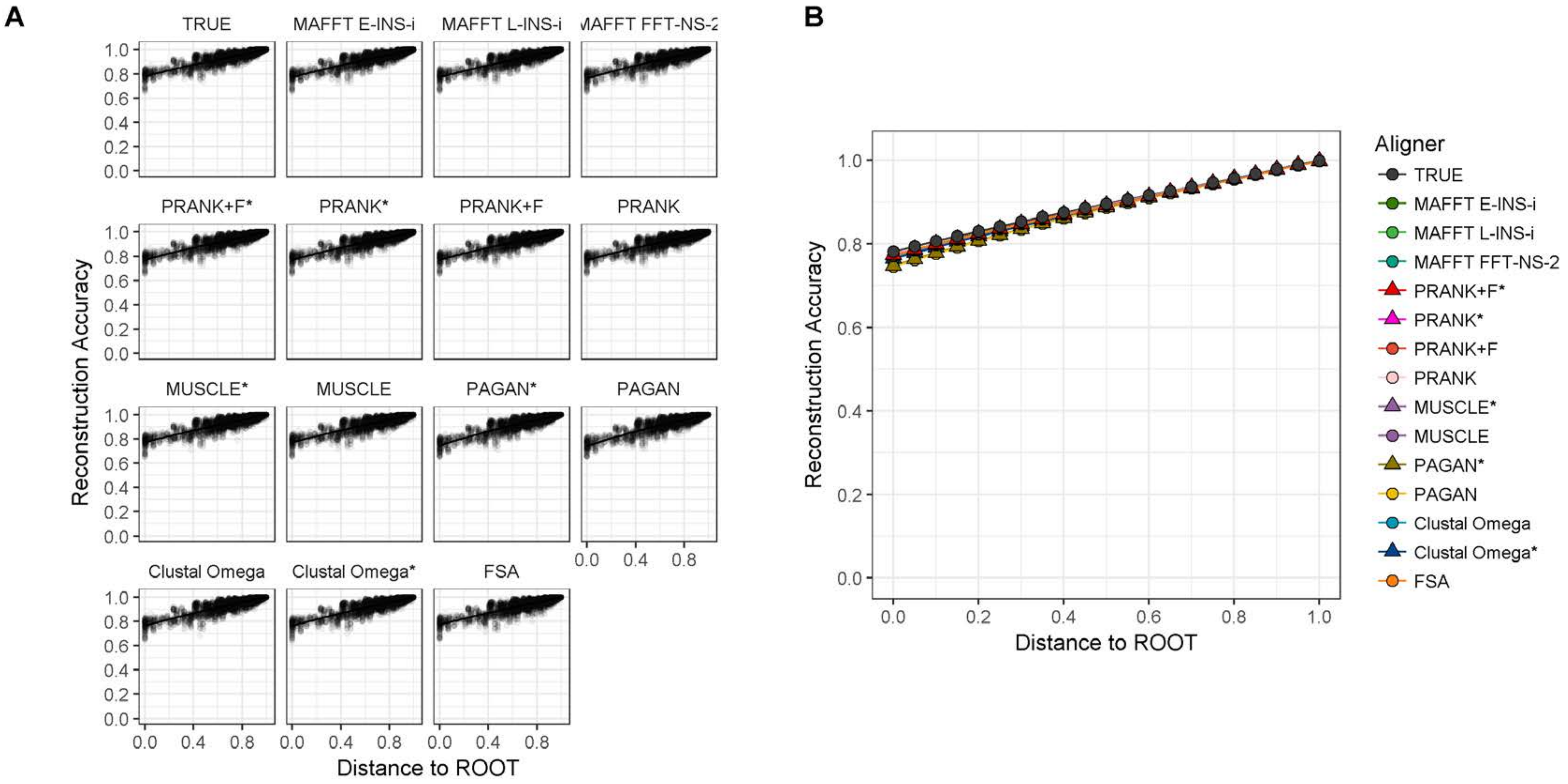

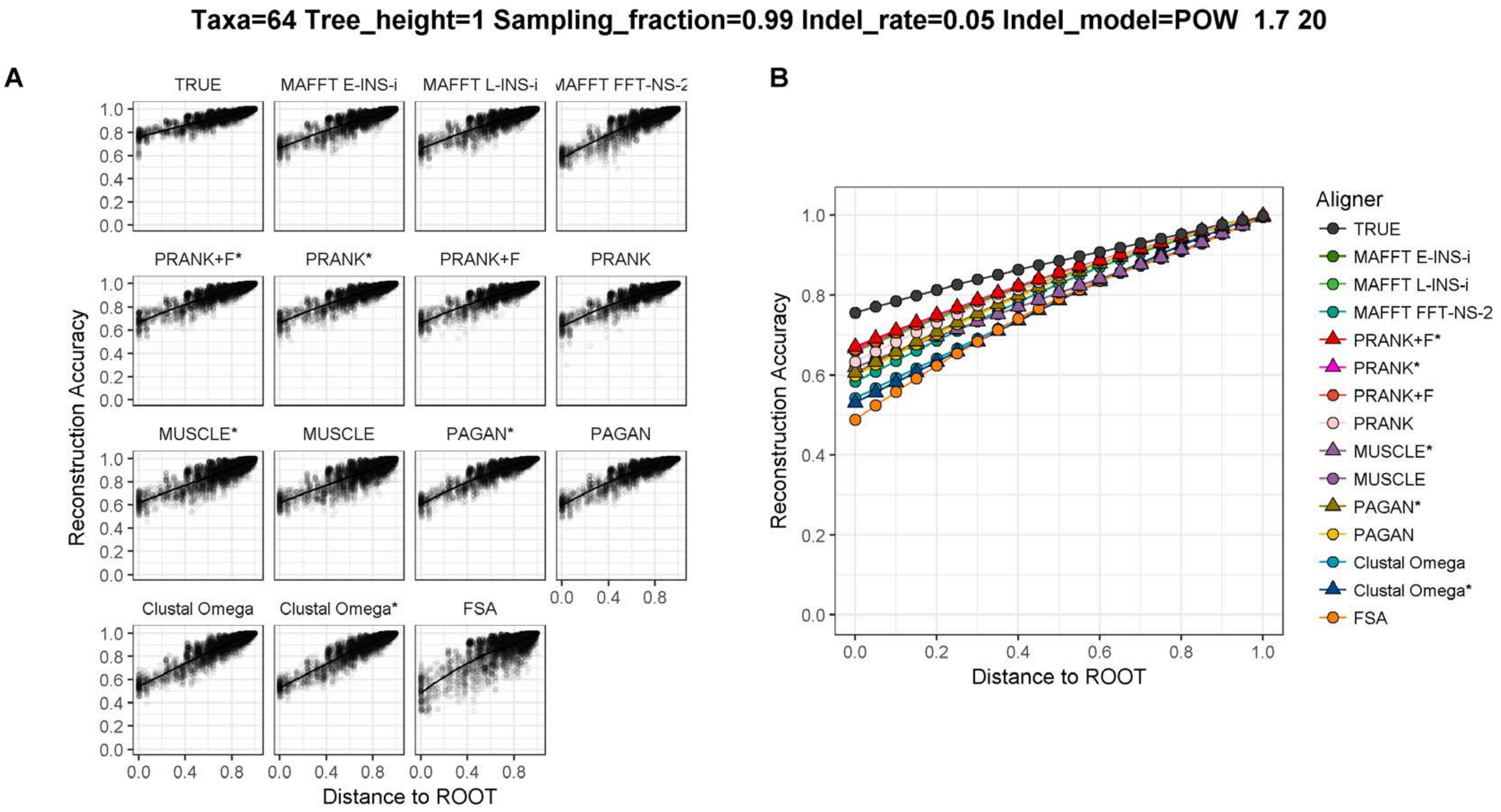
Taxa=64 Tree_height=1 Sampling_fraction=0.99 Indel_rate=0.05 Indel_model=POW 1.7 20
A
B
TRUE
MAFFT E-INS-i
MAFFT L-INS-i
MAFFT FFT-NS-2
PRANK+F*
PRANK*
PRANK+F
PRANK
MUSCLE*
MUSCLE
PAGAN*
PAGAN
Clustal Omega
Clustal Omega*
FSA
Reconstruction Accuracy
Distance to ROOT
Aligner

Taxa=16 Tree_height=1.2 Sampling_fraction=0.01 Indel_rate=0.01 Indel_model=POW 1.7 20

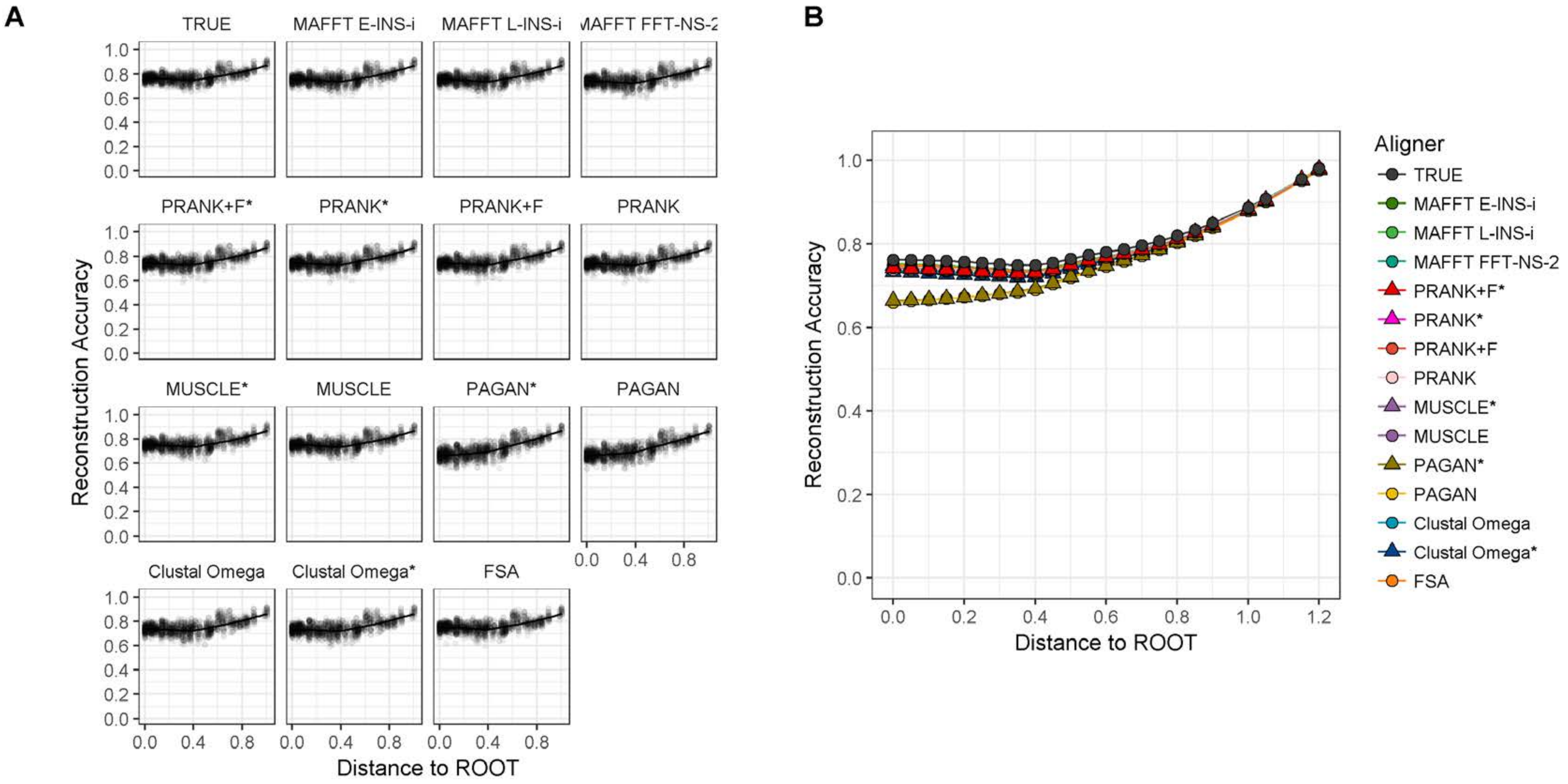

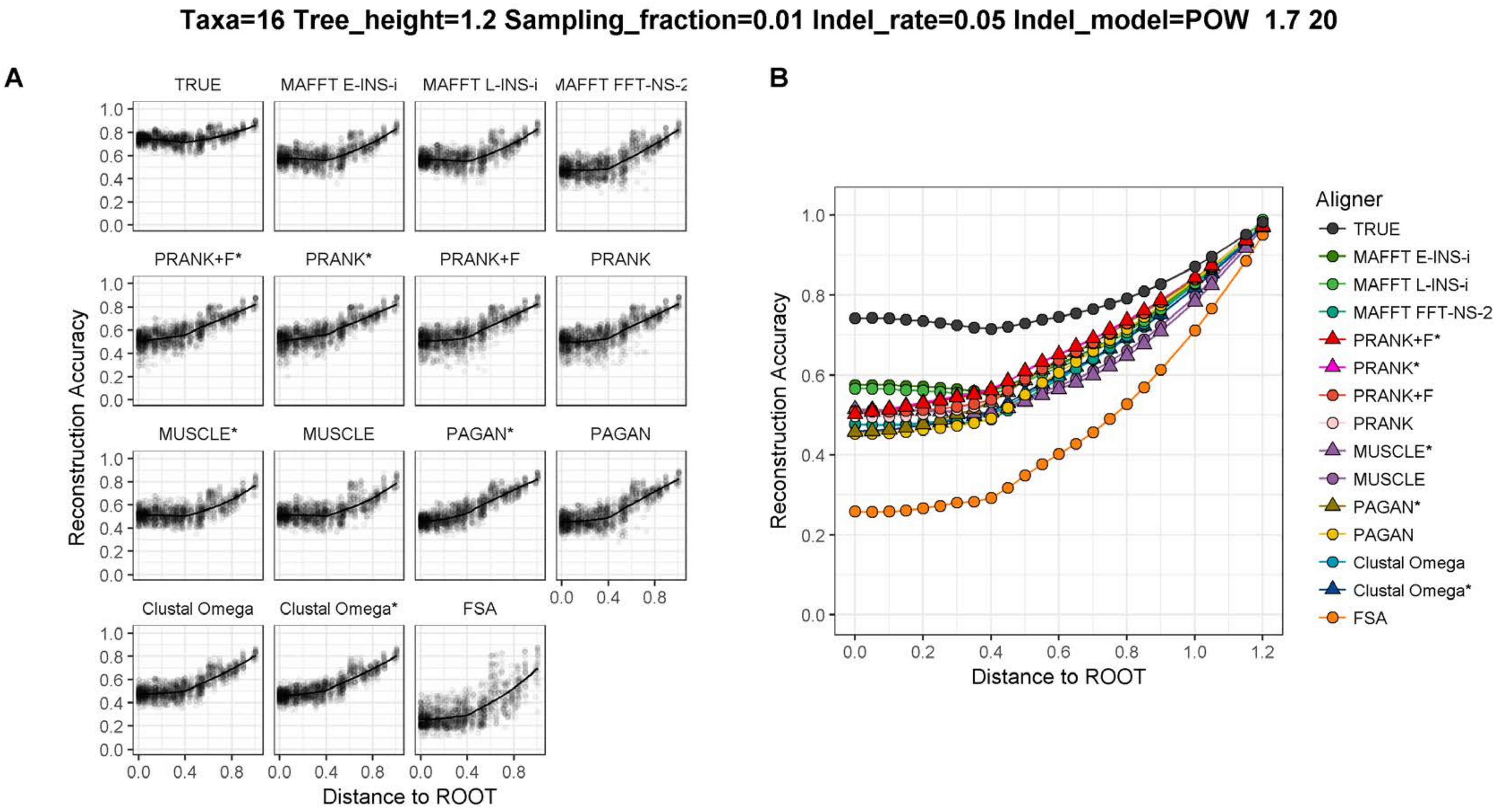

Taxa=16 Tree_height=1.2 Sampling_fraction=0.01 Indel_rate=0.05 Indel_model=POW 1.7 20
A
TRUE
MAFFT E-INS-i
MAFFT L-INS-i
MAFFT FFT-NS-2
PRANK+F*
PRANK*
PRANK+F
PRANK
MUSCLE*
MUSCLE
PAGAN*
PAGAN
Clustal Omega
Clustal Omega*
FSA
Reconstruction Accuracy
Distance to ROOT
B
Reconstruction Accuracy
Distance to ROOT
Aligner
TRUE
MAFFT E-INS-i
MAFFT L-INS-i
MAFFT FFT-NS-2
PRANK+F*
PRANK*
PRANK+F
PRANK
MUSCLE*
MUSCLE
PAGAN*
PAGAN
Clustal Omega
Clustal Omega*
FSA

Taxa=16 Tree_height=1.2 Sampling_fraction=0.25 Indel_rate=0.01 Indel_model=POW 1.7 20

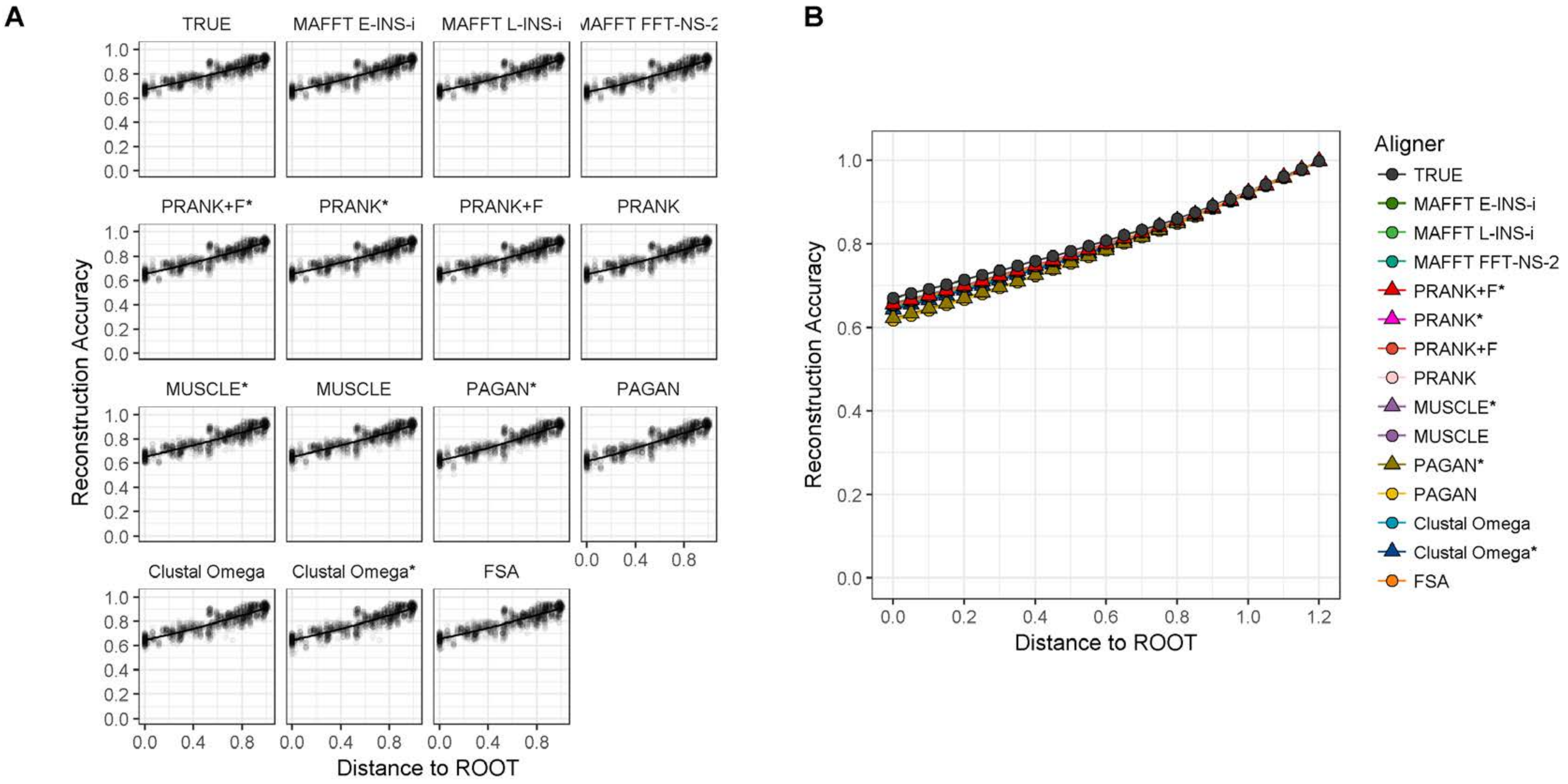

Taxa=16 Tree_height=1.2 Sampling_fraction=0.25 Indel_rate=0.05 Indel_model=POW 1.7 20

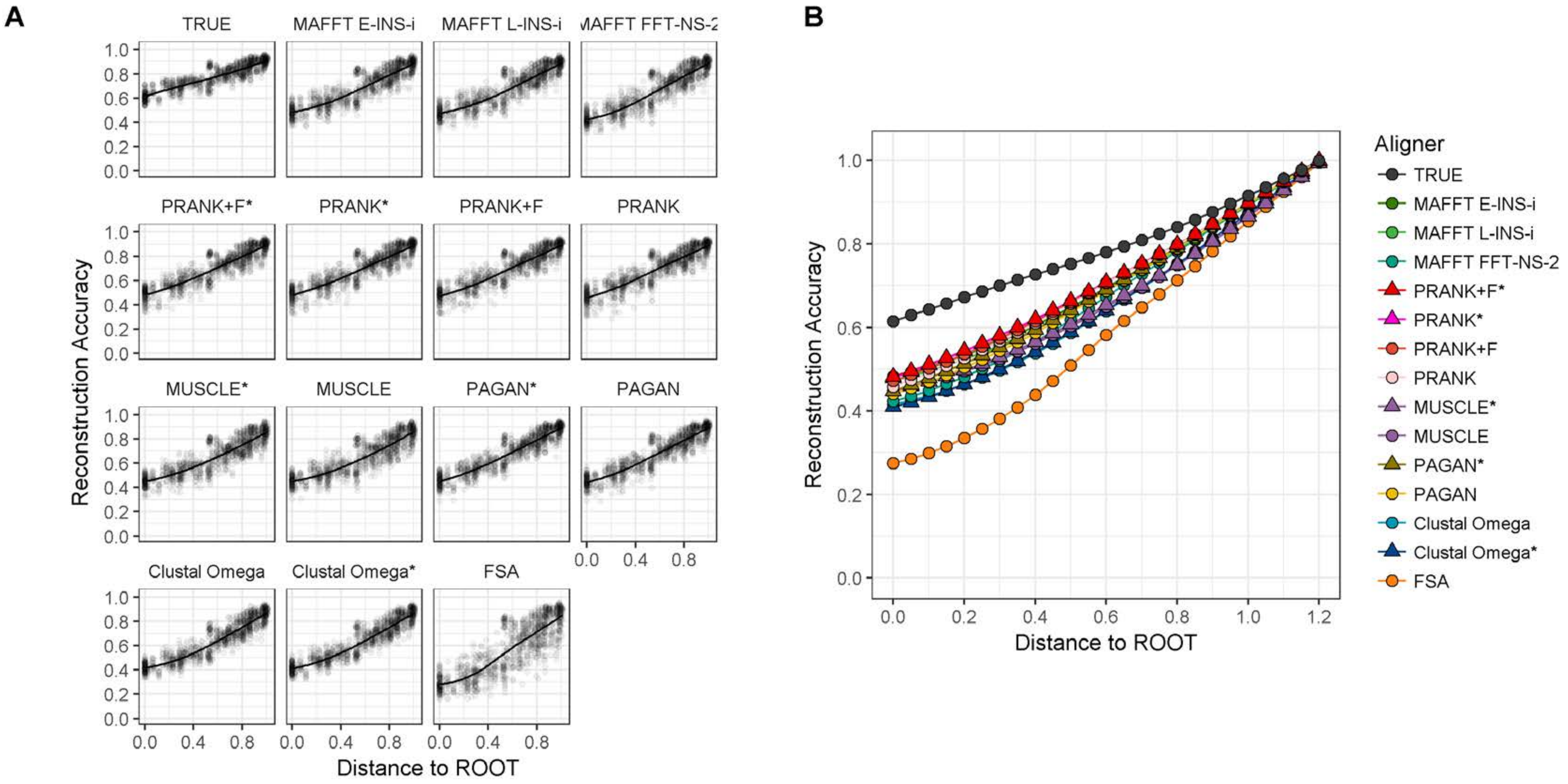

Taxa=16 Tree_height=1.2 Sampling_fraction=0.99 Indel_rate=0.01 Indel_model=POW 1.7 20

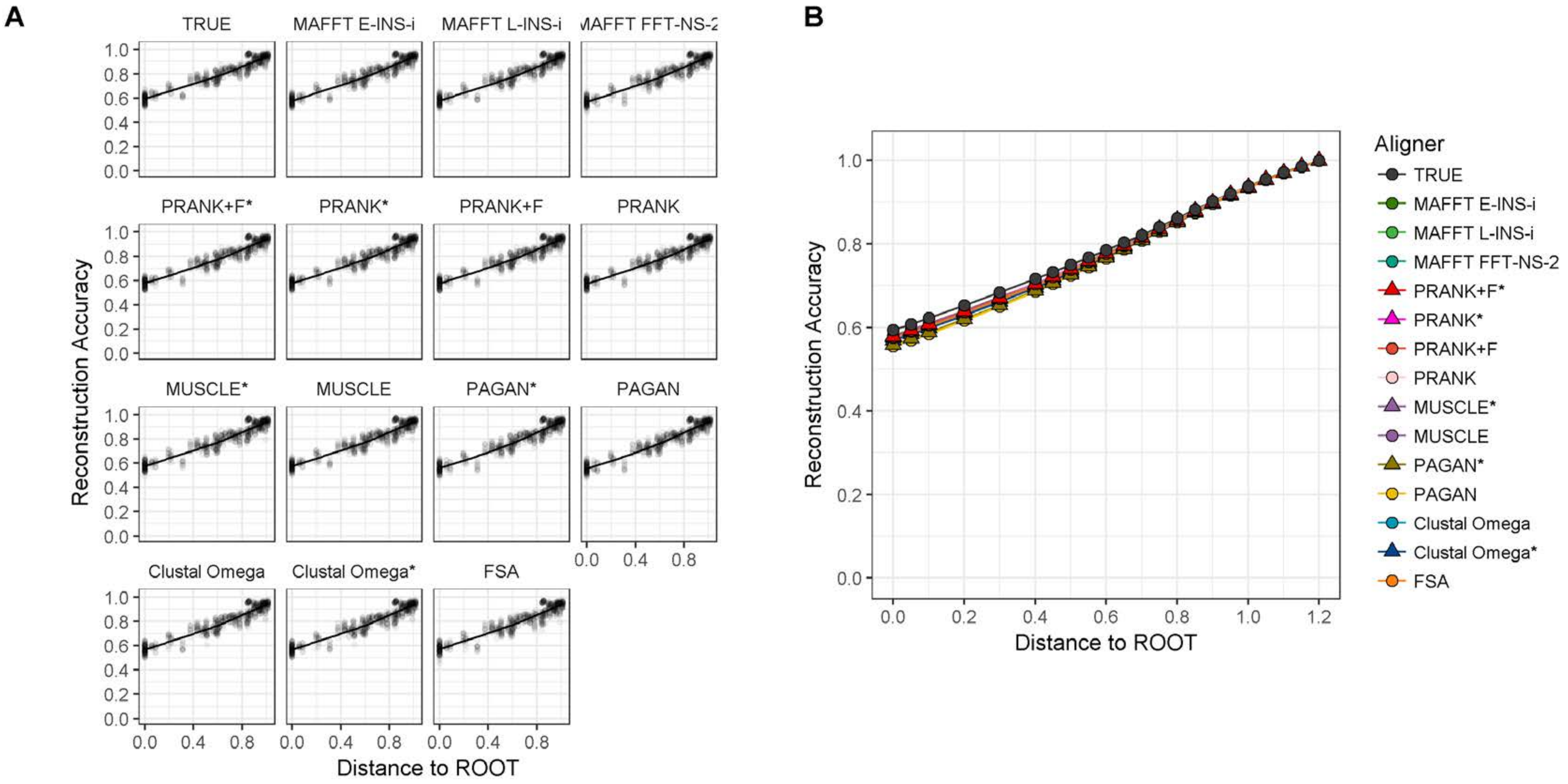

Taxa=16 Tree_height=1.2 Sampling_fraction=0.99 Indel_rate=0.05 Indel_model=POW 1.7 20

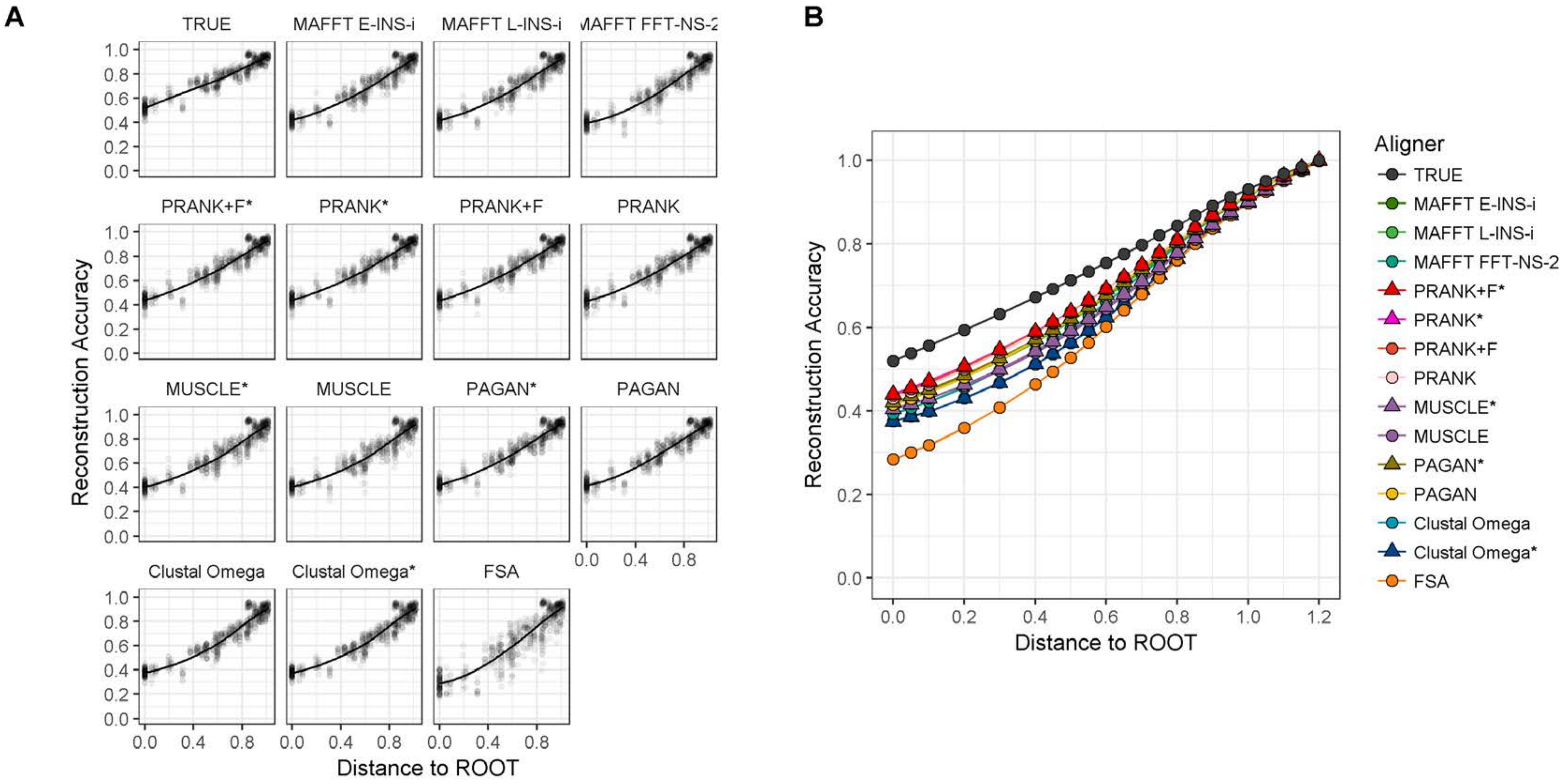

Taxa=32 Tree_height=1.2 Sampling_fraction=0.01 Indel_rate=0.01 Indel_model=POW 1.7 20

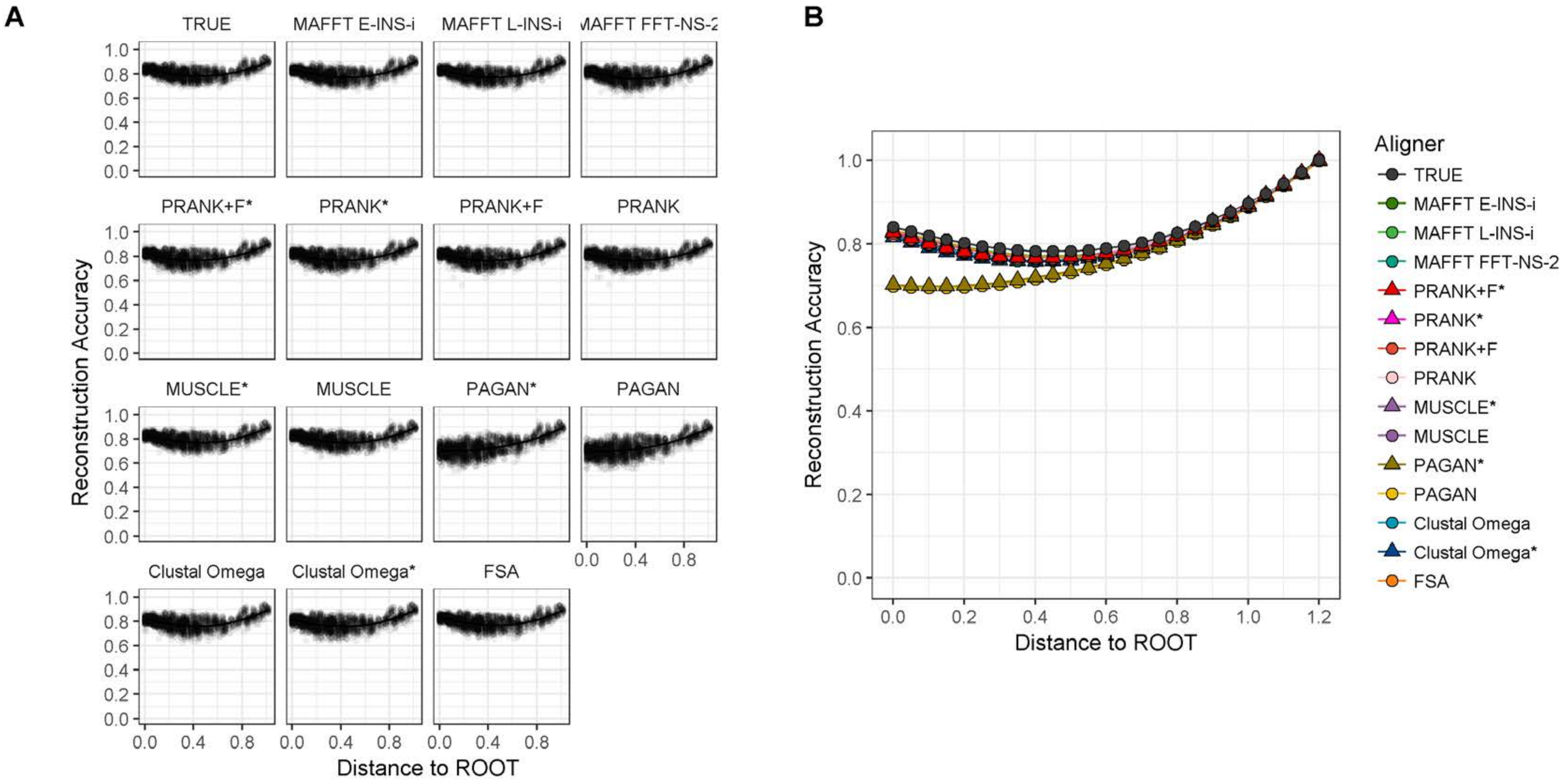

Taxa=32 Tree_height=1.2 Sampling_fraction=0.01 Indel_rate=0.05 Indel_model=POW 1.7 20

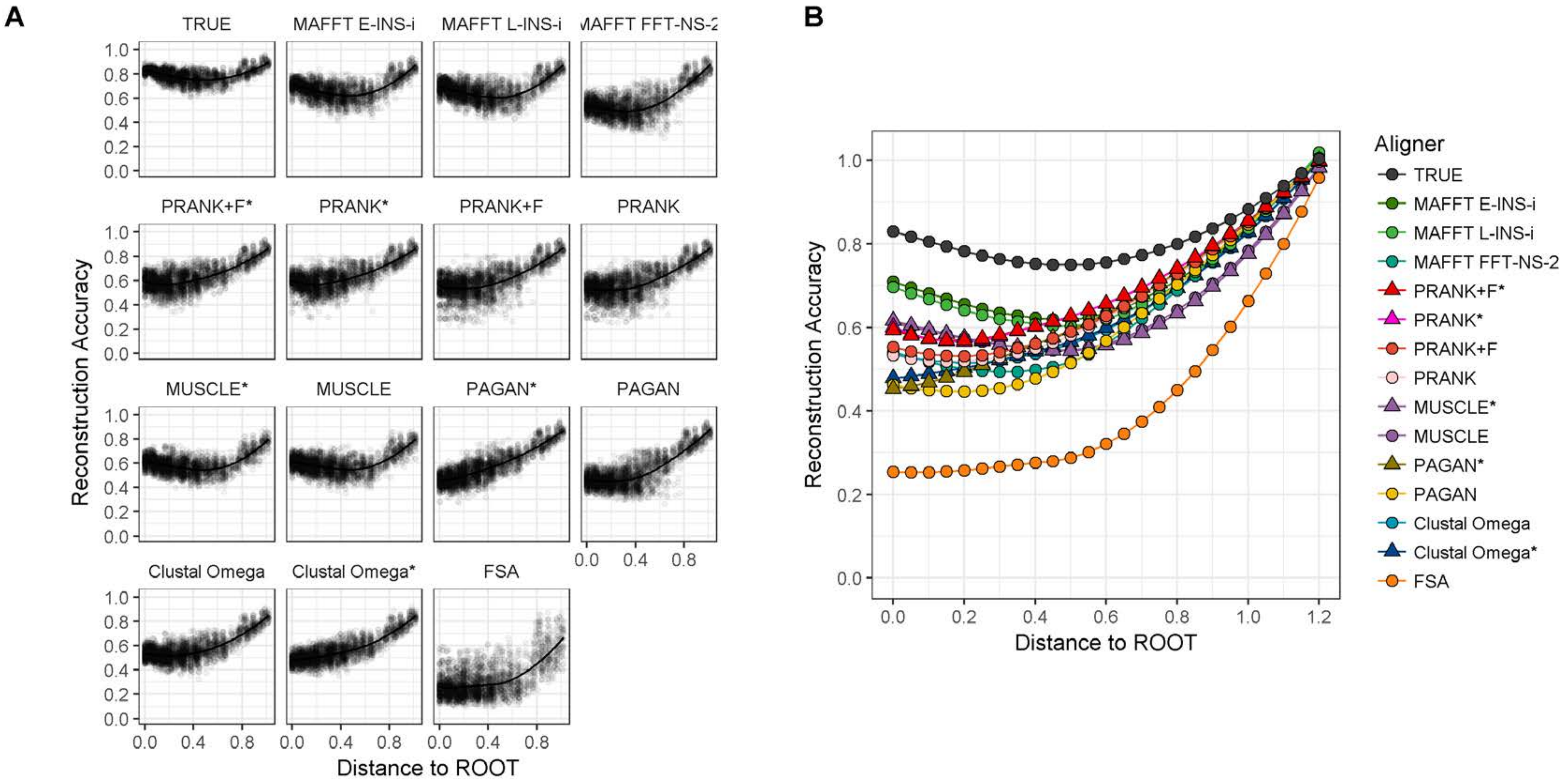

Taxa=32 Tree_height=1.2 Sampling_fraction=0.25 Indel_rate=0.01 Indel_model=POW 1.7 20

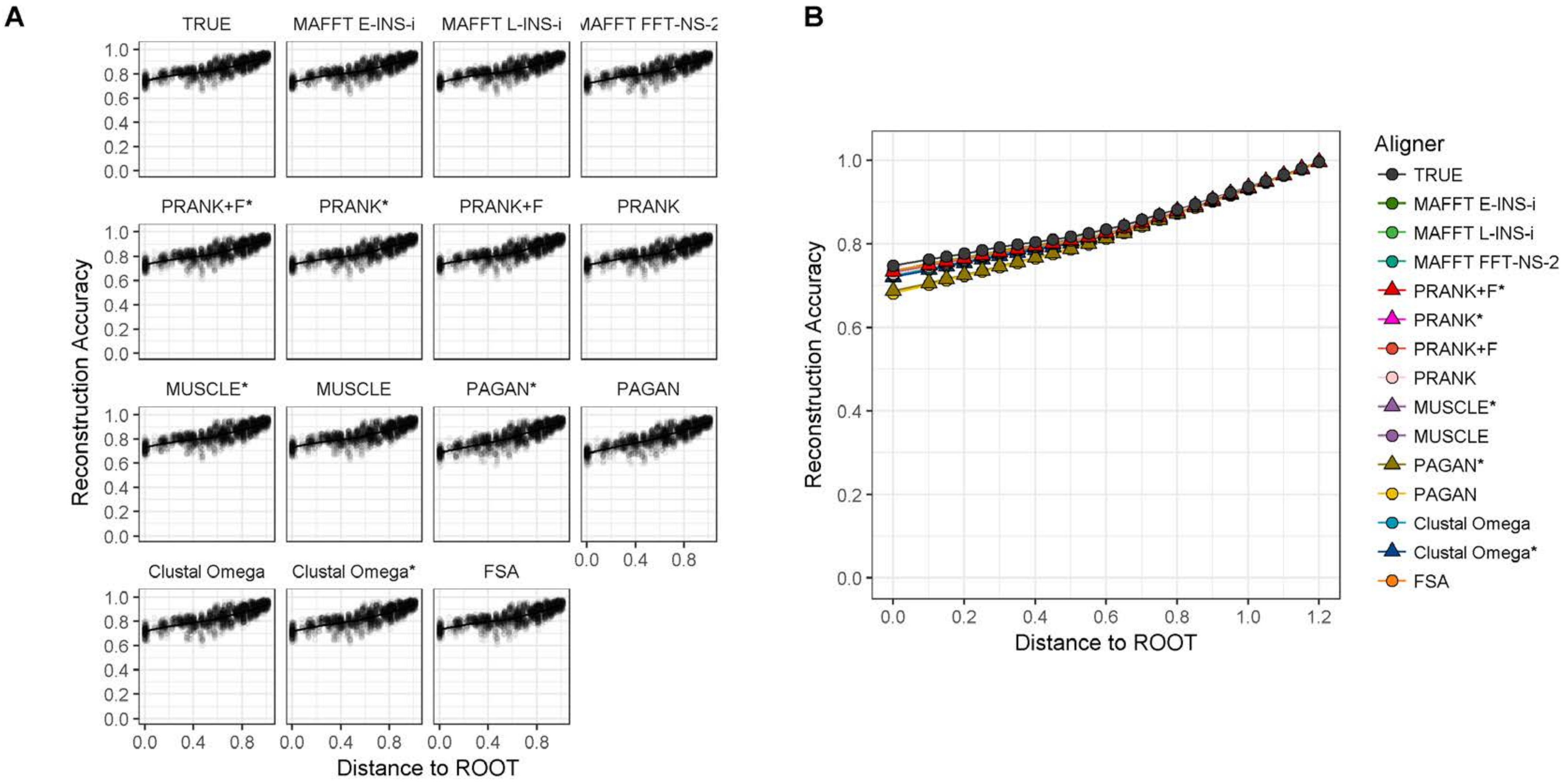

Taxa=32 Tree_height=1.2 Sampling_fraction=0.25 Indel_rate=0.05 Indel_model=POW 1.7 20

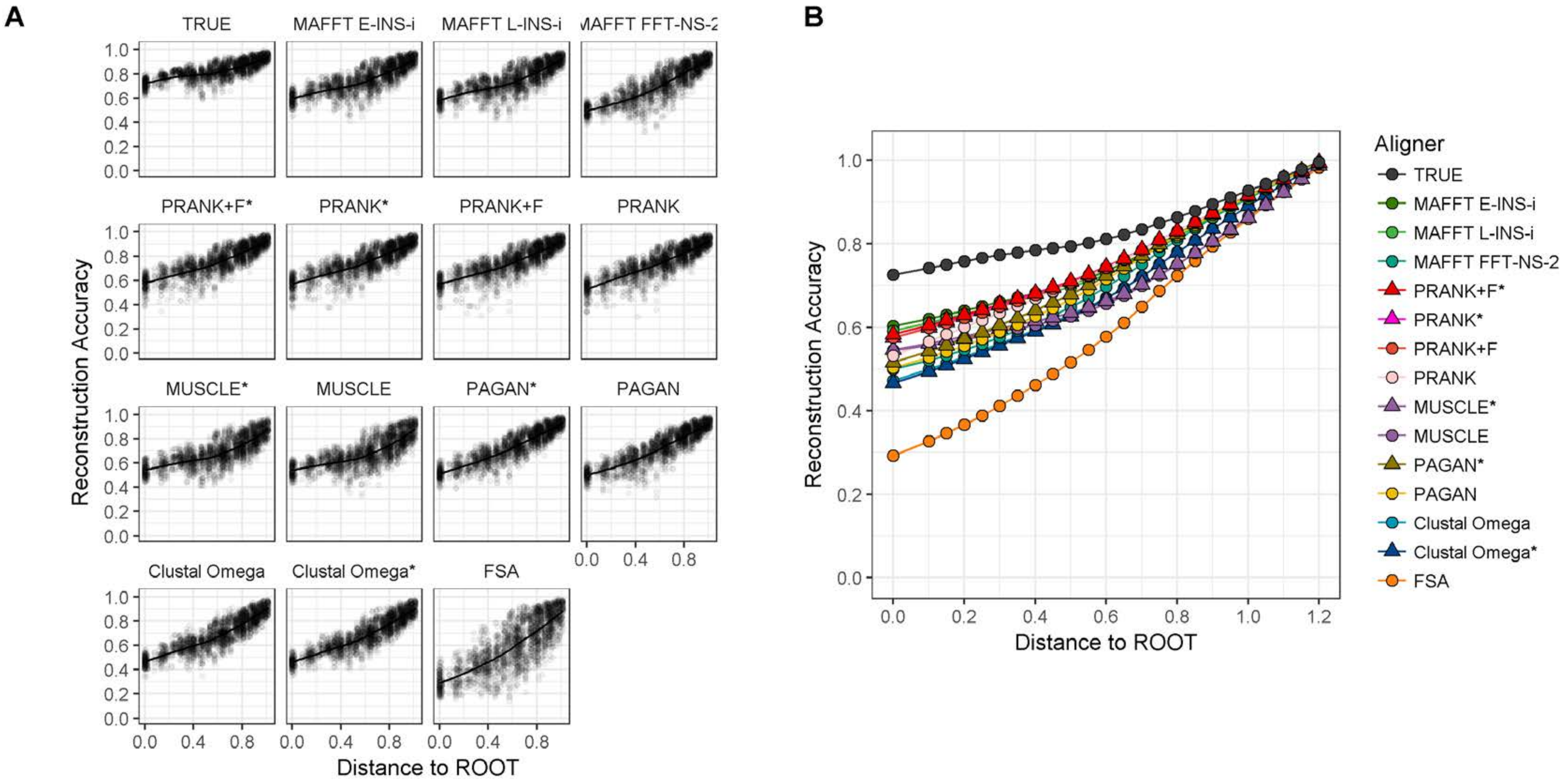

Taxa=32 Tree_height=1.2 Sampling_fraction=0.99 Indel_rate=0.01 Indel_model=POW 1.7 20

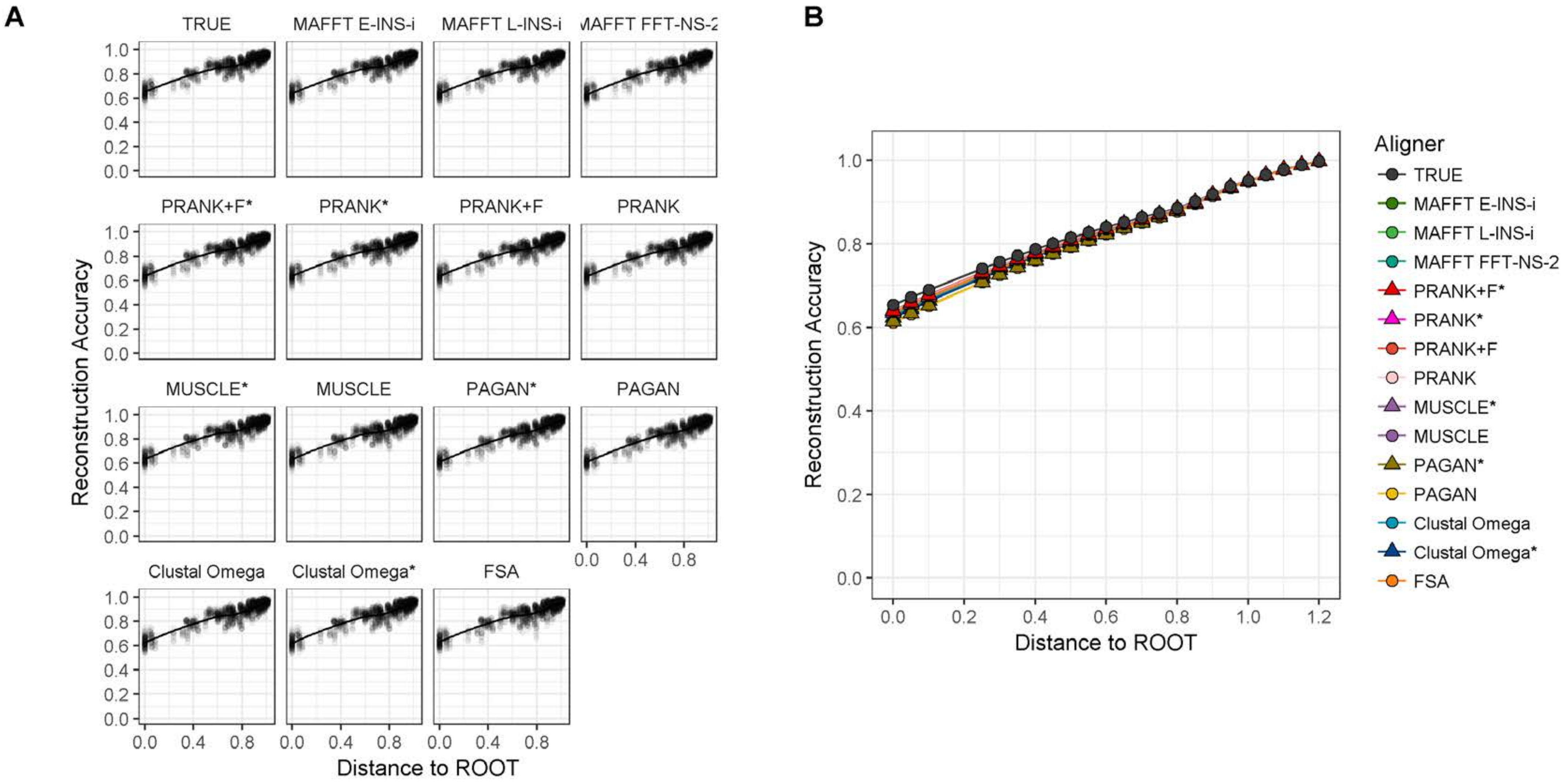

Taxa=32 Tree_height=1.2 Sampling_fraction=0.99 Indel_rate=0.05 Indel_model=POW 1.7 20

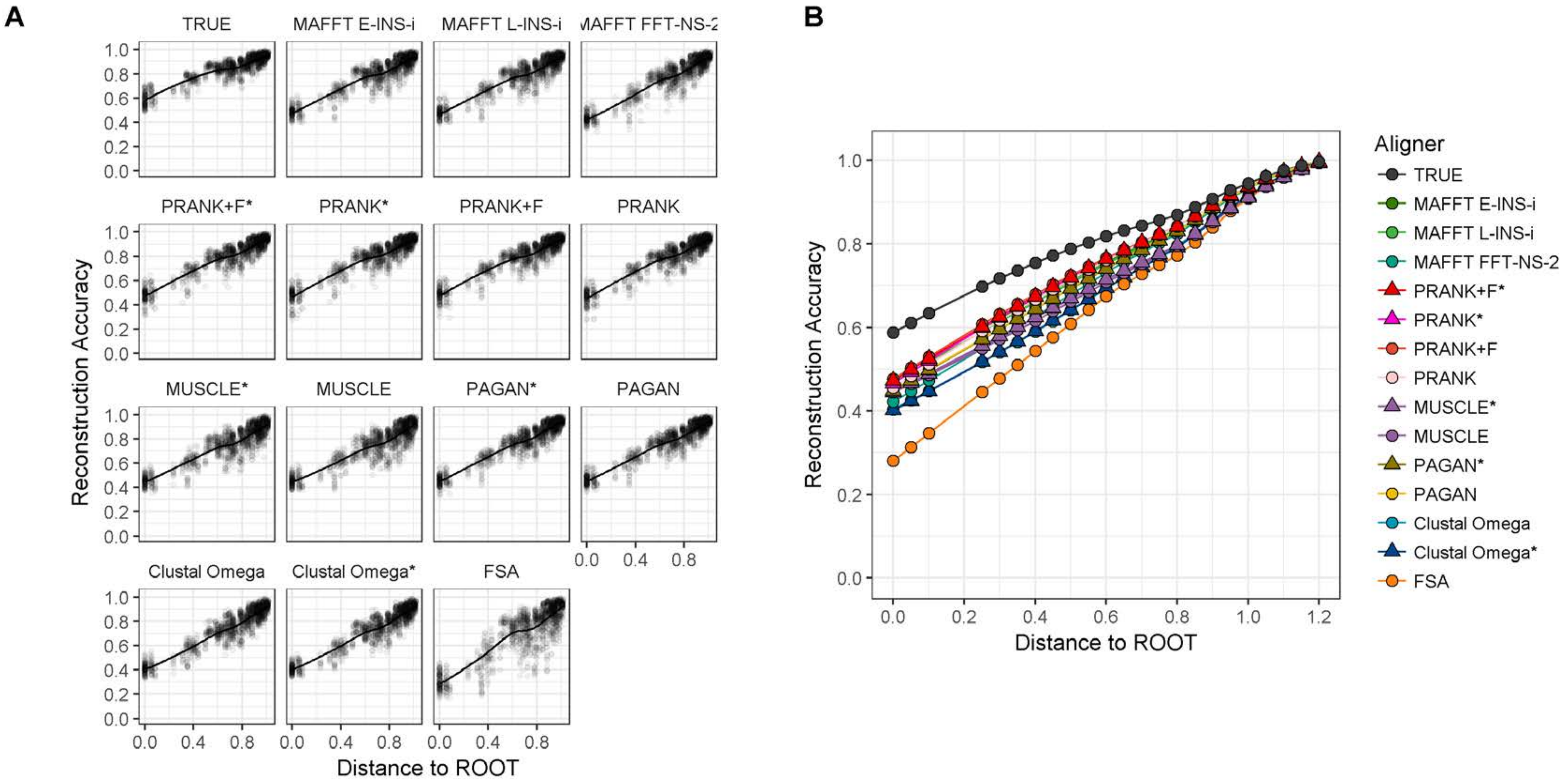

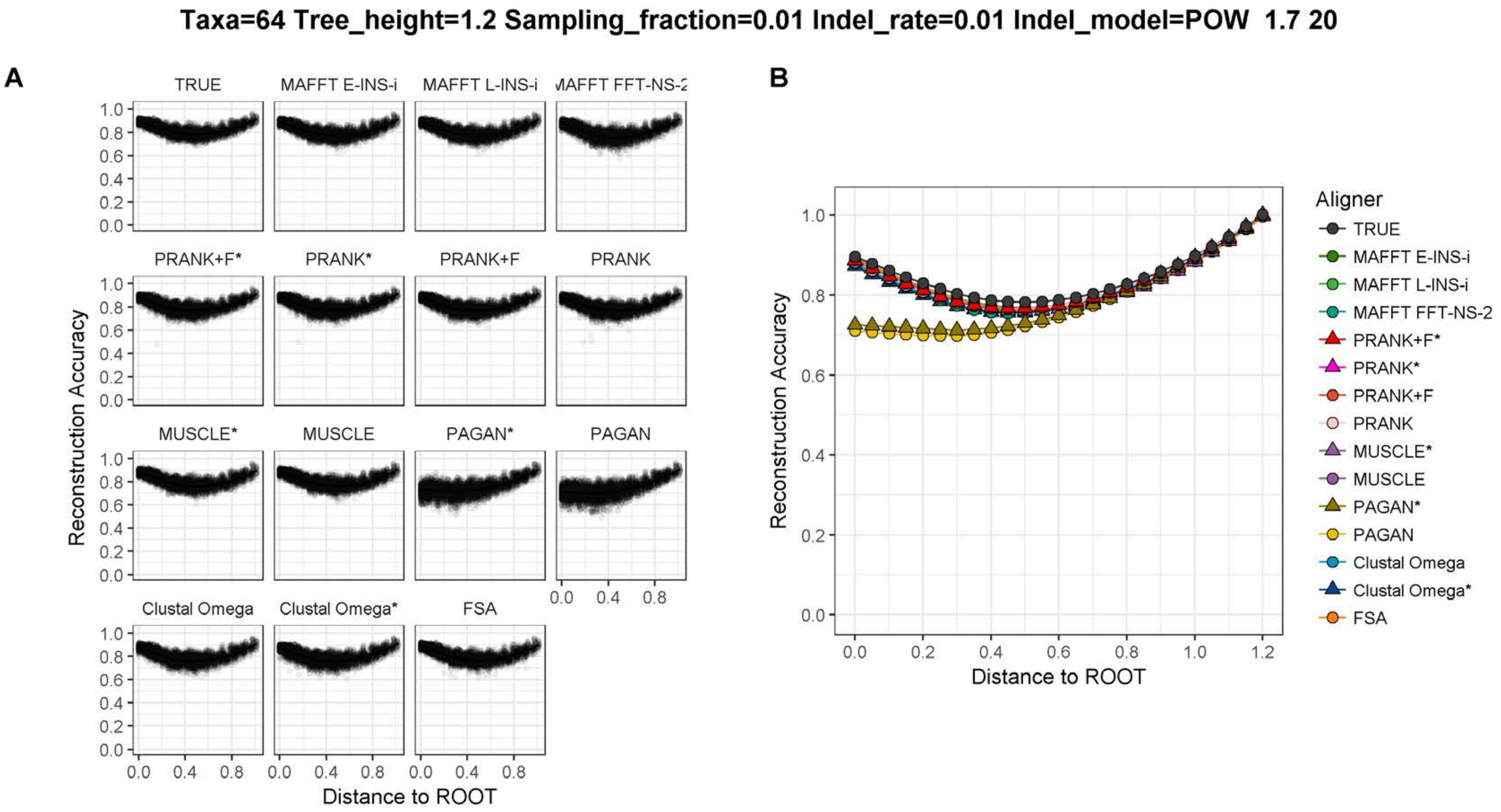
Taxa=64 Tree_height=1.2 Sampling_fraction=0.01 Indel_rate=0.01 Indel_model=POW 1.7 20
A
B
TRUE
MAFFT E-INS-i
MAFFT L-INS-i
MAFFT FFT-NS-2
PRANK+F*
PRANK*
PRANK+F
PRANK
MUSCLE*
MUSCLE
PAGAN*
PAGAN
Clustal Omega
Clustal Omega*
FSA
Reconstruction Accuracy
Distance to ROOT
Aligner

Taxa=64 Tree_height=1.2 Sampling_fraction=0.01 Indel_rate=0.05 Indel_model=POW 1.7 20

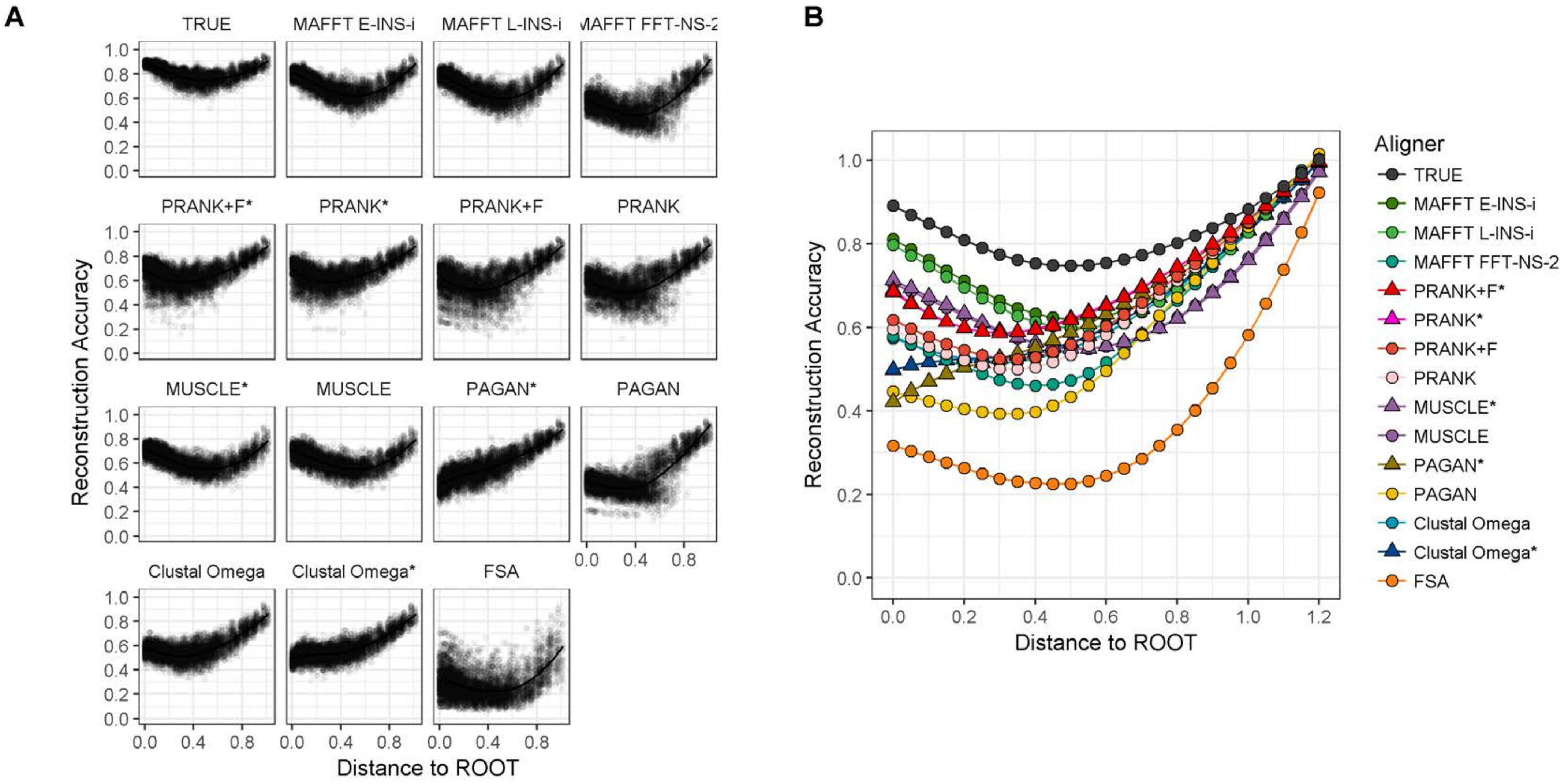

Taxa=64 Tree_height=1.2 Sampling_fraction=0.25 Indel_rate=0.01 Indel_model=POW 1.7 20

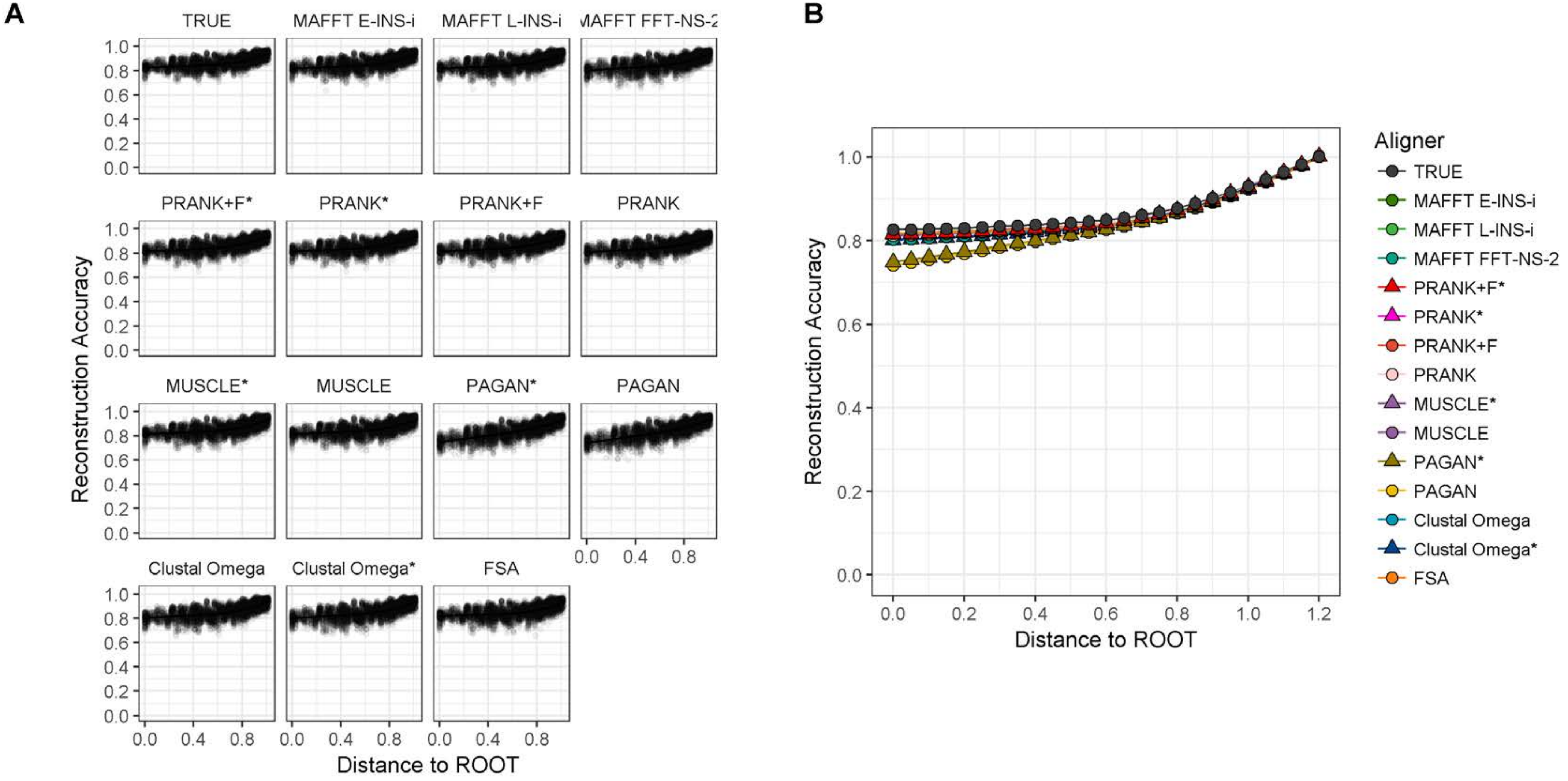

Taxa=64 Tree_height=1.2 Sampling_fraction=0.25 Indel_rate=0.05 Indel_model=POW 1.7 20

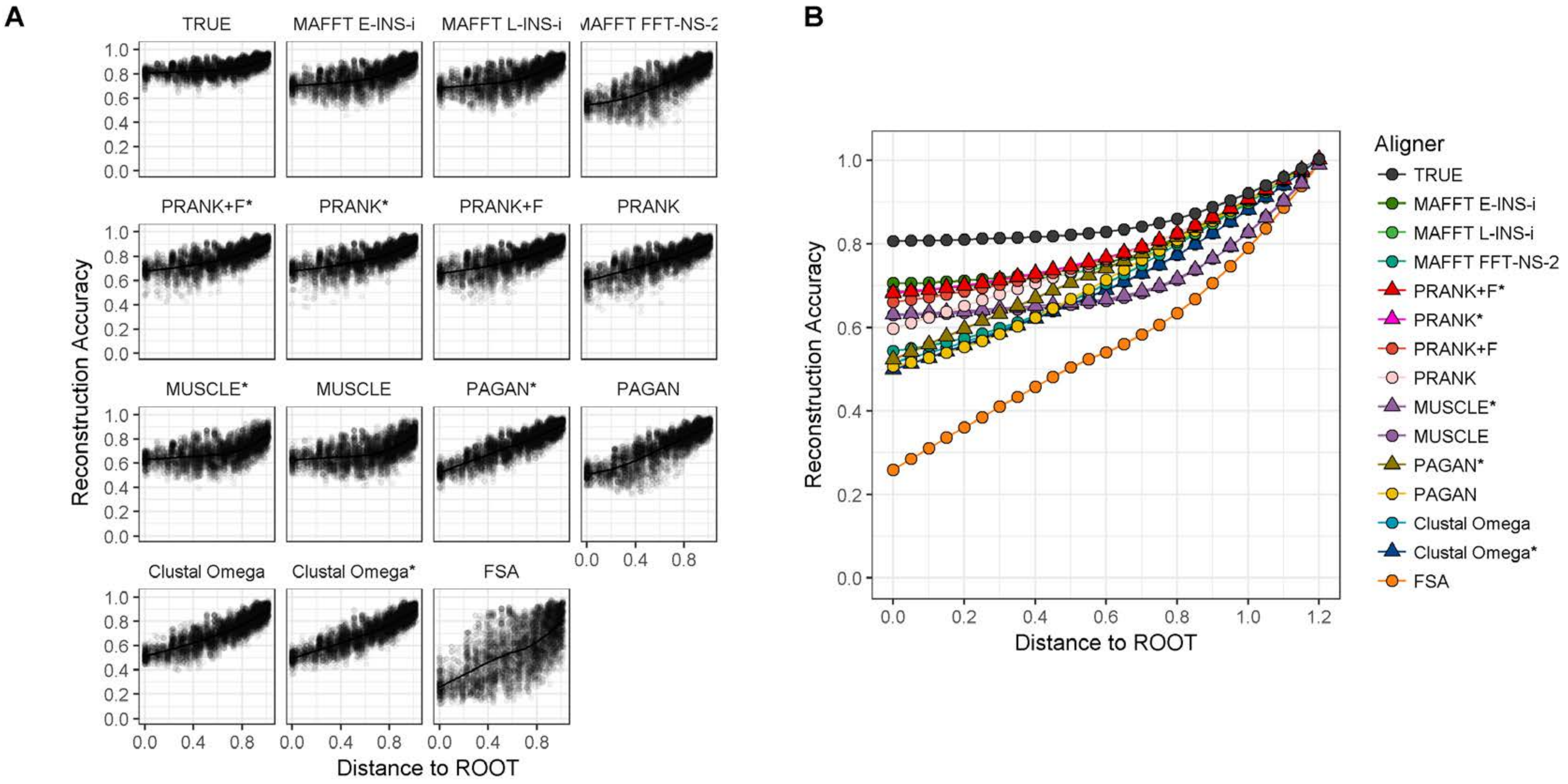

Taxa=64 Tree_height=1.2 Sampling_fraction=0.99 Indel_rate=0.01 Indel_model=POW 1.7 20

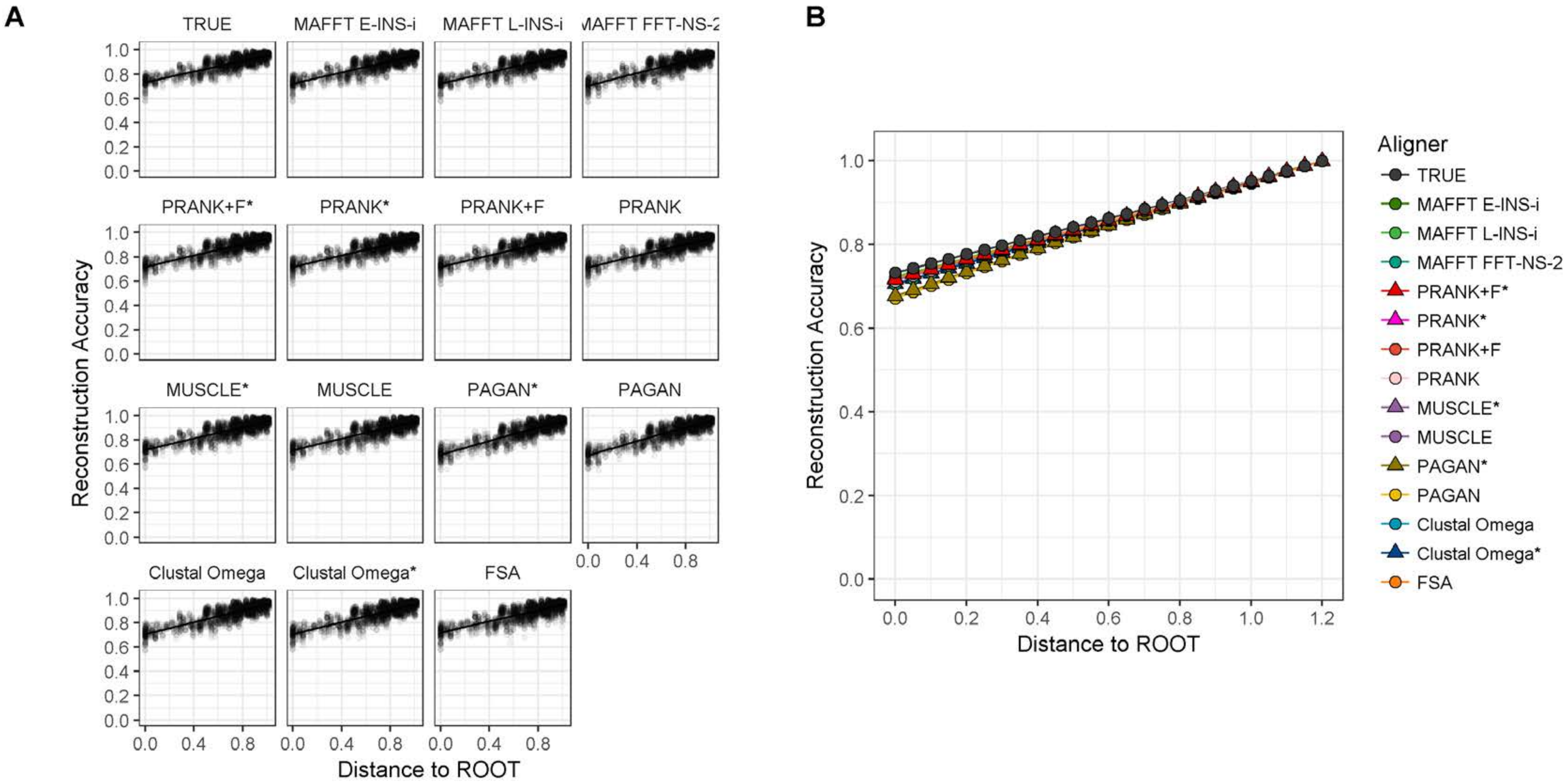

Taxa=64 Tree_height=1.2 Sampling_fraction=0.99 Indel_rate=0.05 Indel_model=POW 1.7 20

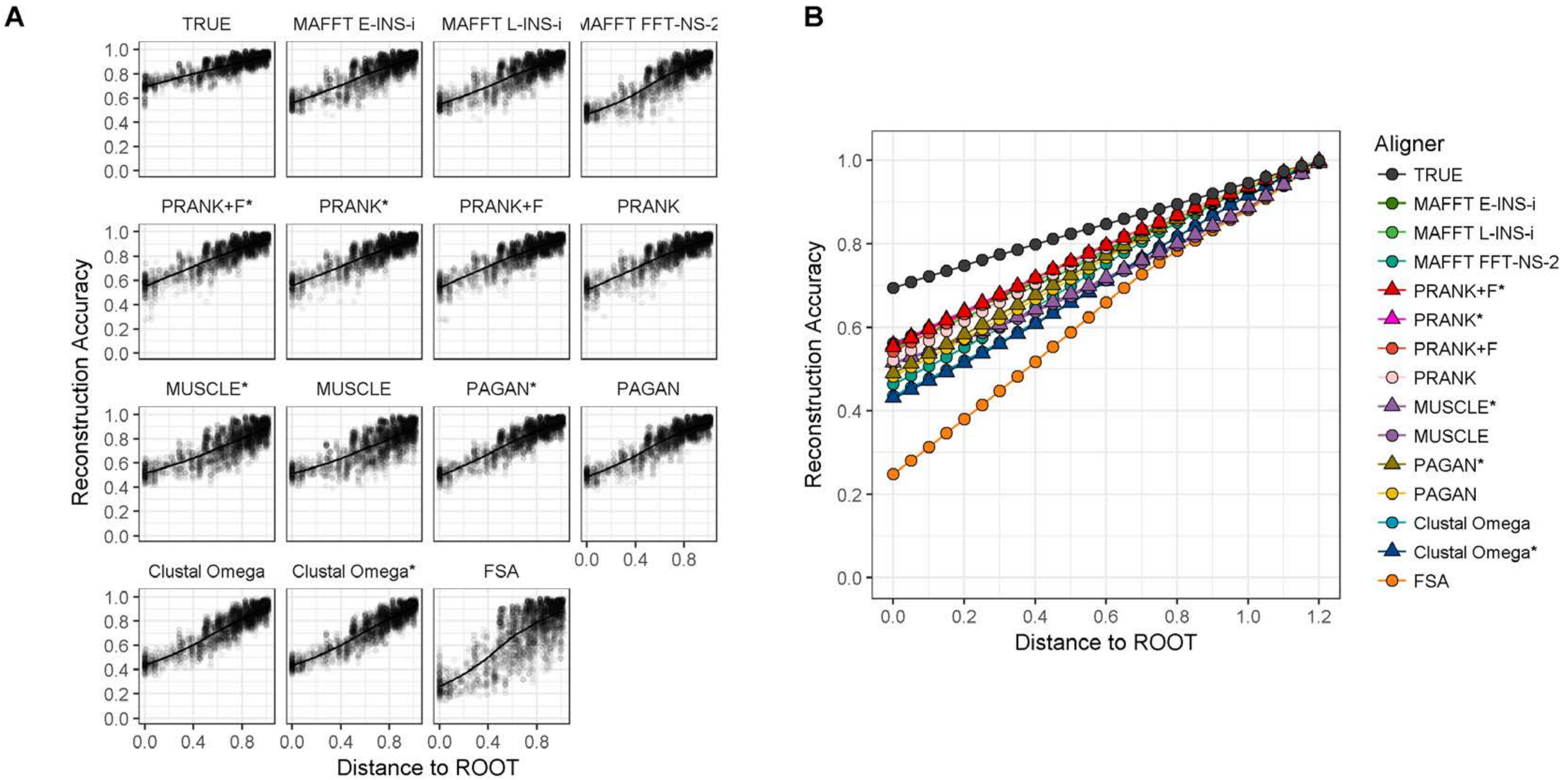

Taxa=16 Tree_height=2 Sampling_fraction=0.01 Indel_rate=0.01 Indel_model=POW 1.7 20

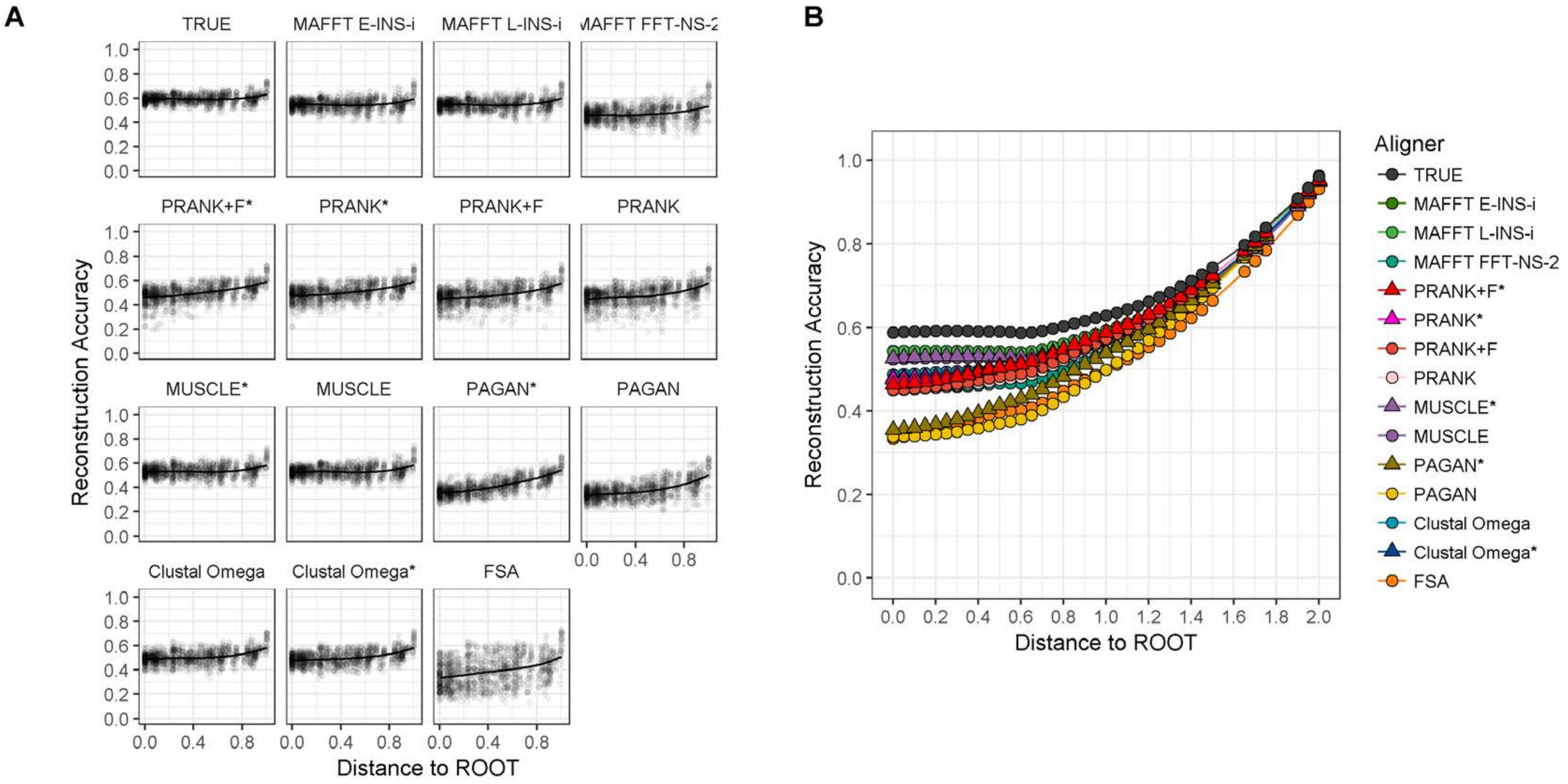

Taxa=16 Tree_height=2 Sampling_fraction=0.01 Indel_rate=0.05 Indel_model=POW 1.7 20

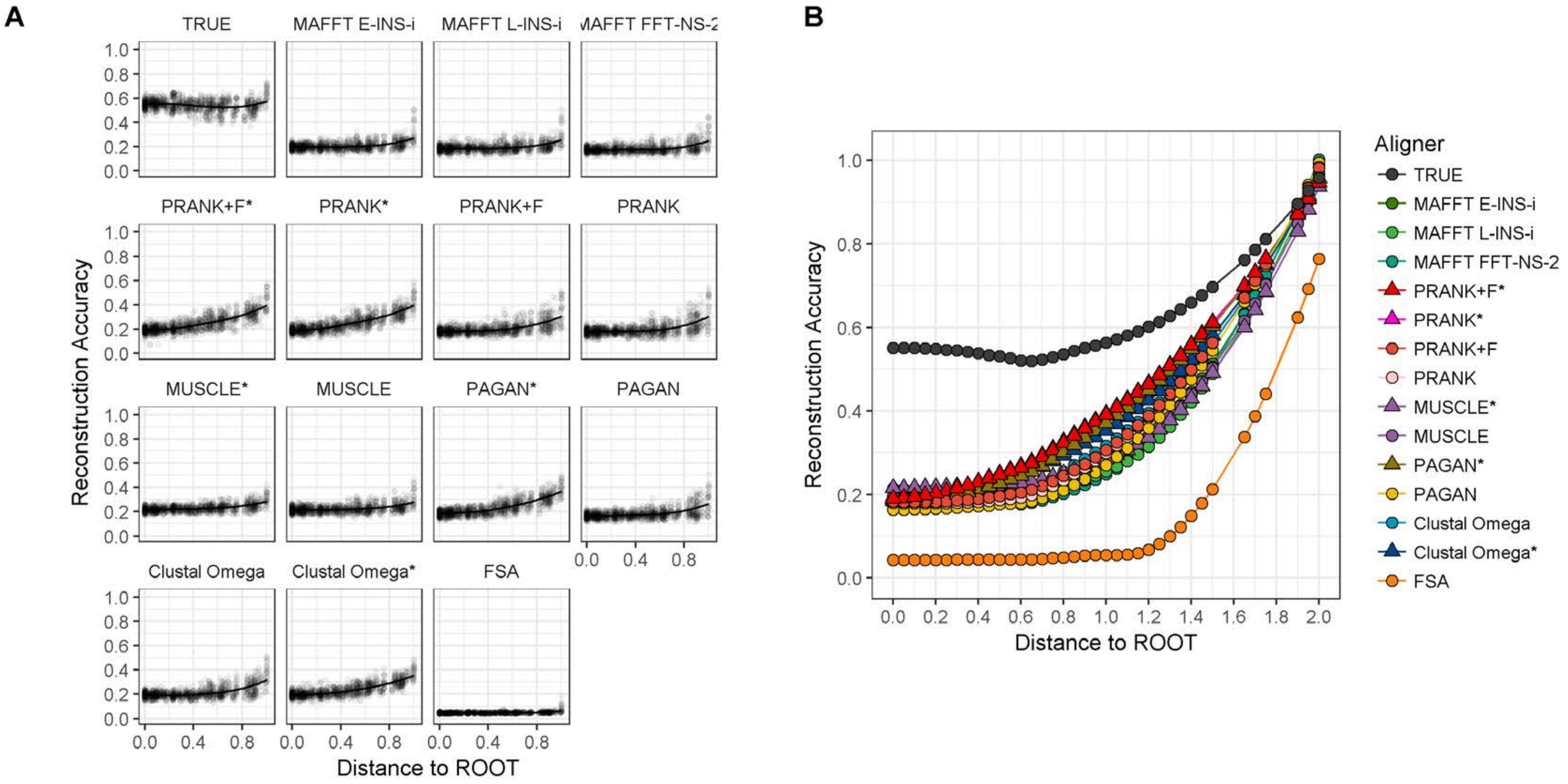

Taxa=16 Tree_height=2 Sampling_fraction=0.25 Indel_rate=0.01 Indel_model=POW 1.7 20

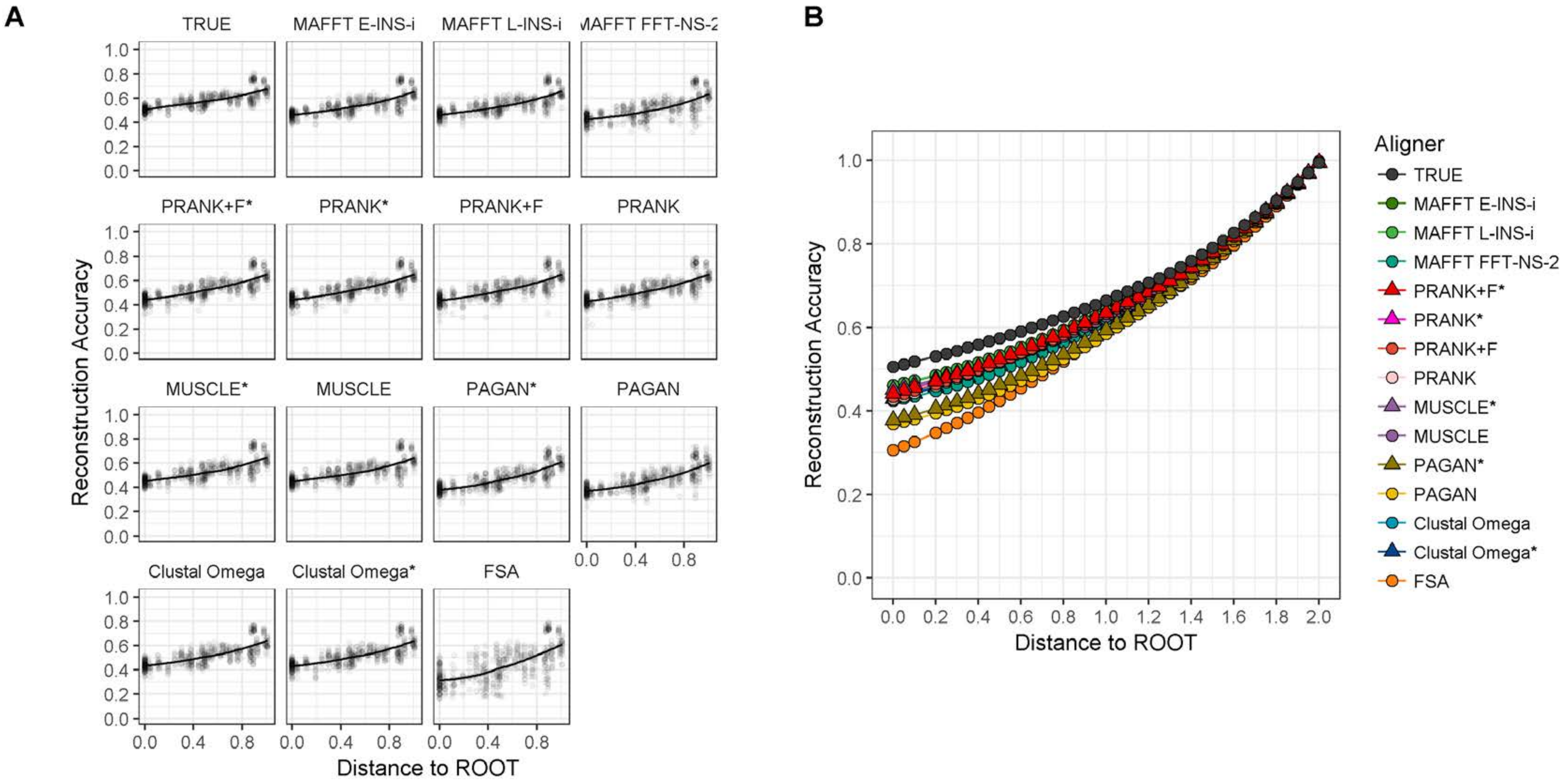

Taxa=16 Tree_height=2 Sampling_fraction=0.25 Indel_rate=0.05 Indel_model=POW 1.7 20

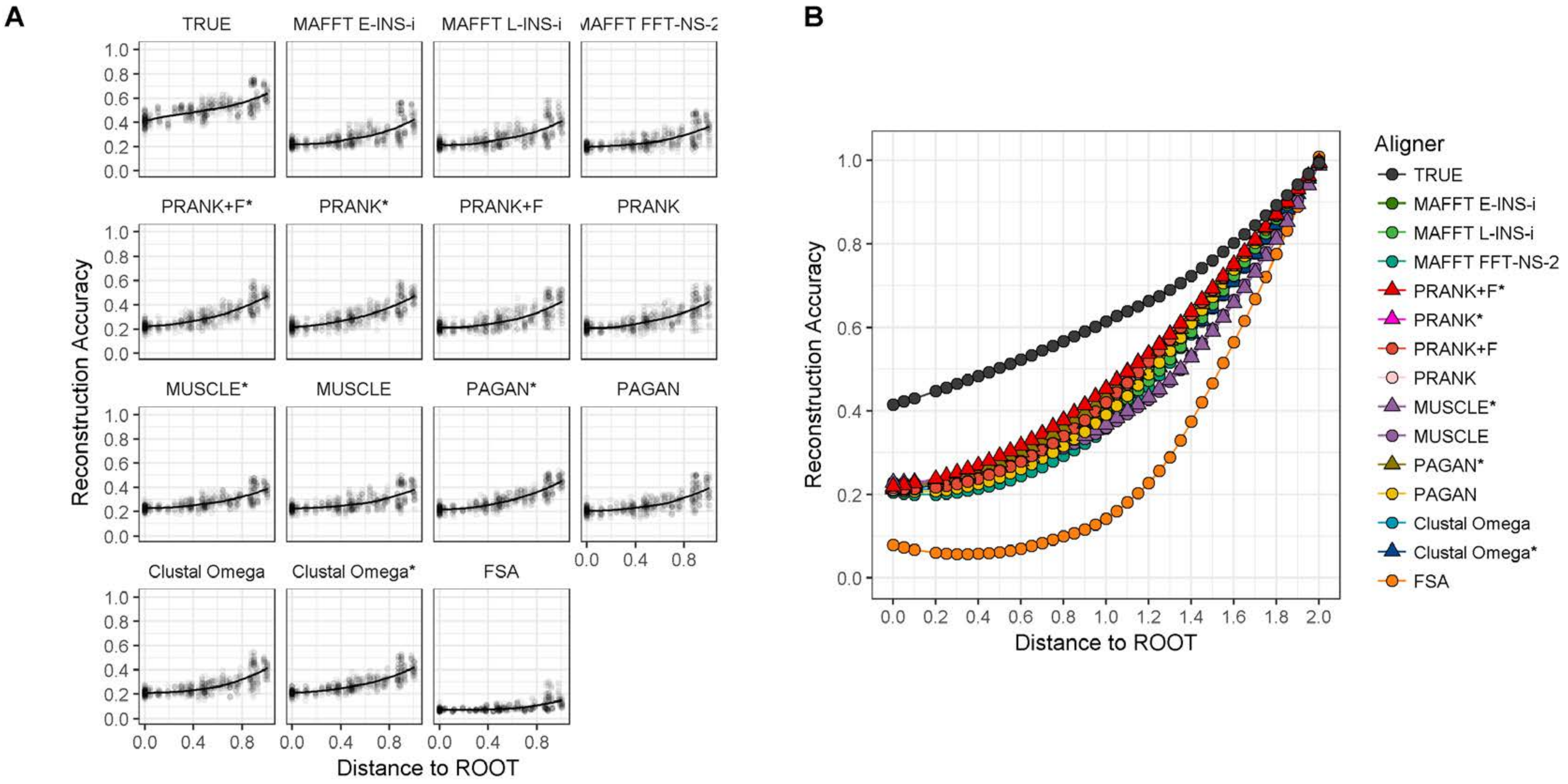

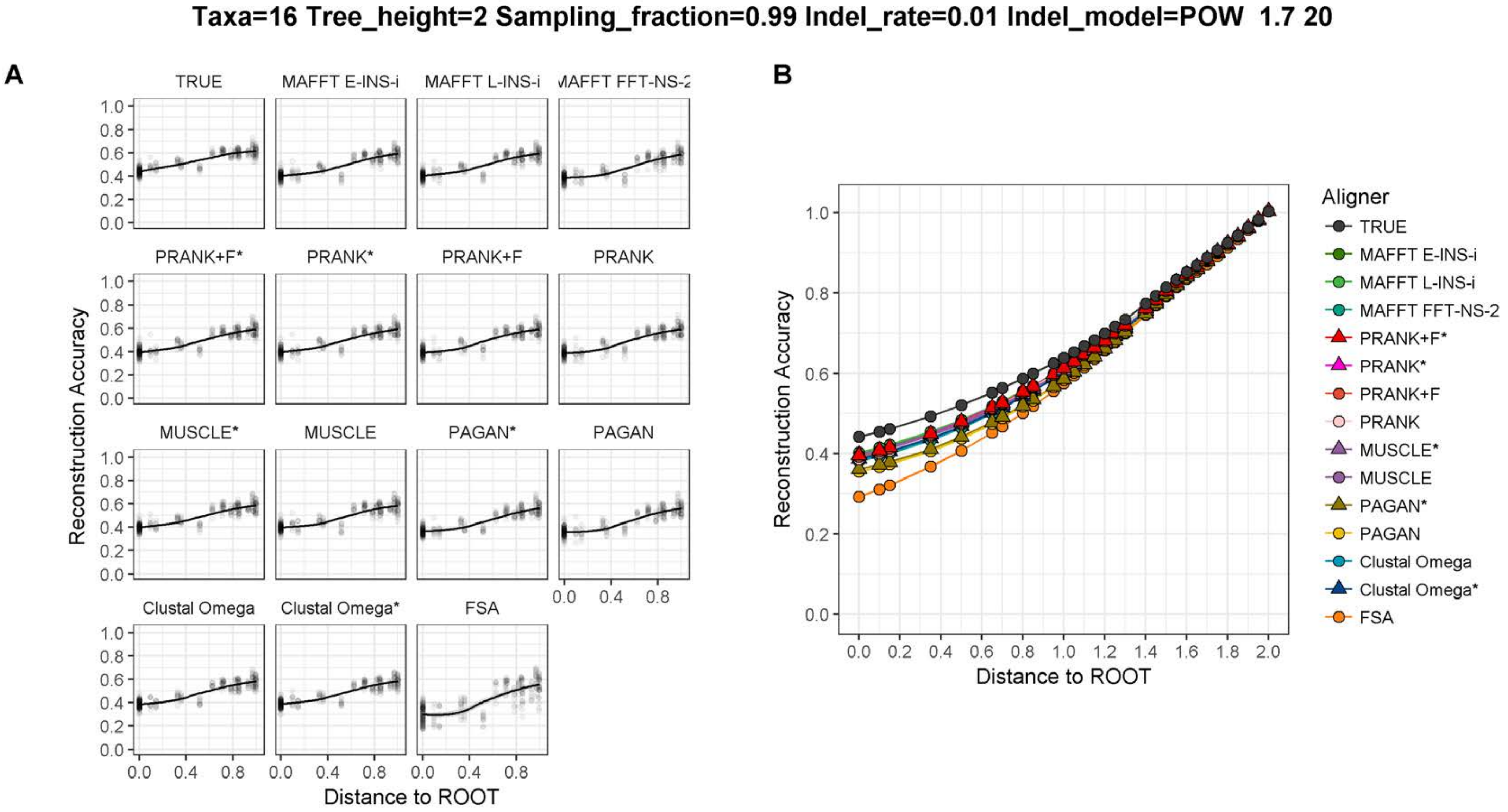
Taxa=16 Tree_height=2 Sampling_fraction=0.99 Indel_rate=0.01 Indel_model=POW 1.7 20
A
TRUE
MAFFT E-INS-i
MAFFT L-INS-i
PRANK+F*
PRANK*
PRANK+F
PRANK
MUSCLE*
MUSCLE
PAGAN*
PAGAN
Clustal Omega
Clustal Omega*
FSA
Reconstruction Accuracy
Distance to ROOT
B
Reconstruction Accuracy
Distance to ROOT
Aligner
TRUE
MAFFT E-INS-i
MAFFT L-INS-i
MAFFT FFT-NS-2
PRANK+F*
PRANK*
PRANK+F
PRANK
MUSCLE*
MUSCLE
PAGAN*
PAGAN
Clustal Omega
Clustal Omega*
FSA

Taxa=16 Tree_height=2 Sampling_fraction=0.99 Indel_rate=0.05 Indel_model=POW 1.7 20

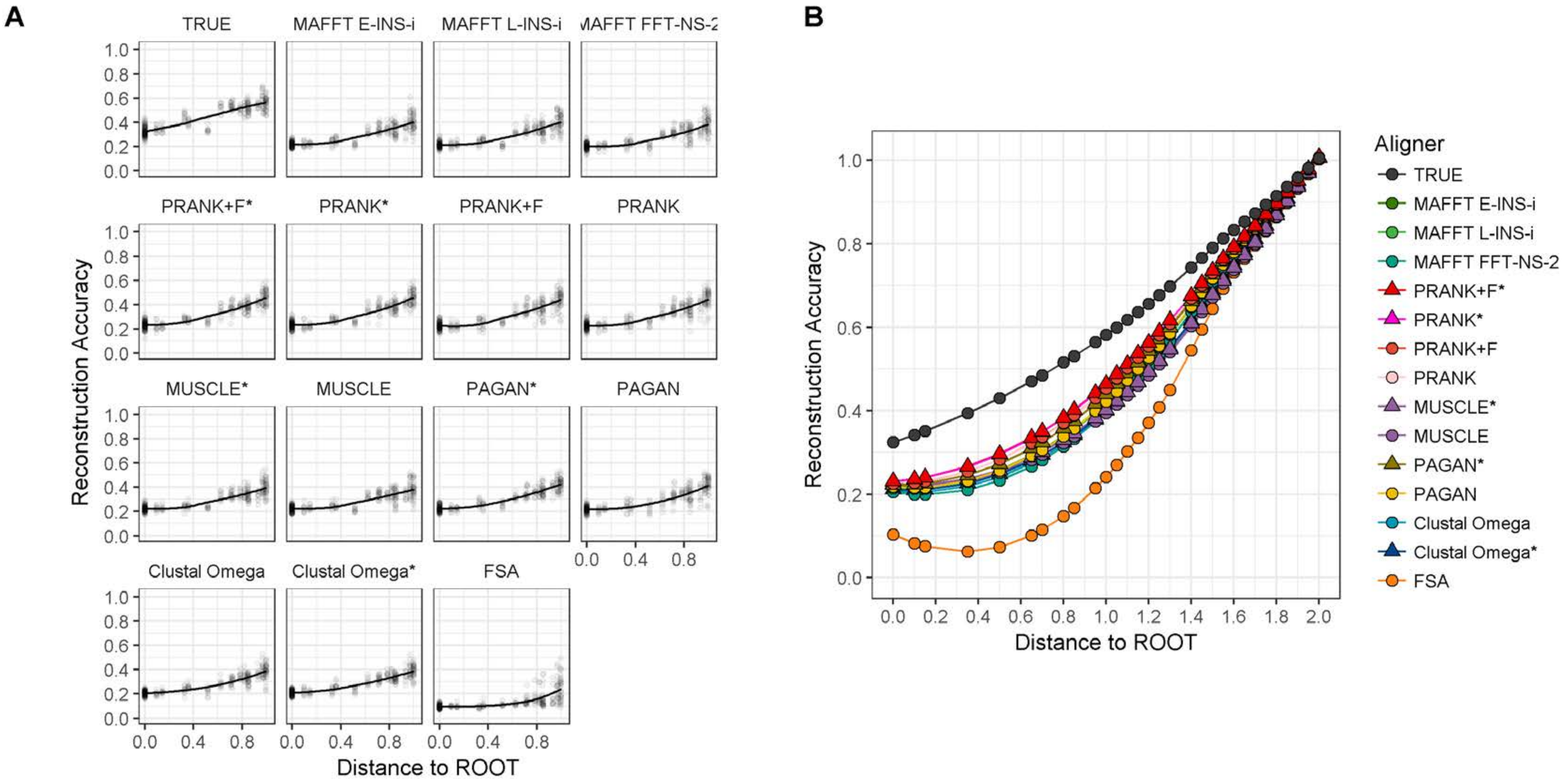

Taxa=32 Tree_height=2 Sampling_fraction=0.01 Indel_rate=0.01 Indel_model=POW 1.7 20

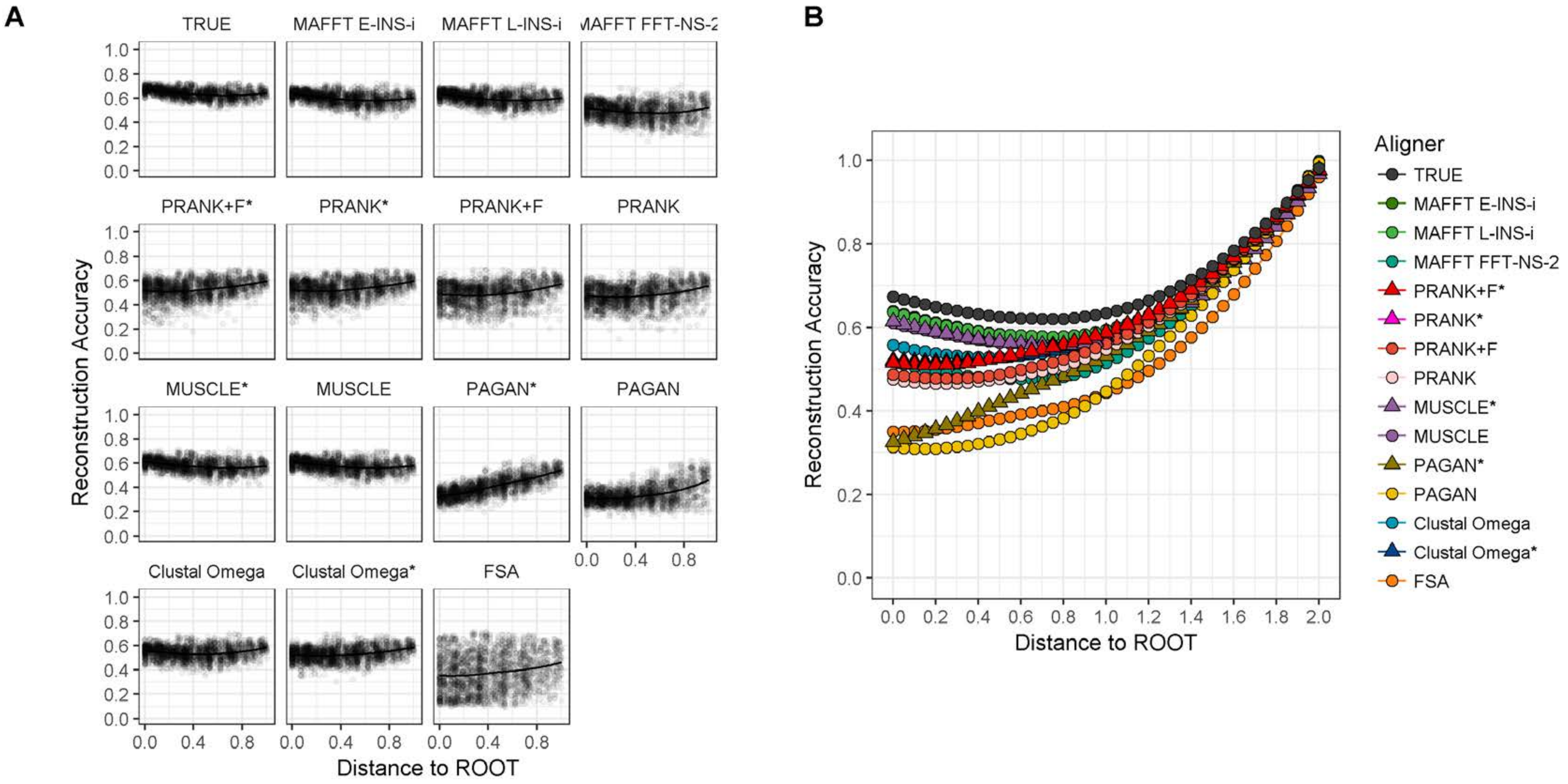

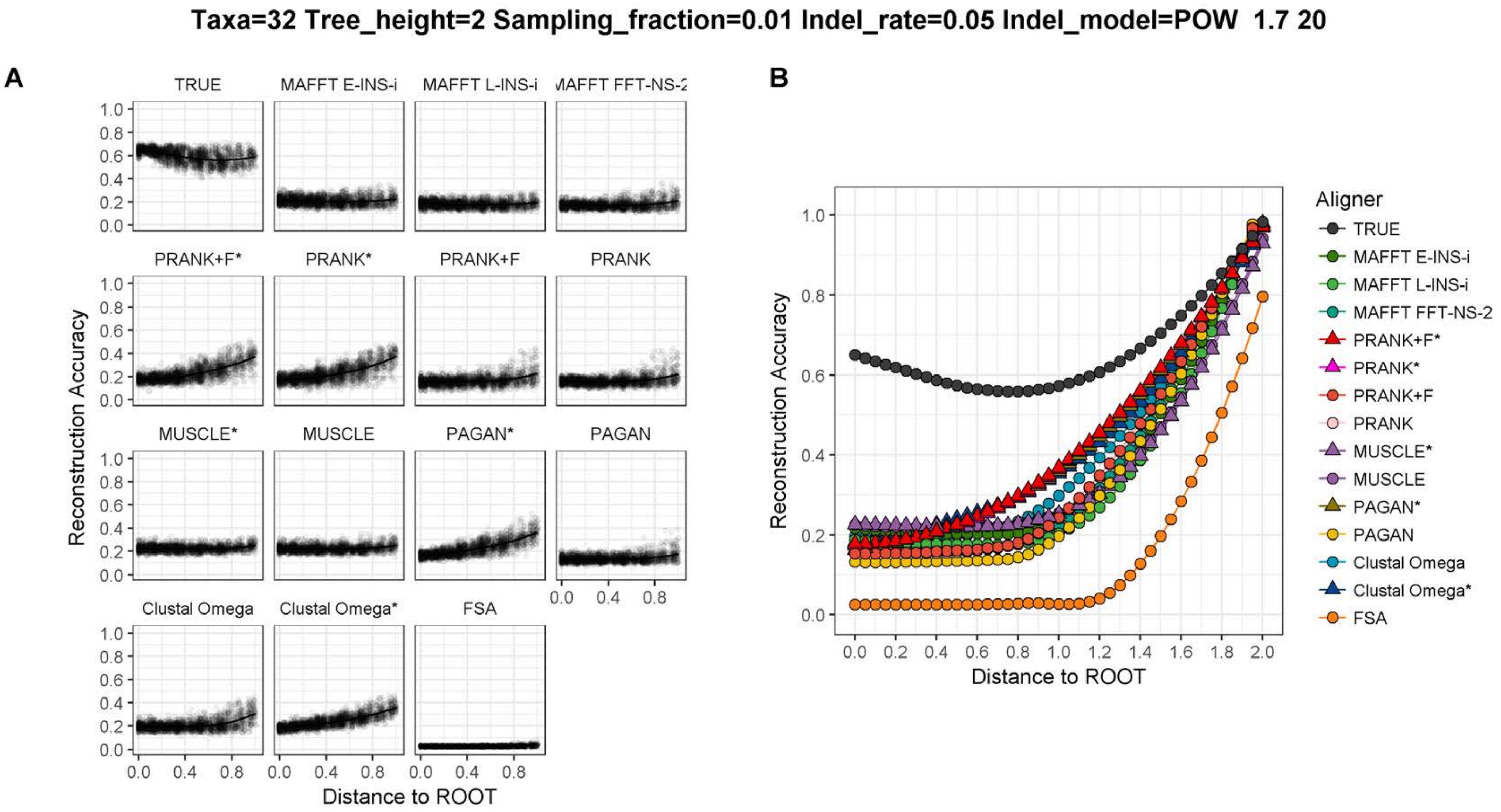
Taxa=32 Tree_height=2 Sampling_fraction=0.01 Indel_rate=0.05 Indel_model=POW 1.7 20
A
B
TRUE
MAFFT E-INS-i
MAFFT L-INS-i
PRANK+F*
PRANK*
PRANK+F
PRANK
MUSCLE*
MUSCLE
PAGAN*
PAGAN
Clustal Omega
Clustal Omega*
FSA
Reconstruction Accuracy
Distance to ROOT
Aligner
MAFFT FFT-NS-2

Taxa=32 Tree_height=2 Sampling_fraction=0.25 Indel_rate=0.01 Indel_model=POW 1.7 20

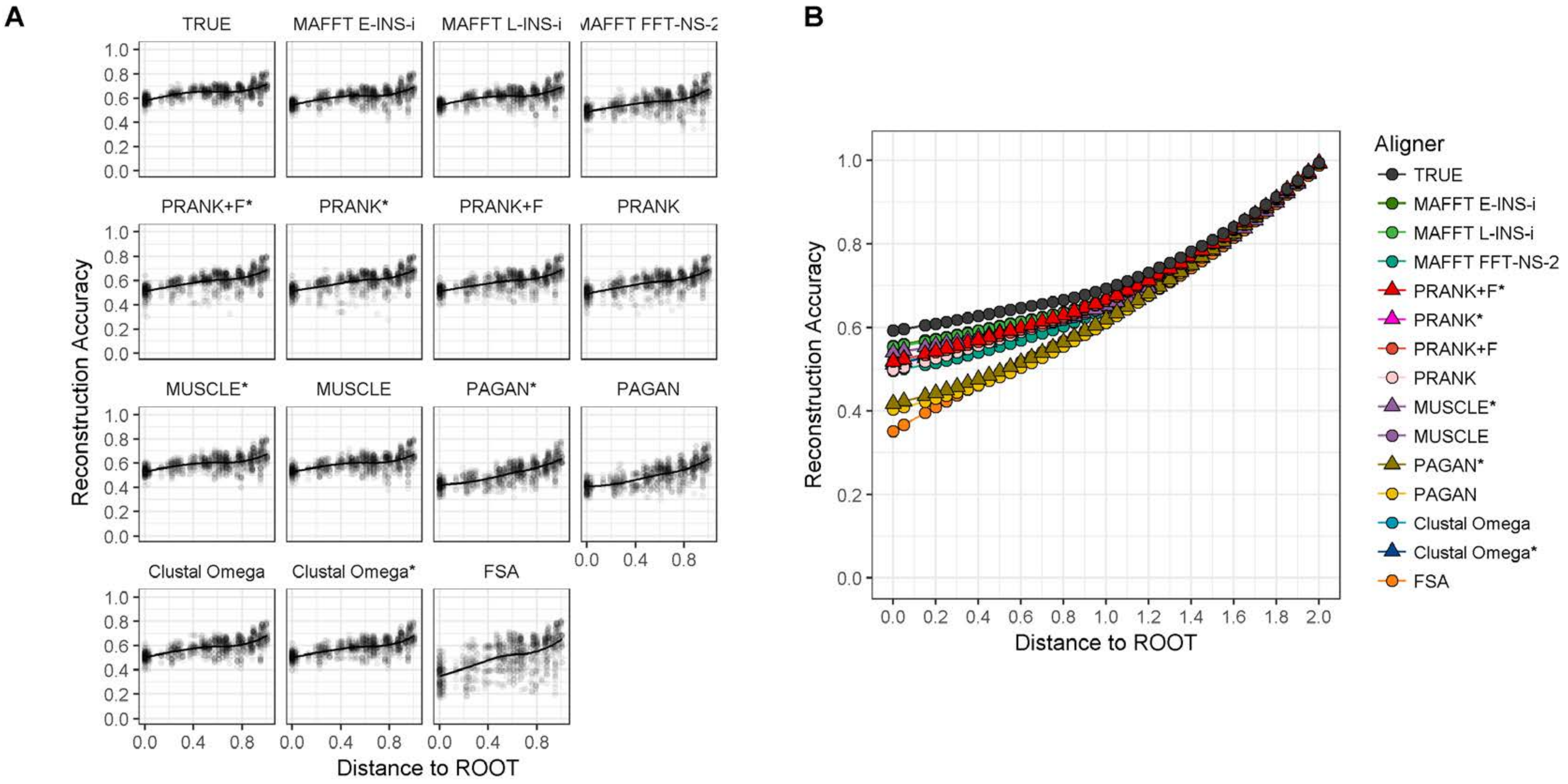

Taxa=32 Tree_height=2 Sampling_fraction=0.25 Indel_rate=0.05 Indel_model=POW 1.7 20

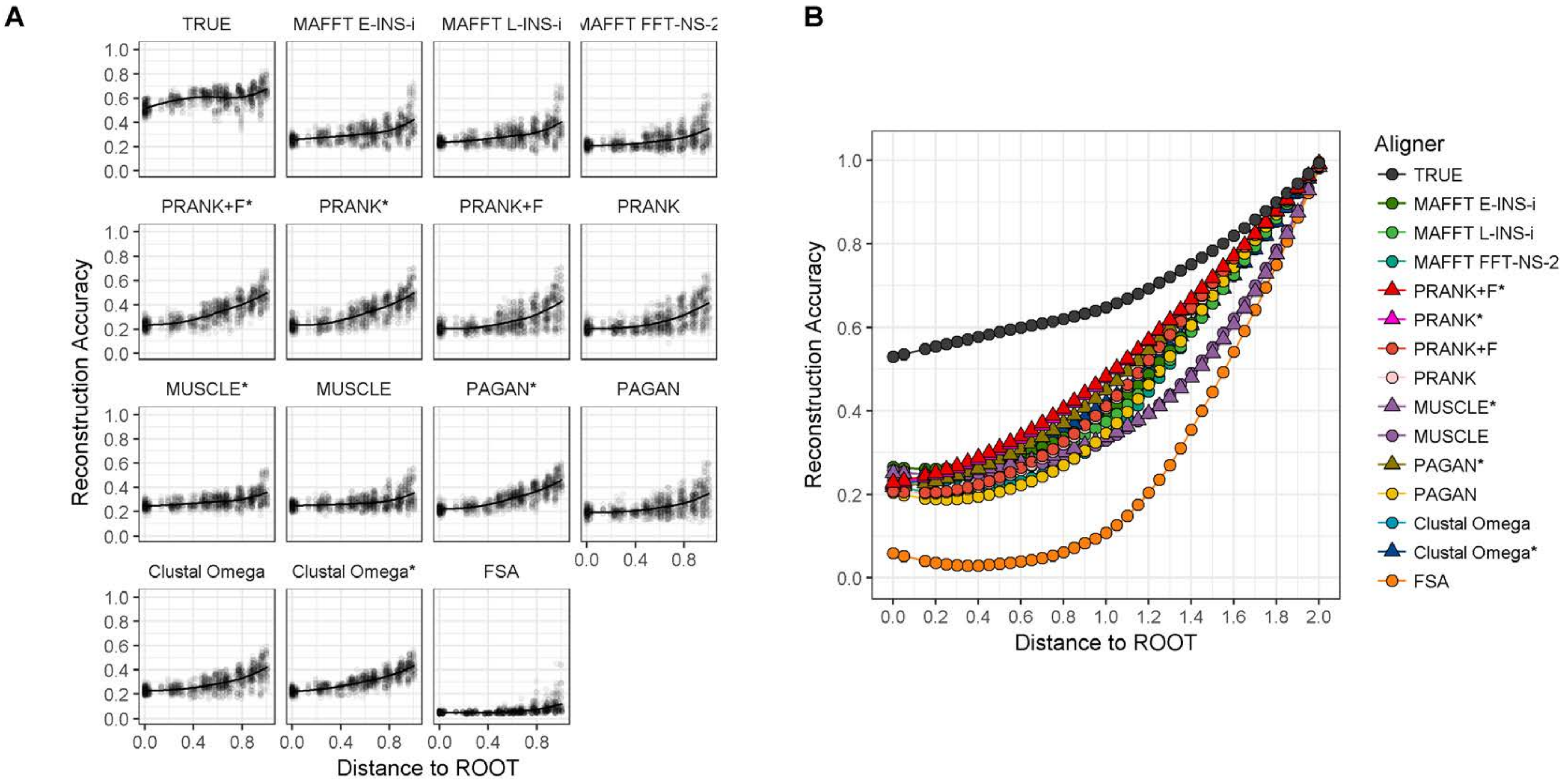

Taxa=32 Tree_height=2 Sampling_fraction=0.99 Indel_rate=0.01 Indel_model=POW 1.7 20

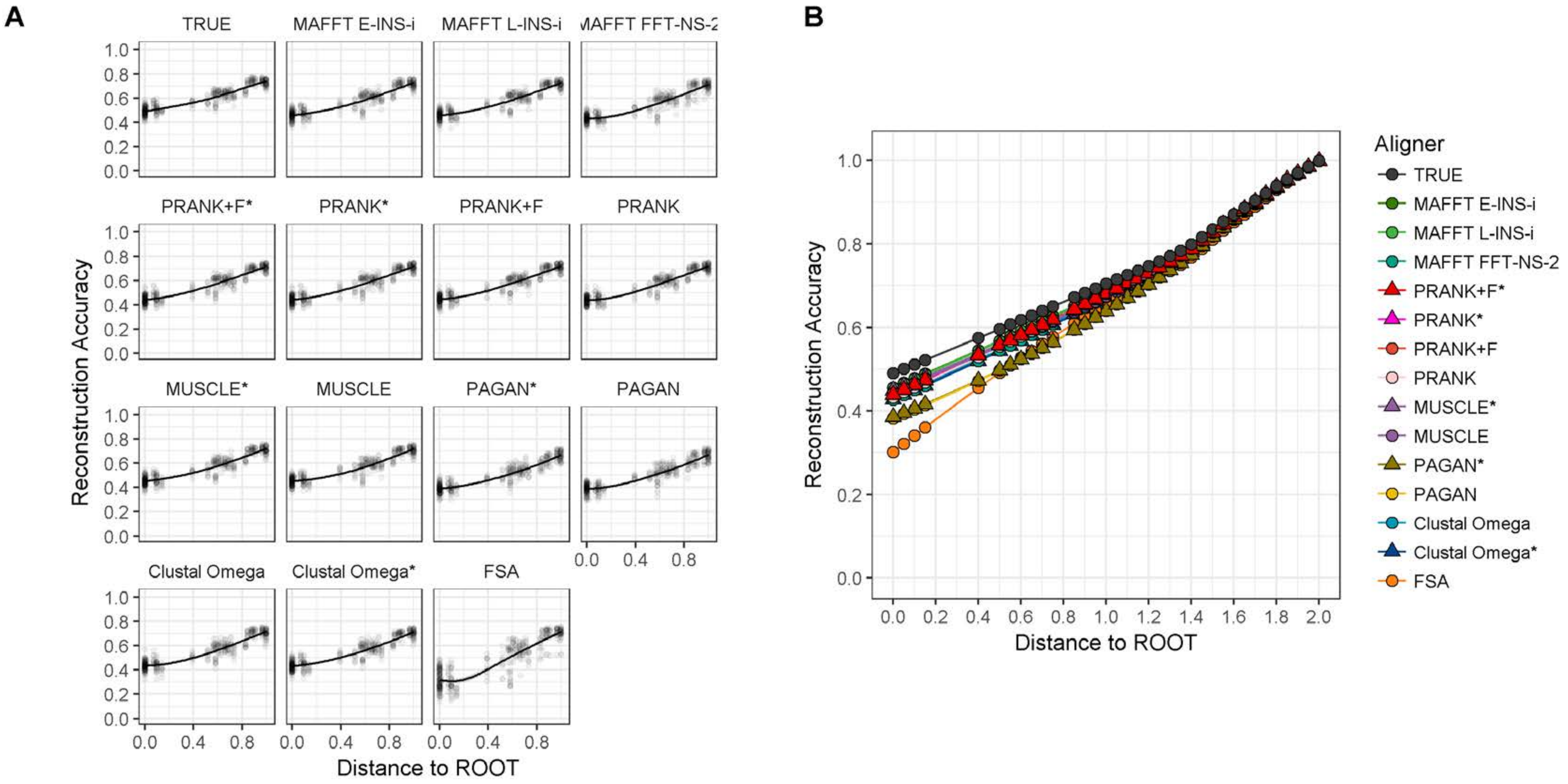

Taxa=32 Tree_height=2 Sampling_fraction=0.99 Indel_rate=0.05 Indel_model=POW 1.7 20

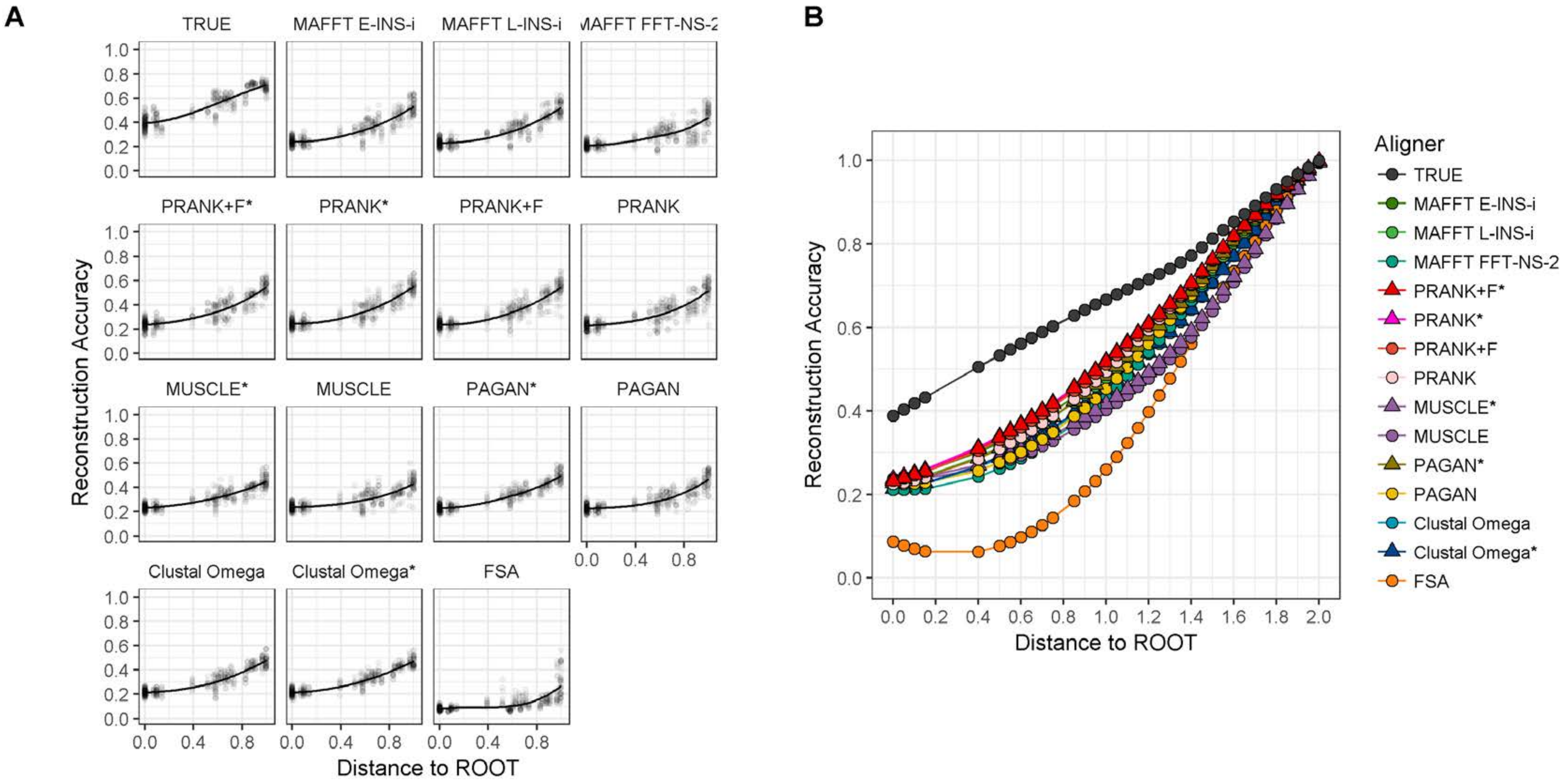

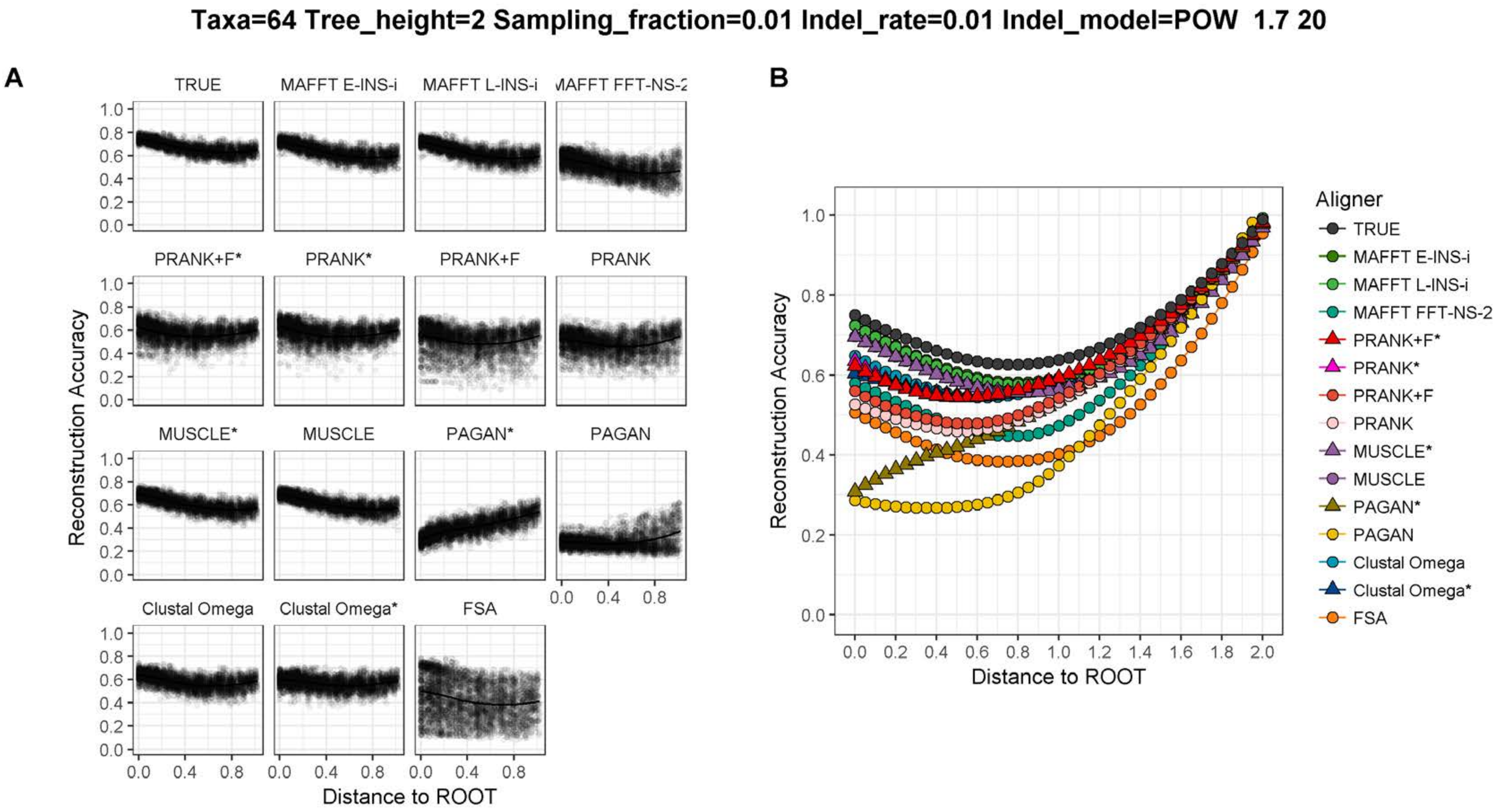

Taxa=64 Tree_height=2 Sampling_fraction=0.01 Indel_rate=0.01 Indel_model=POW 1.7 20
A
TRUE
MAFFT E-INS-i
MAFFT L-INS-i
MAFFT FFT-NS-2
PRANK+F*
PRANK*
PRANK+F
PRANK
MUSCLE*
MUSCLE
PAGAN*
PAGAN
Clustal Omega
Clustal Omega*
FSA
Reconstruction Accuracy
Distance to ROOT
B
Aligner
TRUE
MAFFT E-INS-i
MAFFT L-INS-i
MAFFT FFT-NS-2
PRANK+F*
PRANK*
PRANK+F
PRANK
MUSCLE*
MUSCLE
PAGAN*
PAGAN
Clustal Omega
Clustal Omega*
FSA
Reconstruction Accuracy
Distance to ROOT

Taxa=64 Tree_height=2 Sampling_fraction=0.01 Indel_rate=0.05 Indel_model=POW 1.7 20

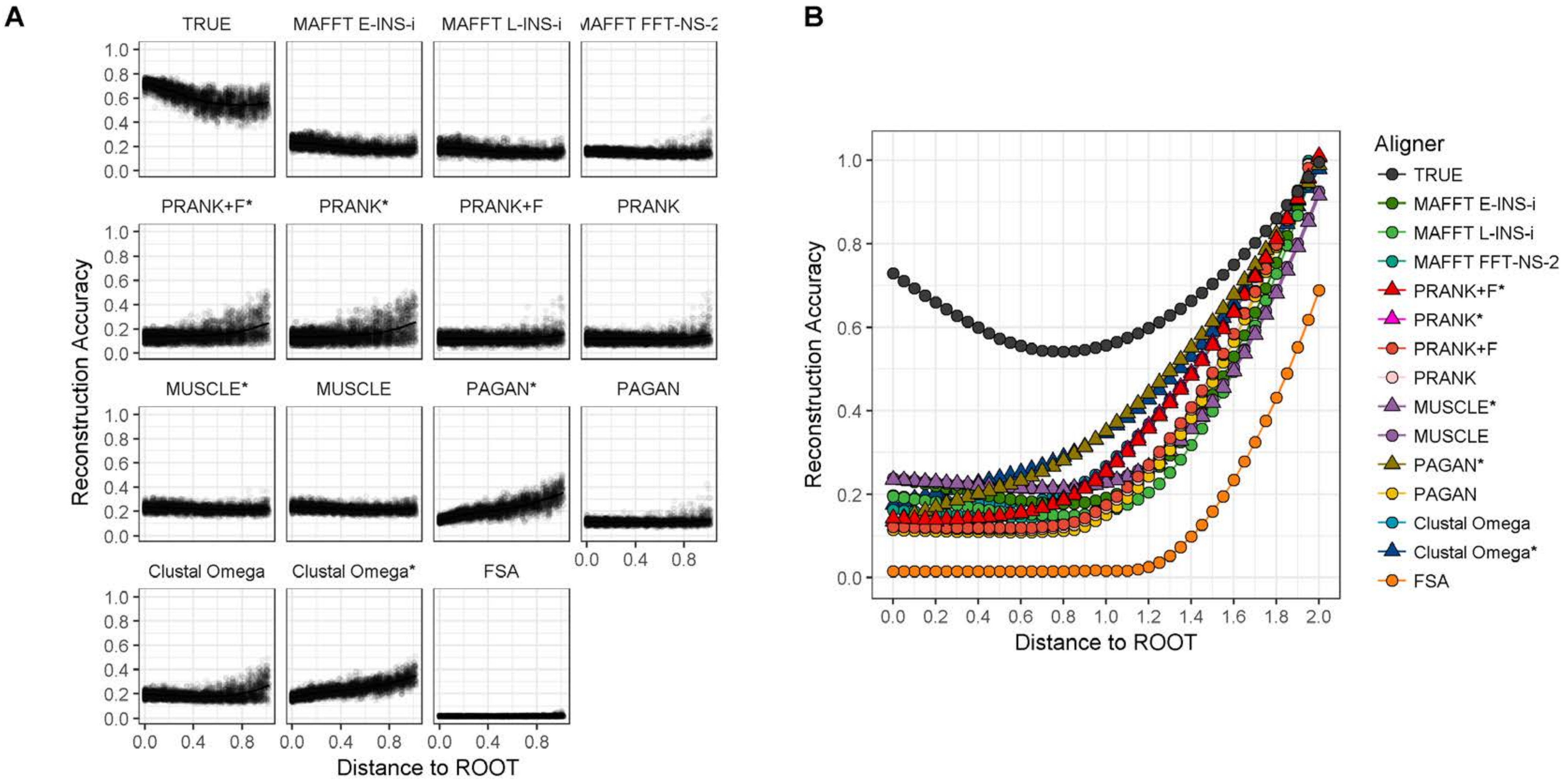

Taxa=64 Tree_height=2 Sampling_fraction=0.25 Indel_rate=0.01 Indel_model=POW 1.7 20

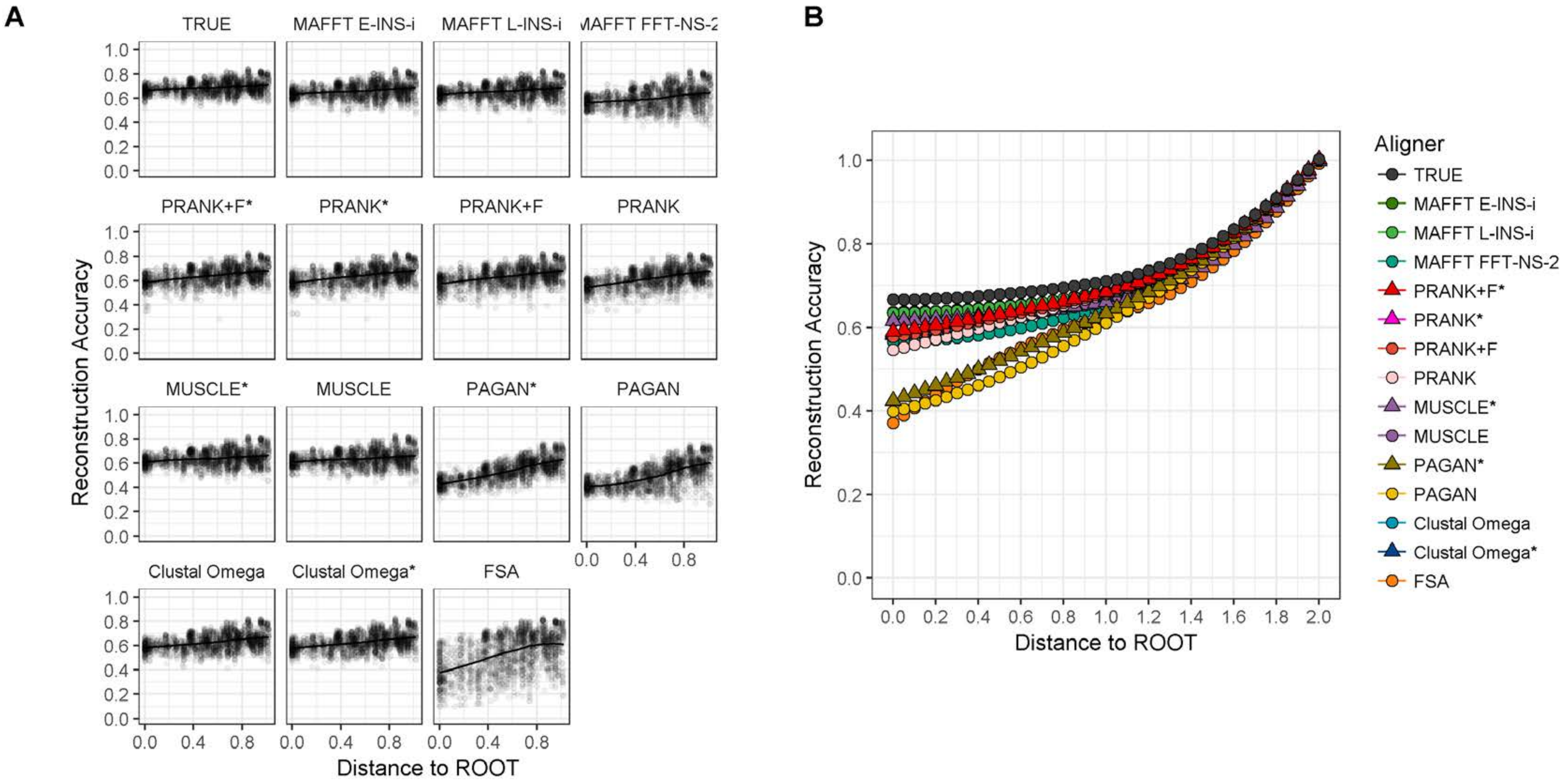

Taxa=64 Tree_height=2 Sampling_fraction=0.25 Indel_rate=0.05 Indel_model=POW 1.7 20

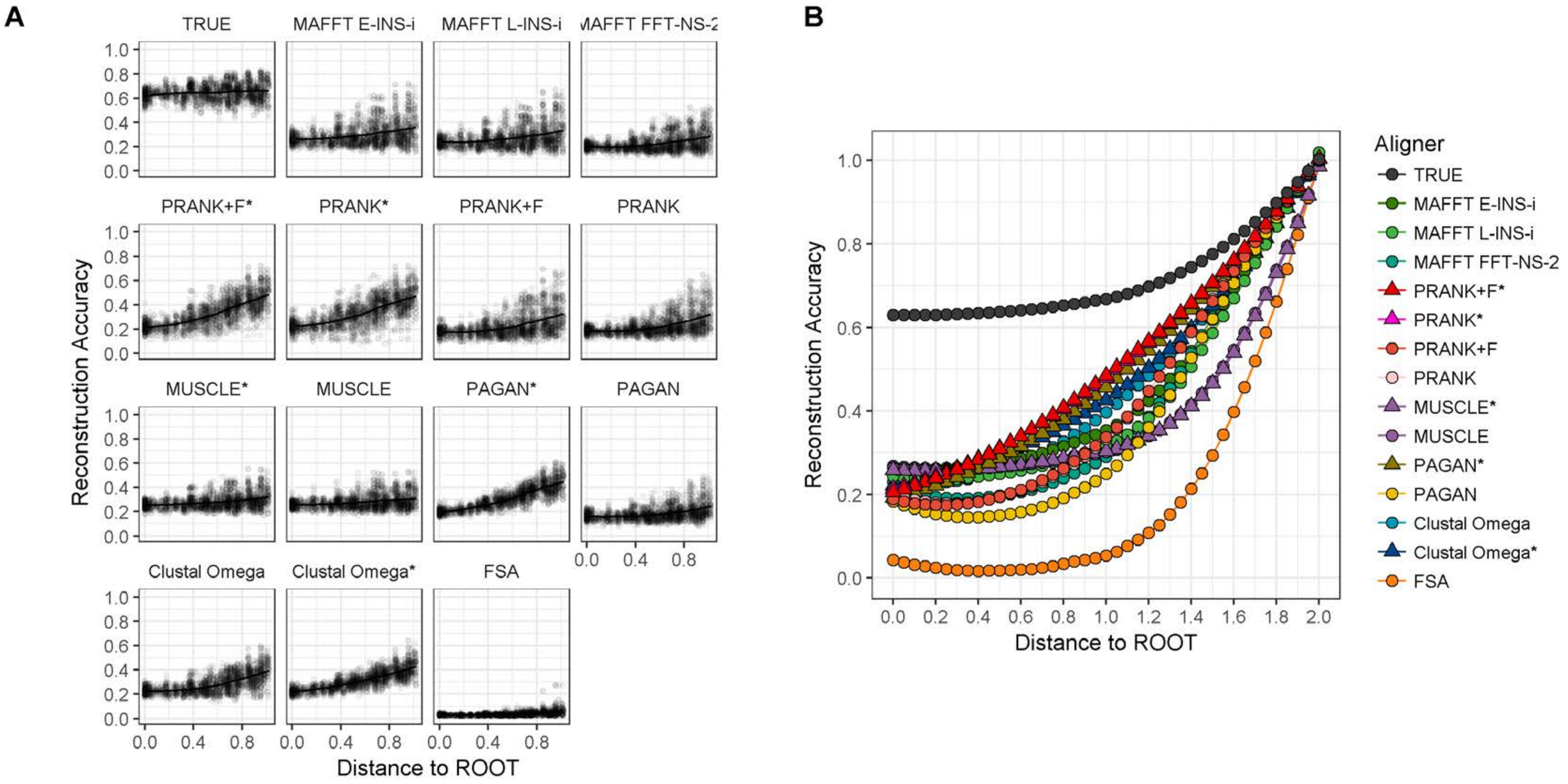

Taxa=64 Tree_height=2 Sampling_fraction=0.99 Indel_rate=0.01 Indel_model=POW 1.7 20

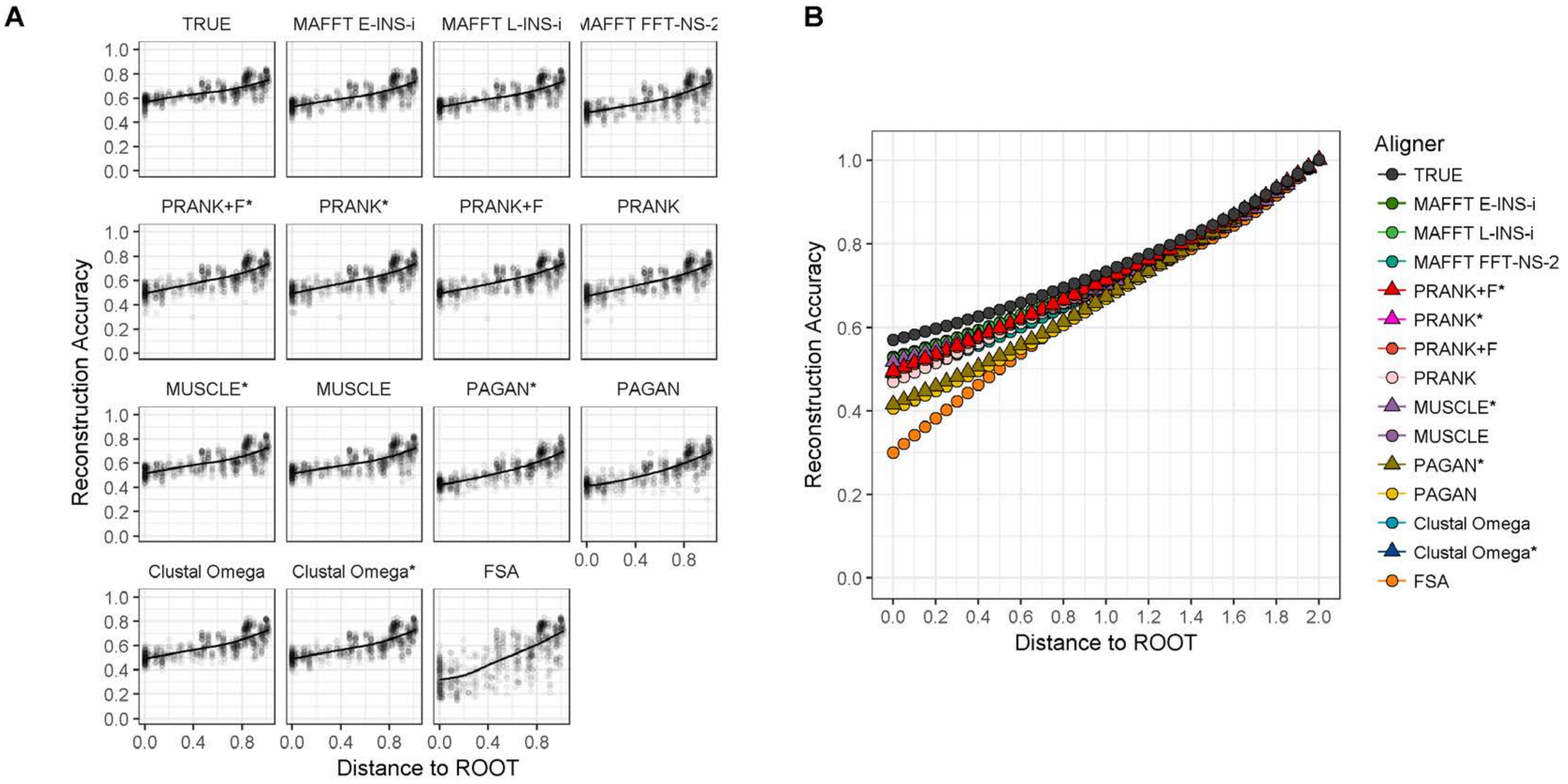

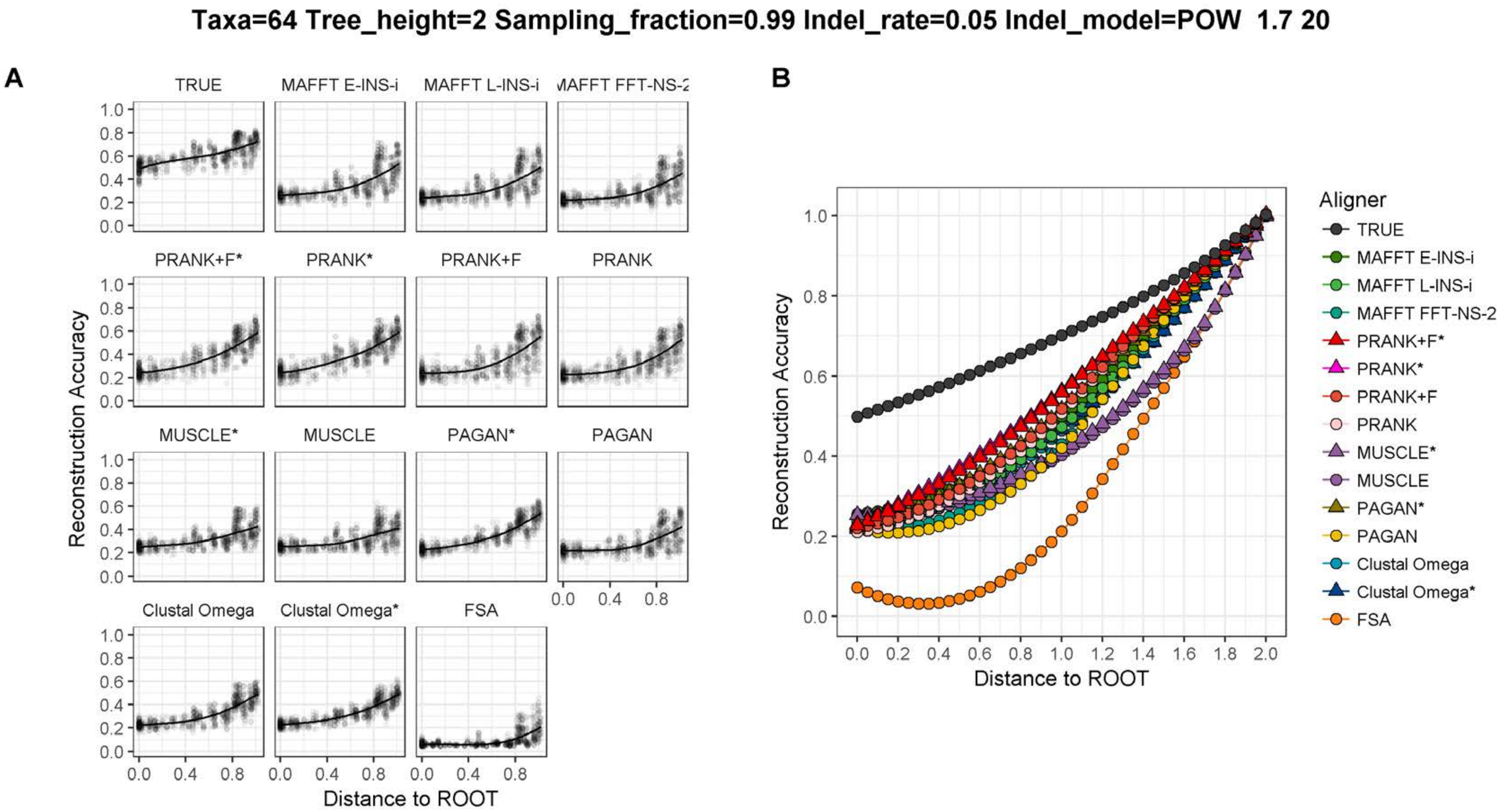
Taxa=64 Tree_height=2 Sampling_fraction=0.99 Indel_rate=0.05 Indel_model=POW 1.7 20
A
TRUE
MAFFT E-INS-i
MAFFT L-INS-i
MAFFT FFT-NS-2
PRANK+F*
PRANK*
PRANK+F
PRANK
MUSCLE*
MUSCLE
PAGAN*
PAGAN
Clustal Omega
Clustal Omega*
FSA
Reconstruction Accuracy
Distance to ROOT
B
Reconstruction Accuracy
Distance to ROOT
Aligner
TRUE
MAFFT E-INS-i
MAFFT L-INS-i
MAFFT FFT-NS-2
PRANK+F*
PRANK*
PRANK+F
PRANK
MUSCLE*
MUSCLE
PAGAN*
PAGAN
Clustal Omega
Clustal Omega*
FSA
