# Supplementary material for: Alignment Modulates Ancestral Sequence Reconstruction Accuracy
Source: Mol Biol Evol. 2018 Apr 3;35(7):1783–97. doi: 10.1093/molbev/msy055 (PMC5995191; doi:10.1093/molbev/msy055)

Taxa=32 Tree_height=1 Sampling_fraction=0.01 Indel_rate=0.02 Indel_model=POW 1.125 50

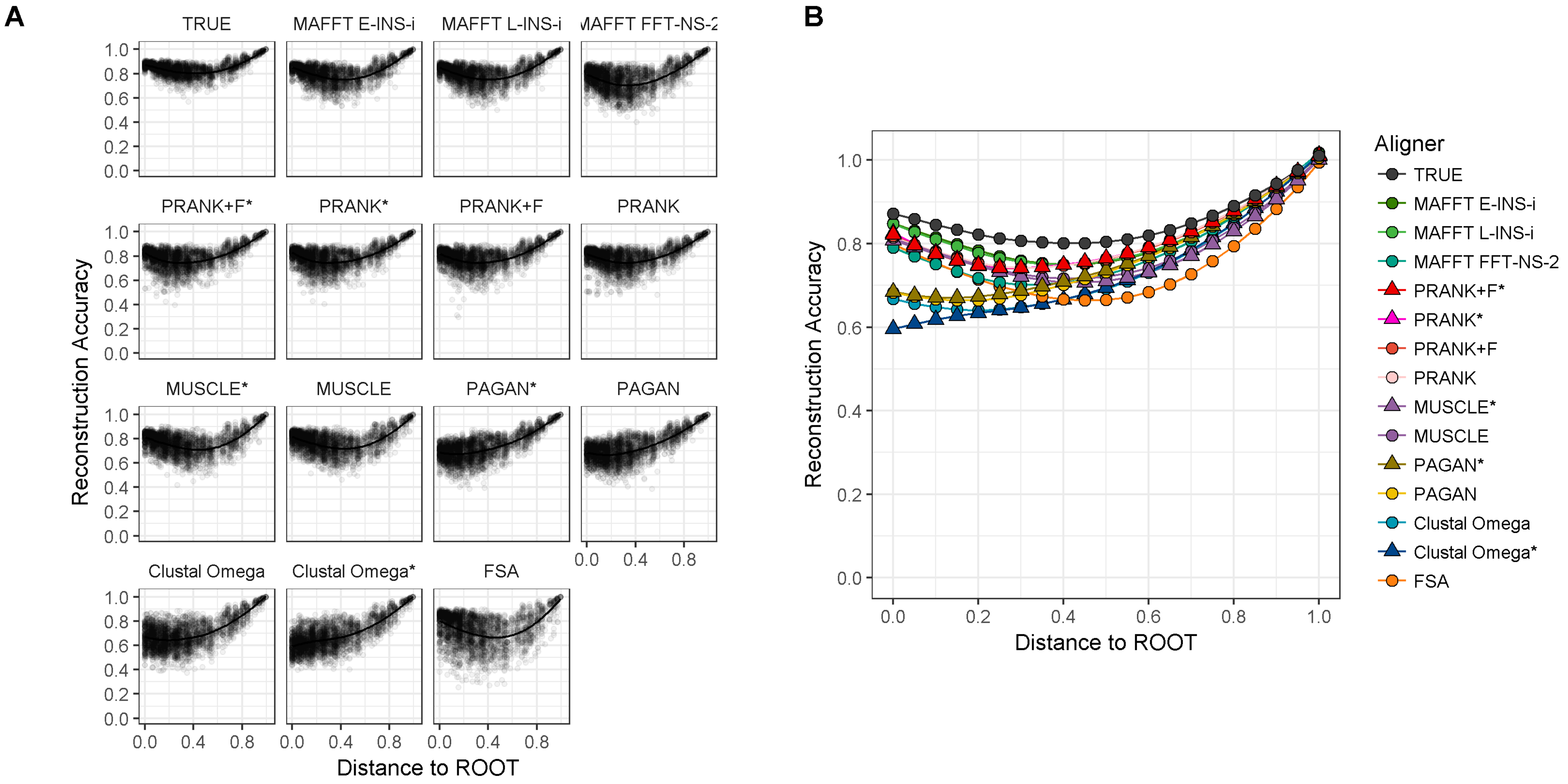

Taxa=32 Tree_height=1 Sampling_fraction=0.99 Indel_rate=0.02 Indel_model=POW 1.125 50

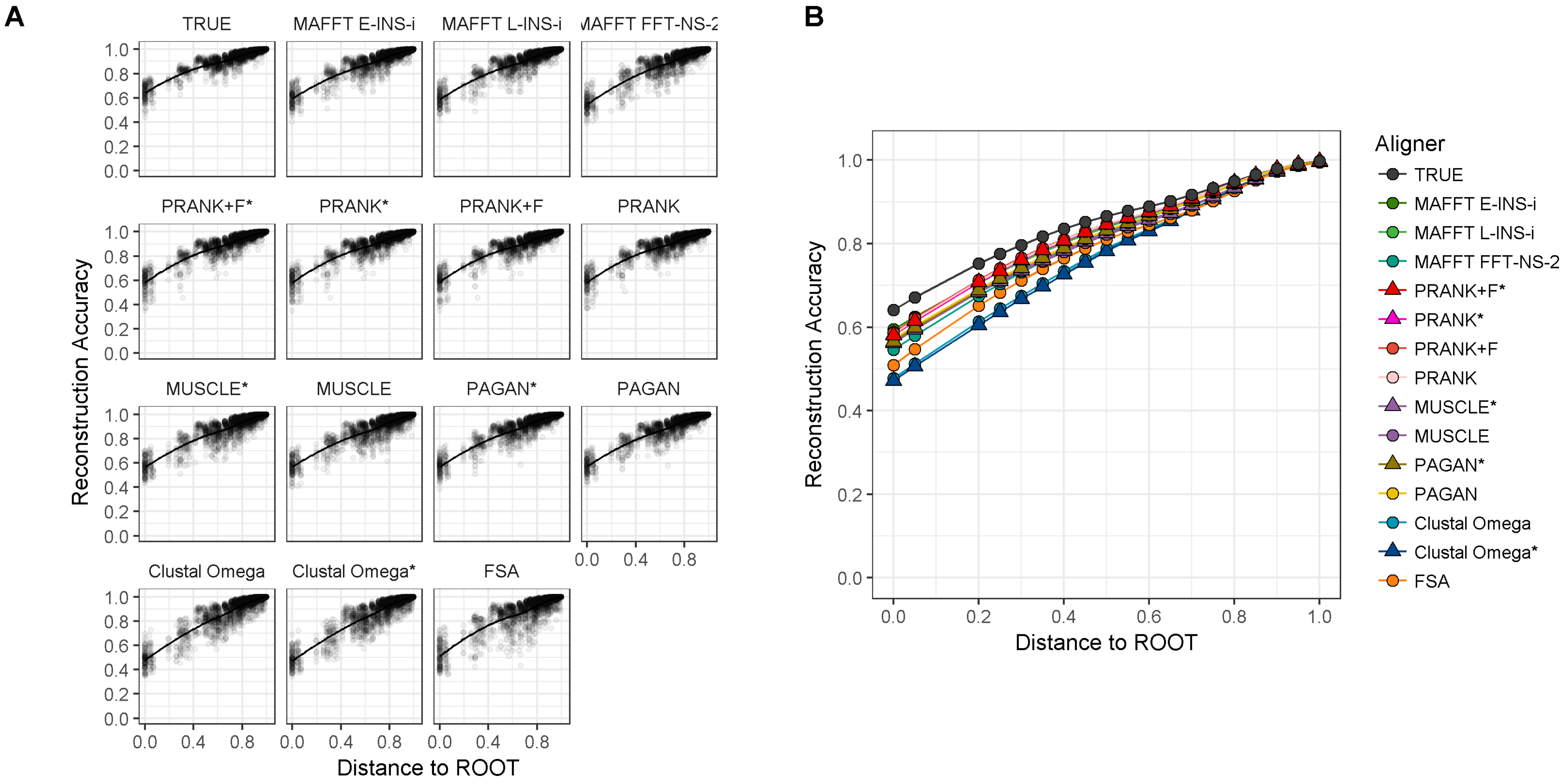

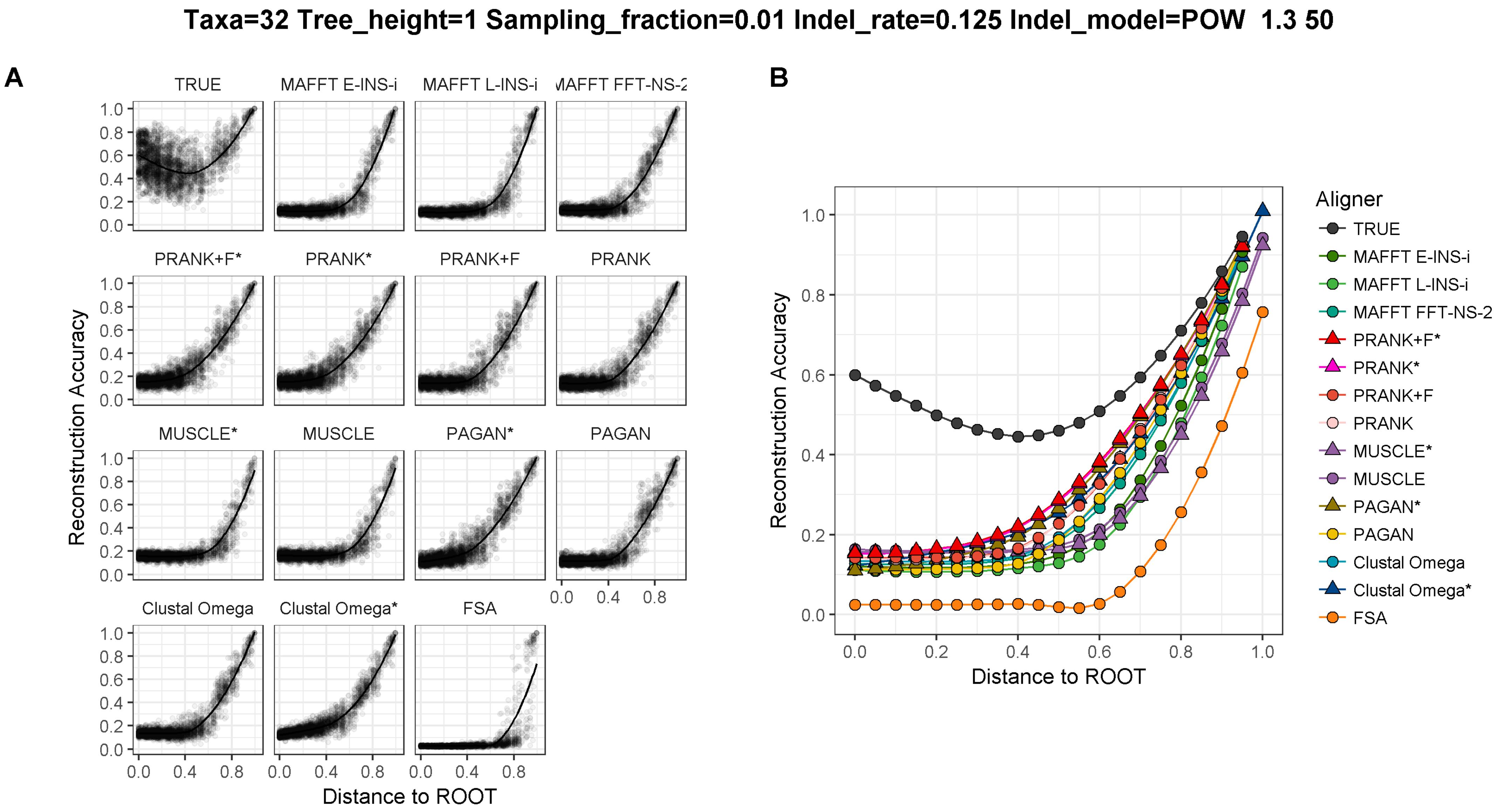
Taxa=32 Tree_height=1 Sampling_fraction=0.01 Indel_rate=0.125 Indel_model=POW 1.3 50
A
TRUE
MAFFT E-INS-i
MAFFT L-INS-i
MAFFT FFT-NS-2
PRANK+F*
PRANK*
PRANK+F
PRANK
MUSCLE*
MUSCLE
PAGAN*
PAGAN
Clustal Omega
Clustal Omega*
FSA
Reconstruction Accuracy
Distance to ROOT
B
Reconstruction Accuracy
Distance to ROOT
Aligner
TRUE
MAFFT E-INS-i
MAFFT L-INS-i
MAFFT FFT-NS-2
PRANK+F*
PRANK*
PRANK+F
PRANK
MUSCLE*
MUSCLE
PAGAN*
PAGAN
Clustal Omega
Clustal Omega*
FSA

Taxa=32 Tree_height=1 Sampling_fraction=0.99 Indel_rate=0.125 Indel_model=POW 1.3 50

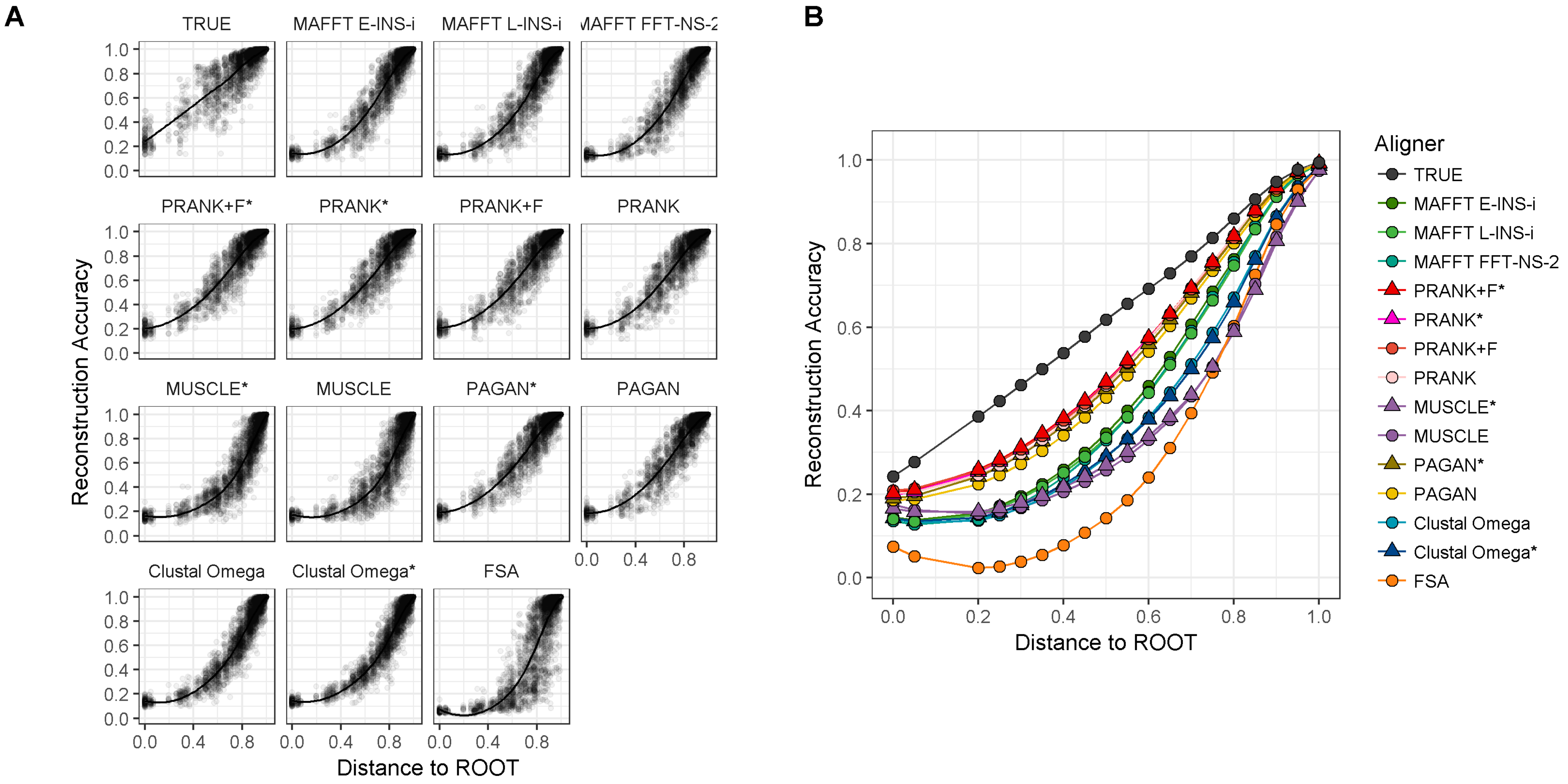

Supplement: Supplementary Data [file msy055_supp.zip › MBE_Vialle_Suppl_Mat_Additional_File_2.pdf]
